# Supplementary material for: What can be learnt from a qualitative evaluation of implementing a rapid sexual health testing, diagnosis and treatment service?
Source: BMJ Open. 2021 Oct 22;11(10):e050109. doi: 10.1136/bmjopen-2021-050109 (PMC8543645; doi:10.1136/bmjopen-2021-050109)
Supplement: Supplementary data [file bmjopen-2021-050109supp001.pdf]

Unity rapid-results service study

Service user interview topic guide v6 (third round)

1. *Introduction and background*

- Thanks, introduce self, re-state purpose of the interview, structure, recording, right to withdrawal
- If participant aged 16 or 17 – explain safeguarding policy and implications for confidentiality
- Check for questions
- Consent: Face to face – record in writing on consent form. Phone – go through consent form & audio record.
- Any relevant demographics not already recorded on the reply slip.

2. *Accessing the service*

- What prompted your visit this time? *(Check if specific concerns and symptoms vs regular check-up)*
- Have you been to the clinic before? *(If so when / how many times?)*
- How did you know about clinic/what did you know in advance about what might happen?
  - How did this affect your decisions / plans? What else would you like to know and how?
- What were you expecting from the service on this occasion? *(Including expectations of treatment, wait etc)*
- How easy was it for you to fit accessing service into your life? *(e.g. take time off, travel etc.)*
  - How acceptable was this, and does it depend on reason for attendance?

3. *Sample drop off phase*

- Did you see a clinician at your first visit? Did you have to wait to be seen? *(How long, was this OK?)*
- What happened when you were called in? *(Info given, any procedures, opportunity to ask questions etc.)*
- Did you take your own samples?
  - How was it? Was it easy to understand how to take them? (*Females – dry swab)*
  - How did you find the process for dropping off your samples?
  - If you have experience of having a clinician take samples before, which would you prefer, and why?
- Females – was contraception discussed?
  - If yes: Raised by you (on form/face to face) or clinician? *(if wanted but not on form why not?)*
    - If advice/services required how were these provided/arranged? *(during apt/follow up?)*
    - Was this acceptable? Was there anything you would have liked to be different?
  - If no: Would you have liked to/was there an opportunity to discuss?
- Were you given information on what tests were for what, and on what would happen next, and was this easy to understand?
- Was there any other information you would have liked at this point?
- On your first visit, did you leave without getting any treatment – was this OK? If not, why not?

4. *Getting results and any treatment or follow up*

- Tell me about receiving your test results *(e.g. wait? How received? In batches? Clarity? How felt about?)*
    - Was this how you were expecting to receive your results? If not, what were your expectations?
    - How would you prefer to receive your results?
- What were the results of your tests?
    - Did this affect your views of the service? *(e.g. would service be more / less acceptable if circumstances different?)*
- Did you have a consultation with a member of staff (apart from drop-off)? Face to face/phone/both?
    - Were your results available at the time of the consultation?
    - How did you find the consultation? *(Including quality of interaction / amount of time with clinician/able to ask questions)*
- Was any treatment or follow up (further tests, further appointments, partner notification) arranged?
- How acceptable would/did you find waiting until follow up appointment for your results (that PM / next day) - to receive treatment? *(instead of being given treatment straight away 'just in case')*
    - Why was this acceptable / not acceptable *(probe re pros/cons, links to reason for attendance)*
    - If not volunteered: Is AMR something which concerns you, and would this influence preferences?
- Would/does this new rapid results service encourage you to test more regularly? Why?
- Were you given all the advice or support you wanted?
- Did you have an opportunity to talk to someone (about any concerns/for advice) during your visit?

5. ***Overall view of the service***

- Overall what do you think has worked well with your visits to Unity?
- What could have improved your experience? Are there any other changes that you could suggest?
- Did the service meet your expectations? Did it provide what you needed?
- Can you think of any positive/negative impacts of organising the service this way? *(e.g. anything off-putting, or more likely to use?)*
- If you have had experience of using sexual health services before the rapid results service started, how did your experience of this service compare? *(Was there anything you preferred about previous service?)*
- What would prompt you to choose this rapid service if you saw it advertised? *(e.g. AMR concerns, quick results, fewer invasive tests?)* Or what explanation of change to service would be acceptable / persuasive to you?

6. ***Any other issues***

- Any other issues the participant would like to raise? Is there anything important I have not asked you about?

Thank them for their time and check preferences regarding receipt of summary of study findings.
