# Supplementary material for: What can be learnt from a qualitative evaluation of implementing a rapid sexual health testing, diagnosis and treatment service?
Source: BMJ Open. 2021 Oct 22;11(10):e050109. doi: 10.1136/bmjopen-2021-050109 (PMC8543645; doi:10.1136/bmjopen-2021-050109)
Supplement: Supplementary data [file bmjopen-2021-050109supp002.pdf]

Unity rapid results service study

Staff interview topic guide (phase 4)

1. ***Introduction and background***

- Thanks, introduce self, re-state purpose of the interview, structure, recording, right to withdrawal.
- Check for questions
- Consent: Phone – go through orally and audio record.

Background information – job/role? How long in this role? Any relevant previous roles/experience? What has your role been in the service changes made in response to COVID-19? Interviewed previously?

From what we understand the Unity service is primarily telephone based now. Walk me through what happens if someone needs a sexual health check. Can you describe the service now we're doing social distancing due to COVID-19?

- Interactions with service users?
- Administration
- Video vs telephone
- Laboratory work
- Interactions /relationships with colleagues?
- Workload?
- Use of panther – drop-off samples and follow-up with patients and accessing treatment
- Postal testing kit requests – use Panther machine now??
- Other?

2. ***Coherence***

What do you think about telephone clinics? What is good/bad about them?

3. ***Cognitive participation***

What training and support did you and/or others receive for running telephone clinics?
Were you involved in the design or delivery of the training?
How engaged were you in the training?
What was good/bad about the training?

4. ***Collective action***

How are telephone clinics working?

- Triage process / involvement of reception staff
- Type / mix of service users seen e.g. symptomatic / asymptomatic, complexity, first/follow up etc.
- Number and duration of consultations
- Content of consultations and history taking
- Number / type of physical examinations you are carrying out?
- How is the process for obtaining samples working? (postal kits through panther machine)
- Getting treatment to patients (Paddy mentioned pick-ups from Boots chemist)
- How is follow-up process working?

How did you find making these changes? Positive or negative? Difficult or easy?
Do you and others in the team feel confident/skilled to do telephone triage?
What is working well? (advantages of having panther machine to do tests in-house / straight forward vs more complex patients – determining who to see in person)
Are there any advantages to the new system? Using Panther for the postal kits (those requested online) - how was this facilitated?
What is more challenging? (not being able to see patients / straight forward vs more complex patients – determining who to see in person)

How are patients responding to the way the service is running now?
Which patients or presentations is this working well for?
Which patients or presentations is this more challenging for?
How do the service design changes impact on the most at risk/vulnerable groups? (service more or less accessible for these groups) E.g. those experiencing domestic violence or who do not have access to phones.
What has helped support the current service changes? (reduced patient numbers, experience of introducing rapid testing / software to link postal tests to patient records)
[IF NOT MENTIONED ABOVE: The service went through a big change introducing panther / rapid testing, do you think having been through that prepared you/ the service for the current situation?

5. ***Reflexive monitoring***
Since starting this new telephone service, has anything had to change to improve it?
How have staff been able to feed back issues which need improvements? Has the way this has been done changed / improved since the new rapid results service was introduced?
What would improve the current telephone service during COVID-19?
What do you think the service will look like after lockdown? (advantages of rapid testing) Continue using postal kits post-lockdown?
Comparing before the rapid-results service was introduced to now, how well is the clinic/are you set up for any potential ongoing social distancing measures in the future? How has introducing the rapid results service helped this? (service is already set up for self-sampling, sample drop off, text results, prescriptions from chemist)

6. ***Any other issues***

- Any other issues the participant would like to raise? Is there anything important I have not asked you about?

Thank them for their time and check preferences regarding receipt of summary of study findings.

Lorenc A, *et al. BMJ Open* 2022; 11:e050109. doi: 10.1136/bmjopen-2021-050109
