# Supplementary material for: What can be learnt from a qualitative evaluation of implementing a rapid sexual health testing, diagnosis and treatment service?
Source: BMJ Open. 2021 Oct 22;11(10):e050109. doi: 10.1136/bmjopen-2021-050109 (PMC8543645; doi:10.1136/bmjopen-2021-050109)
Supplement: Supplementary data [file bmjopen-2021-050109supp003.pdf]

**Elements of rapid STI service implementation**	
**What**	**Who should do this**
**Changes to documentation**	
Rewrite and sign-off treatment guidelines and SOPs when new processes are agreed.	Project leads, clinical lead, project implementation/operations team and clinical governance guideline group
Change the triage process and form.	Project operations team, to include reception staff
Consider changes to IT system/medical records system.	Project leads, consultant and project implementation team
**Implementation of the actual machine and process**	
Write business case for new rapid STI service and have it signed off by PHE.	Clinic lead, clinic manager, lead consultant, project manager, in collaboration with PHE.
Source the machine, find space for it (with waste disposal) and install it. Arrange insurance (including negotiations with PHE and legal teams)	Clinic lead, operations manager, lead consultant, PHE, nursing lead
Ensure IT systems allow direct transfer of data from Panther	Project lead, clinic manager
Pilot before implementing with all patients	All staff including reception teams
Write protocol for Panther outages	Project leads, in collaboration with PHE.
Quality assessment scheme /UKAS accreditation	PHE team
**Services**	
Consider impact on other services	Project operations team
Adjust clinic timetabling to accommodate rapid STI service appointments	Operations manager
Changing medical history forms and process to accommodate the new appointment structure	Project leads and implementation team
Changes to the IT coding	Clinic data manager, project lead, clinic manager, clinical lead (minor)
**Staff engagement, training and communication**	
Put together an implementation team, to oversee implementation, and put in place mechanisms for all staff to feedback to this team	Representative from each staff group and clinic manager.
Clarify communication pathways between all staff and the implementation team	Clinic Manager, project leads
Consider the impact on staff roles and workload and if staffing changes are therefore needed	Project operations team
Regular meetings for staff involved in the new service	Possible staff to include: project leads, HAs, consultants, nursing assistants, nurses, administrative staff, researchers, IT lead, clinic manager, data manager, chlamydia screening program team lead.
Staff training and regular updates at existing staff training sessions	Led by project leads, all staff to attend
Regular departmental meetings	Project leads and clinical lead
**Patient communication**	
Communicate changes to patients – write leaflets/posters/website	Project leads
