# Supplementary material for: What can be learnt from a qualitative evaluation of implementing a rapid sexual health testing, diagnosis and treatment service?
Source: BMJ Open. 2021 Oct 22;11(10):e050109. doi: 10.1136/bmjopen-2021-050109 (PMC8543645; doi:10.1136/bmjopen-2021-050109)
Supplement: Author's manuscript [file bmjopen-2021-050109.draft_revisions.pdf]

BMJ Open

BMJ Open is committed to open peer review. As part of this commitment we make the peer review history of every article we publish publicly available.

When an article is published we post the peer reviewers' comments and the authors' responses online. We also post the versions of the paper that were used during peer review. These are the versions that the peer review comments apply to.

The versions of the paper that follow are the versions that were submitted during the peer review process. They are not the versions of record or the final published versions. They should not be cited or distributed as the published version of this manuscript.

BMJ Open is an open access journal and the full, final, typeset and author-corrected version of record of the manuscript is available on our site with no access controls, subscription charges or pay-per-view fees (http://bmjopen.bmj.com).

If you have any questions on BMJ Open's open peer review process please email info.bmjopen@bmj.com

BMJ Open

What can be learnt from a qualitative evaluation of implementing a rapid sexual health testing, diagnosis and treatment service?

Journal:	*BMJ Open*
Manuscript ID	bmjopen-2021-050109
Article Type:	Original research
Date Submitted by the Author:	10-Feb-2021
Complete List of Authors:	Lorenc, Ava; University of Bristol, Population Health Sciences, Bristol Medical School; National Institute for Health Research, Applied Research Collaboration West<br>Brangan, Emer; University of the West of England, Department of Nursing and Midwifery<br>Kesten, Joanna; University of Bristol, National Institute for Health Research, Applied Research Collaboration West (NIHR ARC West); University of Bristol, NIHR Health Protection Research Unit in Behavioural Science and Evaluation, Population Health Sciences, Bristol Medical School<br>Horner, Paddy; University of Bristol, NIHR Health Protection Research Unit in Behavioural Science and Evaluation, Population Health Sciences, Bristol Medical School; University Hospitals Bristol and Weston NHS Foundation Trust, Unity Sexual Health<br>Clarke, Michael; University Hospitals Bristol and Weston NHS Foundation Trust, Unity Sexual Health<br>Crofts, Megan; University Hospitals Bristol and Weston NHS Foundation Trust, Unity Sexual Health<br>Steer, Jonathan; Public Health England, South West Regional Laboratory, National Infection Service<br>Turner, Jonathan; Public Health England, South West Regional Laboratory, National Infection Service<br>Muir, Peter; Public Health England, South West Regional Laboratory, National Infection Service<br>Horwood, Jeremy; University of Bristol, National Institute for Health Research, Applied Research Collaboration West (NIHR ARC West), University Hospitals Bristol and Weston NHS Foundation Trust; University of Bristol, NIHR Health Protection Research Unit in Behavioural Science and Evaluation, Population Health Sciences
Keywords:	QUALITATIVE RESEARCH, SEXUAL MEDICINE, Organisational development < HEALTH SERVICES ADMINISTRATION & MANAGEMENT, Change management < HEALTH SERVICES ADMINISTRATION & MANAGEMENT, COVID-19

For peer review only - http://bmjopen.bmj.com/site/about/guidelines.xhtml

I, the Submitting Author has the right to grant and does grant on behalf of all authors of the Work (as defined in the below author licence), an exclusive licence and/or a non-exclusive licence for contributions from authors who are: i) UK Crown employees; ii) where BMJ has agreed a CC-BY licence shall apply, and/or iii) in accordance with the terms applicable for US Federal Government officers or employees acting as part of their official duties; on a worldwide, perpetual, irrevocable, royalty-free basis to BMJ Publishing Group Ltd ("BMJ") its licensees and where the relevant Journal is co-owned by BMJ to the co-owners of the Journal, to publish the Work in this journal and any other BMJ products and to exploit all rights, as set out in our licence.

The Submitting Author accepts and understands that any supply made under these terms is made by BMJ to the Submitting Author unless you are acting as an employee on behalf of your employer or a postgraduate student of an affiliated institution which is paying any applicable article publishing charge ("APC") for Open Access articles. Where the Submitting Author wishes to make the Work available on an Open Access basis (and intends to pay the relevant APC), the terms of reuse of such Open Access shall be governed by a Creative Commons licence – details of these licences and which Creative Commons licence will apply to this Work are set out in our licence referred to above.

Other than as permitted in any relevant BMJ Author's Self Archiving Policies, I confirm this Work has not been accepted for publication elsewhere, is not being considered for publication elsewhere and does not duplicate material already published. I confirm all authors consent to publication of this Work and authorise the granting of this licence.

For peer review only

What can be learnt from a qualitative evaluation of implementing a rapid sexual health testing, diagnosis and treatment service?

Authors

Ava Lorenc[1,2], Emer Brangan[1,3], Joanna M Kesten[1,2,4], Patrick Horner[1,4,5], Michael Clarke[5], Megan Crofts[5], Jonathan Steer[6], Jonathan Turner[6], Peter Muir[6], Jeremy Horwood[1,2,4]

[1.] Population Health Sciences, Bristol Medical School, University of Bristol, Canynge Hall, 39 Whatley Road, Bristol, BS8 2PS

[2.] National Institute for Health Research, Applied Research Collaboration West (NIHR ARC West), University Hospitals Bristol and Weston NHS Foundation Trust, 9th Floor, Whitefriars, Lewins Mead, Bristol, BS1 2NT

[3] University of the West of England
Department of Nursing and Midwifery, Faculty of Health and Applied Sciences, University of the West of England, Frenchay Campus, Coldharbour Lane, Bristol, BS16 1QY

[4] NIHR Health Protection Research Unit in Behavioural Science and Evaluation, Population Health Sciences, Bristol Medical School, University of Bristol, Canynge Hall, 39 Whatley Road, Bristol, BS8 2PS

[5] Unity Sexual Health, University Hospitals Bristol and Weston NHS Foundation Trust, Tower Hill, Avon, Bristol BS2 0JD

[6] Public Health England South West Regional Laboratory, National Infection Service, Pathology Sciences Building, Science Quarter, Southmead Hospital, Bristol BS10 5NB

Corresponding author: Jeremy Horwood, J.Horwood@bristol.ac.uk

Word count: 3993

For peer review only

Abstract

Objectives

To investigate experiences of implementing a new rapid sexual health testing, diagnosis and treatment service.

Design

A theory-based qualitative evaluation with a focused ethnographic approach using non-participant observations and interviews with patient and clinic staff. Normalisation Process Theory was used to structure interview questions and thematic analysis.

Setting

A sexual health centre in Bristol, UK.

Participants

26 patients and 21 staff involved in the rapid sexually transmitted infection (STI) service were interviewed. Purposive sampling aimed for a range of views and experiences and socio-demographics and STI results for patients, job grades and roles for staff. 40 hours of observations conducted.

Results

Implementation of the new service required co-ordinated changes in practice across multiple staff teams. Patients also needed to make changes to how they accessed the service. Multiple small 'pilots' of process changes were necessary to find workable options. For example, the service was introduced in phases beginning with male patients. This responsive operating mode created challenges for delivering comprehensive training and communication in advance to all staff. However, staff worked together to adjust and improve the new service, and morale was buoyed through observing positive impacts on patient care. Patients valued faster results and avoiding unnecessary treatment. Patients reported that they were willing to drop-off self-samples and return for a follow-up appointment, enabling infection-specific treatment in accordance with test results thus improving antimicrobial stewardship.

Conclusions

The new service was acceptable to staff and patients. Implementation of service changes to improve access and delivery of care in the context of stretched resources can pose challenges for staff at all levels. Early evaluation of pilots of process changes, played an important role in the success of the service by rapidly feeding back issues for adjustment. Visibility to staff of positive impacts on patient care is important in maintaining morale.

Strengths and limitations of this study

- The 'trial, assess, adapt' strategy (reflexive process of observation, feedback, and resulting action) meant that evaluation and implementation occurred in parallel and allowed researchers to capture the active process.
- The evaluation benefitted the staff, as researchers provided ongoing feedback and suggestions for service improvements and provided a space for reflection.

For peer review only - http://bmjopen.bmj.com/site/about/guidelines.xhtml

For peer review only

- A strong and trusting relationship between research and clinic staff arose from researcher flexibility and timely responsiveness and allowed good researcher access to spaces, staff and meetings.
- Frequent, regular and extensive physical presence of the researcher in various clinic settings was crucial as much of the process was not documented.
- The patient sample was limited due to recruitment being cut short by the COVID-19 pandemic lockdown, and we only interviewed males due to the pathway being initially implemented for male patients during the evaluation period.

For peer review only

For peer review only - http://bmjopen.bmj.com/site/about/guidelines.xhtml

Introduction

Rates of sexually transmitted infections (STIs) continue to increase in England despite control efforts, with a 5% increase between 2018-2019[1]. *Chlamydia trachomatis* (chlamydia) and *Neisseria gonorrhoeae* (gonorrhoea) are the most common, with 226,411, and 70,982 diagnoses reported in England in 2019, a 5% and 26% increase since 2018[2]. The rise in gonorrhoea is particularly concerning as first line treatment effectiveness is threatened by the development of antimicrobial resistance (AMR)[3]. Most STIs are diagnosed through Specialist Sexual Health Services (SSHS), the provision of which is increasingly challenging as funding (via government public health grant), has been steadily cut since 2015[6].

Chlamydia and gonorrhoea if left untreated may cause pelvic inflammatory disease (PID) in women, which can result in infertility, ectopic pregnancy, and chronic pelvic pain[7-9]. Infections are often asymptomatic, particularly in women, and when they do cause symptoms and/or signs these are not pathognomonic[7 8]. Nucleic acid amplification tests (NAATs) provide accurate detection. Early detection and treatment helps prevent the spread of STIs and the development of complications. Point-of-care testing (POCT; results within 15-30mins)[10] and rapid STI services (results on the same day) can potentially improve care and reduce costs, due to reduced time from diagnosis to treatment and number lost to follow up. This can increase testing uptake, improve partner notification rates and enable better and timelier clinician decisions, improving outcomes such as fewer unnecessary treatments and reduced PID risk[11-14]. Patients prefer rapid STI testing[15-17] and are happy to wait at clinic for results. Rapid testing can reduce anxiety[18 19] and improve patient acceptability of services and uptake of testing[20-23]. HIV POCT is well established and preferred by high risk men who have sex with men (MSM)[24 25]. Although studies suggest a limit of 30 minutes to wait for results[26-29], experience from our service indicates patients would be prepared to wait longer than 20 minutes for their result[30].

However, much of the evidence is from modelling and hypothetical views of clinicians and/or patients[11-13 26-29 31], with little real-life implementation evaluation, and rarely considering the complexity of patient visits including both asymptomatic and symptomatic patients with multiple needs e.g. female contraception. There is an urgent need to evaluate staff and patient preferences, and clinical benefits and cost effectiveness in practice.

In November 2018, a UK SSHS implemented a first-of-its-kind rapid STI testing, diagnosis and treatment service, using a clinic-based Hologic 'Panther' NAAT diagnostic machine. In 2017, the clinic introduced an online STI and HIV testing postal service for asymptomatic patients[32]. The new rapid service provides chlamydia and gonorrhoea results in 3.5 hours (previously over a week when tested in the microbiology laboratory), to improve patient care while reducing costs. This evaluation assessed the best service model and patient and staff acceptability, to refine and improve the service and support implementation in other SSHSs. We report the qualitative evaluation of male patient and staff views and experiences of the implementation of the first phase of this new rapid STI service.

For peer review only - http://bmjopen.bmj.com/site/about/guidelines.xhtml

Methods

Design

The evaluation was ethnographic, theory-based (informed by Normalisation Process Theory (NPT)) and used observations and interviews[33]. The study focussed on four timepoints during 16 months of evaluation: T1 at start of implementation; T2 after 6 months; T3 after 14 months; T4 at 16 months during the COVID-19 pandemic lockdown.

Setting

A sexual health clinic in Bristol (population 450,000), UK.

Participants

Due to the new services being initially introduced for the male pathway only, male patients (over 16 years old) and staff at the sexual health clinic were interviewed. Patients were invited to take part, via a clinic survey about PrEP (pre-exposure prophylaxis for HIV) [34] and physically attending the clinic at T2 and T3. Purposive sampling attempted to capture maximum variation in views and experiences, and socio-demographics and STI test results for patients, and job grades and roles for staff (consultants, doctors, nurses/nursing assistants, health advisers, Public Health England (PHE); responsible for the Panther lab and admin). Information sheets were provided to male patients by staff at the clinic or via email from researchers, with patients asked to contact the researcher and ask questions before deciding to take part. Staff were emailed by the researcher about the study.[35] with continuous assessment of information within our sample with regard to meeting study objectives.

Data collection

In the first 6 months of service implementation, observations were conducted at varying times/days, in reception, laboratory and waiting areas. Non-participant observations focussed on day-to-day operations, how clinic staff integrated the new service and any factors which promoted or inhibited successful incorporation, providing insights into peoples' views and actions via the contexts and locations they inhabited[36]. Written accounts included observations, conversations, and reflection[37].

Staff were interviewed in four batches at each timepoint. Interviews explored: views and experiences of the service; impact on workload and clinical practice; information and support needs, sustainability and future implementation of the service. Patient interviews took place throughout the evaluation period and explored their experience and views of the service including acceptability, barriers and facilitators to uptake. Patients were offered a £10 High Street shopping voucher. Interviews were conducted by experienced qualitative senior research associates AL/JMK/EB, used flexible topic guides and open-ended questioning, were face-to-face (at the clinic or University) or by telephone, and lasted around 30 minutes. Participants were told that the study was evaluating the rapid results service and that interviewers were independent of the service.

Analysis

Interviews were audio recorded, transcribed verbatim and imported into QSR NVivo (version 10). Ongoing and iterative analysis informed further data collection and service development, evaluation,

For peer review only - http://bmjopen.bmj.com/site/about/guidelines.xhtml

adaptation, refinement and integration. Thematic, inductive analysis by EB/AL identified and analysed patterns and themes salient to participants. Initial noting of ideas was followed by line-by-line examination and inductive coding. A subset of transcripts were independently double-coded by EB/JH and discrepancies discussed. Negative cases and reasons for deviance were explored. The four NPT constructs[38] were used to further develop themes. NPT proposes that successful implementation of an intervention is dependent on participants ability to fulfil four criteria : 1) Coherence - (sense-making - understanding and opinion of the intervention purpose); 2) Cognitive Participation (commitment and engagement with the intervention); 3) Collective Action (the work that individuals and organisations have to do to make the intervention function); 4) Reflexive Monitoring (appraisal of the intervention once it is in use).

Ethical approval

South West Frenchay Research Ethics Committee granted approval, reference 18/SW/0090.

Patient and Public Involvement

PPI meetings with three people who recently used the clinic informed the study design. These meetings reviewed patient-facing materials and discussed the acceptability of proposed recruitment and data collection.

Results

Participants/hours of observation

25 observations were conducted, approximately 40 hours total, 25 staff interviews (24 participants), 26 patient interviews. Patients ranged in age from 19 to 57, average 34 years, index of multiple deprivation scores ranged from 2 to 10, average 5.4, and most identified as MSM. Two had positive STI test results.

Coherence (sense-making)

Staff and patients welcomed rapid testing (Table 1). All staff saw it as beneficial and many were excited about doing something new, particularly to improve service access which was limited by a lack of pre-bookable appointments, high observed demand (manifesting in long queues outside before the clinic opened each morning to access limited capacity walk-in appointments) and staff shortages. Some staff had concerns around anticipated reduced clinician contact and shorter consultations in the new service. Patients most valued a potentially quicker and more convenient service, but also reduced anxiety from waiting for results, which may increase testing frequency. Staff also welcomed being able to provide treatment based on results and avoiding unnecessary antibiotic prescribing (previously treatment was prescribed presumptively for symptomatic patients due to the week-long wait for test results). Some patients valued avoiding unnecessary antibiotic treatment on a personal basis and acknowledged wider societal issues.

Cognitive participation (buy in)

The importance of engaging the whole clinic team in the service redesign was recognised but challenging with a large team and many part-time staff (Table 1). Formal engagement was via an implementation team, project meetings and staff training sessions. Engagement of ‘on the ground’ staff was inadequate, with admin and nursing staff feeling particularly disengaged and having limited

For peer review only - http://bmjopen.bmj.com/site/about/guidelines.xhtml

For peer review only

preparation time, citing little communication (due to busy work schedules with limited time for accessing emails), training (lack of access as many staff were part-time and did not work on the day training was delivered) or involvement in project meetings or the implementation group (which was initially only senior staff, although this did improve).

Engagement was also limited by a lack of protected project time – implementation work was fitted around existing high workloads, rapid changes made timely feedback difficult, and burn out from staff pressures (funding cuts, understaffing, and high service demand).

Collective action (putting rapid STI test results service into operation)

The service was implemented for males in November 2018 and for all in August 2019. The changes to the patient pathway and quotes relating to collective action are listed in Table 2 and 3 respectively. Some of these caused challenges:

- After registering, eligible patients wait at clinic to be seen within the first hour of session, rather than coming back hours or days later to a "slot" on the walk-in clinic.

- The shorter initial appointments, with reduced medical record completion and fewer physical examinations, was a 'huge change' and source of concern and anxiety for clinicians both before and during the changes, due to perceived loss of opportunities for patient discussions e.g. about domestic violence, female genital mutilation, alcohol use, and contraception, seen as essential for a 'holistic', 'integrated' 'level 3 service'. This did improve with practice, and patients with particularly concerning issues were referred for a health adviser consultation, which was longer under the new service.

- Self-sampling drop-off meant reduced clinical contact, particularly for asymptomatic, low-risk men with negative test results (health advisers only see high-risk/new MSM patients at the first visit). The walk-in clinic was therefore more demanding, as the case mix changed, seeing more symptomatic patients and with complex presentations. Although reduced clinical contact with asymptomatic patients was a planned cost-saving benefit, nursing assistants (running the sample drop-off sessions) often ended up collecting mandatory data (GUMCAD surveillance system[39]) and answering patient clinical queries, which they were not qualified/paid/willing to do.

- Chlamydia and gonorrhoea treatments were to be given based on results, not presumptively unless sexual contact with a case was within the 2 weeks window period and patient requested treatment[7 8]
    - Men with symptoms of urethritis were first tested for chlamydia/gonorrhoea and booked to return more than 4 hours later. If NAAT-positive they were treated according to British Association for Sexual Health and HIV (BASHH) chlamydia and gonorrhoea guidelines[7 8] and if negative tested for urethritis and managed according to BASHH guidelines[40] [with reassurance, including a leaflet, if negative] and told to re-attend for an early morning smear if their symptoms did not resolve. Some patients, particularly regulars, were not keen on this longer wait for treatment, although this did improve.
    - A minority of clinicians deviated from protocol and treated presumptively, especially for patients who were particularly anxious. Staff reported mixed patient understanding of only treating when results were available, with detailed explanations needed, but patients were amenable once they understood.

Important in collective action was **designing and documenting** the new patient pathways, which needed to be clear but flexible, responding to individual patient situations and need e.g. anxiety,

For peer review only - http://bmjopen.bmj.com/site/about/guidelines.xhtml

medical history, relationship status, availability to attend clinic, with some staff deviation from protocol. Guidelines, SOPs (Standard Operating Procedures) and pathways had to be rewritten. However, detailed SOPs were not always in place prior to implementation of a new modification to a pathway, making it difficult for staff to keep up with current processes. This was due to the repeated and frequent changes to clinic processes/patient pathways, and the lack of protected admin time which meant that when patient pathways were revised following staff feedback these could take over 6 weeks to review and be signed off by the clinical governance group. The triage form for patients to self-identify at reception whether symptomatic, their risk level, and if they had had sex against their will was revised three times during implementation to make it clearer which pathway should be followed. This was stressful for reception staff, particularly given their limited engagement and training. Observation showed that they annotated a copy of the triage form to remind them of the pathways for different responses.

Patients were happy with **communication** about the changes made (via the website, staff, consultations, on the triage form clipboard).

The implementation process was one of **continual adaptations**. Although staff accepted this as inevitable due to the novelty of the service, it was difficult. During initial implementation, '**teething issues**' were experienced, including administration staff not knowing which patients were eligible for the service, dealing with the high volume of patients when the doors first open, and the best way to triage patients.

The responsive model meant comprehensive preparatory **training** and **communication** to all staff was challenging, although communication challenges were not unique to this project. Multiple methods of communication were essential. The evaluation process aided communication, and researchers were able to suggest solutions to problems based on the non-participant observation. For example, researcher (EB) co-developed with the clinical team a laminated card for patients explaining the new service in response to the researcher observation that patients were given variable information by reception staff. However, staff meetings were often poorly attended as many staff were part-time and these were held at a fixed time each week. Lack of training was particularly noticeable and stressful for administrative staff, much of which was 'on the job'.

Understaffing (a chronic problem in the NHS) and extra workload (due to both the new system and increased demand), affected implementation, caused much stress, and aggravated the teething problems. This applied to all teams, but particularly reception staff. There were ongoing budget cuts and lack of funding during the implementation period.

Many staff found changing ingrained **behaviours** difficult, particularly reducing the content and duration of consultations when they had been taught to maximise patient contact.

Staff worked together to adjust and improve the new service, identifying problems and opportunities and innovating in their own practice, overseen and supported by the implementation groups, and morale was buoyed by the positive impact on patient care and the positive feedback from the research team.

Reflexive monitoring (appraisal of STI test results service into operation)

Contextual factors

Contextual factors influencing the service experience included: inadequate service funding; understaffing; ongoing communication problems; increased use of postal testing (less complex

For peer review only - http://bmjopen.bmj.com/site/about/guidelines.xhtml

patients use postal testing so more complex at walk-in); increasing use of PrEP; increasing service demand; and increasing societal awareness of gender issues. Issuing triage forms to male patients on arrival created tensions around sensitively managing patients who did not identify as either male or female including trans patients, this process was amended following feedback from the research team.

Success

Overall, the new service was seen as successful as it was implemented and running fairly smoothly after initial teething problems (Table 4). Although the process was challenging, implementation was an achievement, given the constraints on resources and staffing and lack of additional funding. Staff were credited with being adaptable, highly motivated, hardworking and mutually supportive. They were proud of being part of something new. The evaluation process played an important role in the success of the service by rapidly feeding back issues for resolution.

Patients rated the quality of the new service highly, with some patients specifically requesting it. There was interest/enthusiasm from other UK SSHS.

Although staff were initially concerned that the changes would jeopardise the high quality of care, this does not appear to have been realised, with patients very positive about staff and the ability to raise concerns and discuss issues.

Benefits

Decreased time to diagnosis and treatment meant less patient anxiety while waiting for results. Self-testing and less physical examinations involving invasive sampling (urethral swab) was generally preferred by patients. Staff perceived that the service was able to see more patients, and that clinicians and health advisers could spend more time and better engage with complex and higher risk patients due to more efficient processing of patients with straightforward needs. Staff, and some patients, were pleased to be able to treat with results, which promoted informed discussions and reduced antibiotic use, secondary complications, and onward transmission. Most patients were happy to wait up to 48 hours for treatment.

Staff satisfaction was boosted from doing something new and exciting and achieving implementation, contributing to enhanced teamwork and coherence. Staff reported improvements in their work experience and job satisfaction, mainly the improvements to consultations with patients, including consultants seeing more complex patients. These boosts to staff satisfaction and morale, gave the team confidence that they could make further service improvements, demonstrated by the changes made during the COVID-19 pandemic during which staff reported being more 'change-ready'.

Suggested improvements

For many staff the most important implementation improvements were preparation of documentation and engaging and communicating (especially face-to-face) with all staff but particularly nursing and reception. It was also recommended that, if possible, staff needed to be better prepared for behavioural change and multiple continual adaptations, and given protected time for the project, and the impact on staff roles and workloads better considered. Other areas for improvement were: consistency in the rapidity of results and contingency planning for malfunctions (sometimes results were not available on time due to Panther breakdowns); more and earlier information for patients, especially on the process and timings (waiting times, results notification etc).

For peer review only - http://bmjopen.bmj.com/site/about/guidelines.xhtml

Based on our data, suggested improvements to the service changes are:

- Document new processes and pathways as soon as possible.
- Engage and involve staff at all levels and with direct experience in each relevant area of work, as well as management and leadership roles, to improve process design iterations.
- Small-scale pilots of the new service with patients, to test and refine draft processes to reduce staff stress and confusion.
- Bring teams together for training to facilitate information exchange and understanding.
- Supportive communication from senior staff, and a variety of methods for communication including written, training sessions, on-the-job support, informal, and nominated individuals for support.
- Consider the impact of the changes on staff roles and workload.
- Consider wider use of phone/video clinics, which were implemented during physical distancing requirements of COVID-19, but may have benefits elsewhere.

The supplementary file summarises service considerations for implementing a rapid STI service, and relevant teams/job roles.

Discussion

Principal findings

The first UK rapid NAAT testing integrated SSHS for chlamydia and gonorrhoea was successfully implemented despite funding and staff shortages. Inevitable teething glitches were resolved and, overall, it was well received. Staff were enthusiastic about it and understood the benefits, although some were concerned about reduced patient contact. Cognitive participation difficulties included engaging all staff and changing ingrained behaviours (resulting from extensive training and audit), especially for administrative and nursing staff, although staff did support each other and work together. Some patients had concerns about waiting for treatment, but most accepted sample drop-off and returning for a follow-up appointment. Reflexive monitoring revealed perceived benefits including reduced patient anxiety, seeing more patients, and boosting staff job satisfaction. Infection-specific treatment based on test results was crucial, enabling informed consultations and improving antimicrobial stewardship. Suggestions for this and other future services included: document new pathways and processes early and comprehensively disseminate to staff; involve all staff in planning, design and implementation; protect staff time for meetings and actions; consider pilots with a small group of staff/patients before sharing more widely or writing guidelines; cross-discipline training; varied methods of, sensitive and supportive communication; consider staff role impact, and ensure staffing to cover changes.

Relation to other studies

Evaluating the real-life implementation of a novel rapid results service confirms previous hypothetical/simulated studies where patients were happy with the service and willing to wait for results before treatment[15-17 26], although this depends on self-assessed infection risk[26]. Although asymptomatic patients are encouraged to use on-line postal services, some patients may wish to attend[14 41]. The benefits of treating with results and improving antimicrobial stewardship previously anticipated[13 19] are highly valued by staff and patients. We also confirm reductions in patient anxiety[13 18 19] and improved testing uptake[20-22] are likely, as well as freeing up clinician time, greater clinician confidence, and efficiencies allowing capacity to be utilised elsewhere[13].

For peer review only - http://bmjopen.bmj.com/site/about/guidelines.xhtml

The challenges of communicating with and engaging all staff, especially those 'on the ground', and the need for dedicated time for training and implementation[42] are key in healthcare quality improvement[42]. Teething issues experienced in this service – documentation of new pathways, impact on staff roles – and the challenges of changing ingrained behaviour – are common in implementation of a major service change and emphasise the importance of staff training and communication of the reason and implications for change[43].

Our findings demonstrate that successfully implementing a beneficial service change can boost staff job satisfaction and morale. Previous research has found improvements in staff satisfaction following successful sexual healthcare innovation[43]. This finding suggests the implementation realised benefits for staff - previously highlighted as influencing acceptance of change in NHS service improvement programmes[44] – and aligned with professionals values and intrinsic motivation to provide quality and effective care[42].

Implications

This study shows that a rapid NAAT-testing integrated SSHS for chlamydia and gonorrhoea can be implemented in a constrained NHS system, and is acceptable to patients, with benefits for staff, patients and public health, including reduced patient anxiety. The perceived efficiency (to be clarified in a separate quantitative evaluation) is crucial given the financial and staffing pressures on UK sexual health services[45]. Similarly, the pride of staff in their service, and enhanced staff satisfaction are important in boosting staff morale and is likely to further enhance the provision of high-quality patient care when such a service is introduced.

AMR is a major concern for gonorrhoea, and a priority worldwide[46] and in England[47]. Rapid STI services could play a vital role in reducing unnecessary antibiotic prescribing by providing test results during/soon after consultations, allowing informed clinician choices. When the technology becomes available, the addition of POCTs to detect ciprofloxacin-sensitive gonorrhoea will dramatically reduce reliance on ceftriaxone and selection pressure for AMR[48 49].

Our implementation recommendations for future services echo those from the Health Foundation, such as sensitive leadership oriented towards inclusion, agreeing roles and responsibilities at the outset and 'bringing everyone along with you'[42], as well as early documentation, piloting pathways, varying communication methods and adequate staffing. The willingness of symptomatic male patients to wait for treatment can inform development of new care pathways using POCTs[13 50], although results are limited to a single service and male patients.

Project strengths include: a multidisciplinary team including clinical academics; a strong trusting relationship between research team and clinical staff due to existing relationships and research team flexibility and responsiveness; regular feedback from researchers to clinicians using a 'trial, assess, adapt' strategy. Limitations include an all-male patient sample as the service was initially only for males, and when implemented for females, few were eligible and evaluation was hampered by the COVID-19 pandemic. We aimed to include patients with positive STI results but most (although symptomatic) were negative, limiting evaluation of follow-up appointments. COVID-19 meant fewer final batch interviews. As the rapid STI result technology develops, continued implementation evaluation is important[50], capturing, the wide-ranging impact on services, staff and patients. Evaluation for female patients is needed, given the challenges around contraception and STIs/symptoms.

For peer review only - http://bmjopen.bmj.com/site/about/guidelines.xhtml

Conclusion

As the first UK SSHS to implement rapid NAAT testing for chlamydia and gonorrhoea within an integrated service, this project faced the challenge of innovating to save time/money and improve patient experience in a constrained environment, particularly lack of funding and understaffing. Inevitable challenges – mainly related to the impact on patient pathways - were resolved and, overall, it was a success. Perceived benefits included reduced patient anxiety, seeing more patients, treating with results, reduced antibiotics use and boosting staff job satisfaction. Learning for other services considering implementing something similar include more inclusive staff engagement, sensitive communication, better documentation of changes, dealing with constant adaptations, and consideration of the impact on staff and their roles.

For peer review only

For peer review only - http://bmjopen.bmj.com/site/about/guidelines.xhtml

Acknowledgements

We would like to thank all study participants.

Funding statement

Funded by the National Institute for Health Research (NIHR) Applied Research Collaboration (ARC) West at University Hospitals Bristol and Weston NHS Foundation Trust and NIHR Health Protection Research Unit (HPRU) in Behavioural Science and Evaluation at University of Bristol in partnership with Public Health England (PHE). Award number P315. The views expressed are those of the authors and not necessarily those of the NIHR, the Department of Health and Social Care or PHE.

Competing interests statement

AL – no competing interests
EB - no competing interests
JK - no competing interests
PH - no competing interests
MC - no competing interests
MC - no competing interests
JS - no competing interests
JT - no competing interests
PM - no competing interests
JH - no competing interests

Author contributions

JH, PH, EB, JK were responsible for the study design. JH and EB were responsible for study management and coordination. EB, AL, JK and JH led data collection and analysis. All authors read, commented on and approved the final manuscript.

Data are available on request from corresponding author.

For peer review only

References

1. England PH. Sexually transmitted infections and screening for chlamydia in England, 2019. 2019
2. Public Health England. Sexually transmitted infections (STIs): annual data tables. London: Public Health England, 2018.
3. Mohammed H, Sile B, Furegato M, et al. Poor adherence to gonorrhoea treatment guidelines in general practice in England. *The British journal of general practice : the journal of the Royal College of General Practitioners* 2016;66(648):352. doi: 10.3399/bjgp16x686077
4. Eyre DW, Town K, Street T, et al. Detection in the United Kingdom of the Neisseria gonorrhoeae FC428 clone, with ceftriaxone resistance and intermediate resistance to azithromycin, October to December 2018. *Euro Surveill* 2019;24(10):1900147. doi: 10.2807/1560-7917.ES.2019.24.10.1900147
5. Sandoz KM, Rockey DD. Antibiotic resistance in Chlamydiae. *Future microbiology* 2010;5(9):1427-42. doi: 10.2217/fmb.10.96 [published Online First: 2010/09/24]
6. White C. Sexual health services on the brink. *BMJ* 2017;359 doi: 10.1136/bmj.j5395
7. Fifer H, Saunders J, Soni S, et al. 2018 UK national guideline for the management of infection with Neisseria gonorrhoeae. Updated 2020. *International journal of STD & AIDS* 2020;31(1):4-15. doi: 10.1177/0956462419886775 [published Online First: 2019/12/25]
8. Nwokolo NC, Dragovic B, Patel S, et al. 2015 UK national guideline for the management of infection with Chlamydia trachomatis. Updated 2018. *International journal of STD & AIDS* 2018;27(4):251-67. doi: 10.1177/0956462415615443 [published Online First: 2015/11/06]
9. Price MJ, Ades AE, Soldan K, et al. The natural history of Chlamydia trachomatis infection in women: a multi-parameter evidence synthesis. *Health technology assessment (Winchester, England)* 2016;20(22):1-250. doi: 10.3310/hta20220 [published Online First: 2016/03/24]
10. Harding-Esch EM, Fuller SS, Chow SC, et al. Diagnostic accuracy of a prototype rapid chlamydia and gonorrhoea recombinase polymerase amplification assay: a multicentre cross-sectional preclinical evaluation. *Clinical microbiology and infection : the official publication of the European Society of Clinical Microbiology and Infectious Diseases* 2019;25(3):380.e1-80.e7. doi: 10.1016/j.cmi.2018.06.003 [published Online First: 2018/06/16]
11. Van Der Pol B. Making the Most of Point-of-care Testing for Sexually Transmitted Diseases. *Clinical Infectious Diseases* 2019 doi: 10.1093/cid/ciz523
12. Rönn MM, Menzies NA, Gift TL, et al. Potential for Point-of-Care Tests to Reduce Chlamydia-associated Burden in the United States: A Mathematical Modeling Analysis. *Clinical Infectious Diseases* 2019 doi: 10.1093/cid/ciz519
13. Adams EJ, Ehrlich A, Turner KME, et al. Mapping patient pathways and estimating resource use for point of care versus standard testing and treatment of chlamydia and gonorrhoea in genitourinary medicine clinics in the UK. *BMJ Open* 2014;4(7) doi: 10.1136/bmjopen-2014-005322
14. Whitlock GG, Gibbons DC, Longford N, et al. Rapid testing and treatment for sexually transmitted infections improve patient care and yield public health benefits. *International journal of STD & AIDS* 2018;29(5):14. doi: 10.1177/0956462417736431 [published Online First: 2017/10/24]
15. Llewellyn CD, Sakal C, Lagarde M, et al. Testing for sexually transmitted infections among students: a discrete choice experiment of service preferences. *BMJ Open* 2013;3(10) doi: 10.1136/bmjopen-2013-003240
16. Chislett L, Clarke J. Which elements of a novel self-directed rapid asymptomatic sexually transmitted infection screening service are most important to users? . Sexually Transmitted Infections, 2015:A1–A258.
17. Mahilum-Tapay L, Laitila V, Wawrzyniak JJ, et al. New point of care Chlamydia Rapid Test--bridging the gap between diagnosis and treatment: performance evaluation study. *BMJ*

For peer review only - http://bmjopen.bmj.com/site/about/guidelines.xhtml

(Clinical research ed) 2007;335(7631):1190-94. doi: 10.1136/bmj.39402.463854.AE [published Online First: 11/30]

18. Llewellyn C, Pollard A, Miners A, et al. Understanding patient choices for attending sexually transmitted infection testing services: a qualitative study. *Sex Transm Infect* 2012;88(7):504-9. doi: 10.1136/sextrans-2011-050344 [published Online First: 2012/05/26]
19. Turner KME, Round J, Horner P, et al. An early evaluation of clinical and economic costs and benefits of implementing point of care NAAT tests for Chlamydia trachomatis and Neisseria gonorrhoea in genitourinary medicine clinics in England. *Sexually Transmitted Infections* 2013 doi: 10.1136/sextrans-2013-051147
20. Natoli L, Guy RJ, Shephard M, et al. Public health implications of molecular point-of-care testing for chlamydia and gonorrhoea in remote primary care services in Australia: a qualitative study. *BMJ Open* 2015;5(4) doi: 10.1136/bmjopen-2014-006922
21. Rompalo AM, Hsieh Y-H, Hogan T, et al. Point-of-care tests for sexually transmissible infections: what do 'end users' want? *Sexual health* 2013;10(6):541-45. doi: 10.1071/SH13047
22. Horwood J, Ingle SM, Burton D, et al. Sexual health risks, service use, and views of rapid point-of-care testing among men who have sex with men attending saunas: a cross-sectional survey. *International journal of STD & AIDS* 2016;27(4):273-80. doi: 10.1177/0956462415580504 [published Online First: 2015/04/25]
23. Lorenc T, Marrero-Guillamón I, Aggleton P, et al. Promoting the uptake of HIV testing among men who have sex with men: systematic review of effectiveness and cost-effectiveness. *Sexually Transmitted Infections* 2011;87(4):272-78. doi: 10.1136/sti.2010.048280
24. Read TRH, Hocking JS, Bradshaw CS, et al. Provision of rapid HIV tests within a health service and frequency of HIV testing among men who have sex with men: randomised controlled trial. *BMJ : British Medical Journal* 2013;347 doi: 10.1136/bmj.f5086
25. Public Health England. Strategic framework to promote the health and wellbeing of gay, bisexual and other men who have sex with men 2014a [Available from: https://www.gov.uk/government/uploads/system/uploads/attachment_data/file/313692/Strategic_Framework_to_promote_the_health_and_wellbeing_of_MSM_FINAL_DRAFT_For_comment.pdf.
26. Fuller SS, Pacho A, Broad CE, et al. "It's not a time spent issue, it's a 'what have you spent your time doing?' issue. . ." A qualitative study of UK patient opinions and expectations for implementation of Point of Care Tests for sexually transmitted infections and antimicrobial resistance. *PloS one* 2019;14(4):16. doi: 10.1371/journal.pone.0215380
27. Widdice L, Hsieh YH, Silver B, et al. Performance of the Atlas Genetics Rapid Test for Chlamydia trachomatis and women's attitudes toward point-of-care testing. *Sexually Transmitted Diseases* 2018;45(11):5. doi: 10.1097/OLQ.0000000000000865
28. Hsieh YH, Hogan MT, Barnes M, et al. Perceptions of an ideal point-of-care test for sexually transmitted infections--a qualitative study of focus group discussions with medical providers. *PloS one* 2010;5(11):e14144. doi: 10.1371/journal.pone.0014144 [published Online First: 2010/12/15]
29. Hsieh Y-H, Gaydos CA, Hogan MT, et al. What Qualities Are Most Important to Making a Point of Care Test Desirable for Clinicians and Others Offering Sexually Transmitted Infection Testing? *PloS one* 2011;6(4):e19263. doi: 10.1371/journal.pone.0019263
30. Hall C, Carter N, Crofts M, et al. P. P155 "time in clinic" survey to evaluate the potential for use of online registration. . *Sexually Transmitted Infections* 2015;91:A66-A67.
31. Huntington SE, Burns RM, Harding-Esch E, et al. Modelling-based evaluation of the costs, benefits and cost-effectiveness of multipathogen point-of-care tests for sexually transmitted infections in symptomatic genitourinary medicine clinic attendees. *BMJ Open* 2018;8
32. Turner KME, Looker KJ, Syred J, et al. Online testing for sexually transmitted infections: A whole systems approach to predicting value. *PloS one* 2019;14(2):e0212420. doi: 10.1371/journal.pone.0212420 [published Online First: 2019/02/23]

For peer review only - http://bmjopen.bmj.com/site/about/guidelines.xhtml

33. Higginbottom GM, Pillay JJ, Boadu NY. Guidance on Performing Focused Ethnographies with an Emphasis on Healthcare Research *The Qualitative Report* 2013;18(9):1-6.
34. MacGregor L, Speare N, Nicholls J, et al. The role of the evolving trends in sexual behaviours and clinic attendance patterns on increasing diagnoses of STIs: a perspective from Bristol, UK. *Sexually Transmitted Infections* 2020;Accepted for Publication
35. Malterud K, Siersma VD, Guassora AD. Sample Size in Qualitative Interview Studies:Guided by Information Power. *Qualitative Health Research* 2016;26(13):1753-60. doi: 10.1177/1049732315617444
36. Reeves S, Kuper A, Hodges BD. Qualitative research methodologies: ethnography. *BMJ* 2008;337 doi: 10.1136/bmj.a1020
37. Emerson R, Fretz R, Shaw L. Writing ethnographic fieldnotes. Chicago and London: University of Chicago Press 1995.
38. Murray E, Treweek S, Pope C, et al. Normalisation process theory: a framework for developing, evaluating and implementing complex interventions. *BMC medicine* 2010;8:63. doi: 10.1186/1741-7015-8-63 [published Online First: 2010/10/22]
39. Public Health England. Guidance on the collection and reporting of data for GUMCAD, the surveillance system for sexually transmitted infections (STIs) in England. 2019 [Available from: https://www.gov.uk/guidance/gumcad-sti-surveillance-system
40. Horner P, Blee K, O'Mahony C, et al. 2015 UK National Guideline on the management of non-gonococcal urethritis. *International journal of STD & AIDS* 2016;27(2):85-96. doi: 10.1177/0956462415586675 [published Online First: 2015/05/24]
41. Turner KME, Zienkiewicz AK, Syred J, et al. Web-Based Activity Within a Sexual Health Economy: Observational Study. *J Med Internet Res* 2018;20(3):e74. doi: 10.2196/jmir.8101
42. Dixon-Woods M, McNicol S, Martin G. Ten challenges in improving quality in healthcare: lessons from the Health Foundation's programme evaluations and relevant literature. *BMJ Quality & Safety* 2012;21(10):876-84. doi: 10.1136/bmjqs-2011-000760
43. Boog K, Heanue J, Kumar V. Implementing integrated sexual and reproductive healthcare in a large sexual health service in England: challenges and opportunities for the provider. *BMJ Sexual & Reproductive Health* 2019;45(2):164-67. doi: 10.1136/bmjsrh-2018-200090
44. Gollop R, Whitby E, Buchanan D, et al. Influencing sceptical staff to become supporters of service improvement: a qualitative study of doctors' and managers' views. *Quality and Safety in Health Care* 2004;13(2):108-14. doi: 10.1136/qshc.2003.007450
45. The Kings Fund. Understanding NHS financial pressures: How are they affecting patient care?, 2017.
46. World Health Organisation. Emergence of multi-drug-resistant Neisseria gonorrhoeae—threat of global rise in untreatable sexually transmitted infections, 2012.
47. Public Health England. PHE infectious diseases strategy 2020 to 2025, 2019.
48. Town K, Harris S, Sánchez-Busó L, et al. Genomic and Phenotypic Variability in Neisseria gonorrhoeae Antimicrobial Susceptibility, England. *Emerg Infect Dis* 2020;26(3):505-15. doi: 10.3201/eid2603.190732
49. Zienkiewicz AK, Verschueren van Rees N, Homer M, et al. Agent-based modelling study of antimicrobial-resistant Neisseria gonorrhoeae transmission in men who have sex with men: towards individualised diagnosis and treatment. *Sex Health* 2019;16(5):514-22. doi: 10.1071/sh18235 [published Online First: 2019/09/03]
50. Toskin I, Govender V, Blondeel K, et al. Call to action for health systems integration of point-of-care testing to mitigate the transmission and burden of sexually transmitted infections. *Sex Transm Infect* 2020;96(5):342-47. doi: 10.1136/sextrans-2019-054358 [published Online First: 2020/04/04]

For peer review only - http://bmjopen.bmj.com/site/about/guidelines.xhtml

Table 1: Quotes on Coherence and Cognitive Participation	
**Coherence**	**Cognitive participation**
**Staff enthusiasm and concerns**<br>It all sounded quite exciting and I was quite, not excited, but it was like, this is really good, the first one in the UK and, you know, this will be excellent, so I was quite open minded about it... lowering the use of antibiotics and fewer invasive procedures for women. (Nurse 17, T3)<br>It's an exciting opportunity, um, but also there's a bit of, um, er, you know, nerves I suppose about how it will actually be, actually run from day one, really, and how it would go (Public Health 14, T2)	**Disengaged staff**<br>It was very much the higher-up staff that kind of organised it all and they're not really the ones that are going to be doing the actual work, so I think it's really important to include clinical staff of all levels, kind of when it's getting near to it and you know, really explain, and have their opinions and thoughts on, you know, how it's gonna work (Nurse 16, T2)
**Reduced patient anxiety**<br>Results on the same day would be amazing, yeah, no doubt about that yeah, because there's always quite an – well, for me, it's like an anxious wait otherwise. Yeah, that sense of not knowing and actually "how do I manage my sex life in case anything comes back positive?" Yeah, quick results can definitely make a big difference (Mike, didn't use rapid STI service)<br>The thing that would encourage me to test more regularly, [is] if the whole thing [time spent in clinic] could be done in a shorter time slot for me (Ben, used rapid STI service)	**Barriers to engagement**<br>[Clinic managers need to] canvass people's feelings about it because I don't know that we do that very well. I think we just crack on. We don't say 'how was it for you that first week did you cope? Did you keep your head above water?' (Nurse 10, T1)
**Avoiding inappropriate antibiotics prescriptions**<br>With antibiotics I'm really – they really mess with my stomach. I really feel really sick whenever I do take them. So, I'm just pro not taking them for that reason alone. Yeah, like – I try to think about the bigger picture of the world and stuff…but I think about my own stomach more than the wider world. So, yeah, I'd – I'm always pleased to like not have to do any unnecessary drugs (Andy, results by text)<br>I just think I'd hate to kind of like take something that I didn't have and then if I ever got it again it doesn't work - so it's kind of like half society view half personal view (Harry, follow-up appointment)	**Inadequate preparation**<br>We were told on the Wednesday that it was supposed to be starting on the Monday and we were like all a bit shocked thinking 'well hang on a minute what about the training? We'll look really, really stupid in front of patients' (Admin 6, T1)<br>There was always talk about what it [Panther] could do, never talk about how it's going to function. Even at the last minute, the week before it was meant to start [Lead Consultant] came to me and goes, 'This is what I think the pathway is. Can you make notes on it?' Nobody was really clear about what happened. [Project manager] had sent a PowerPoint around and it was embedded in a way that… some staff who aren't maybe familiar with how embedded links and things work [- couldn't open it]. When the meeting came around, they said, 'Has everyone read the email about the Pathways?' half of the people went, 'What email?' …, it just wasn't presented to us in a clearest way. (Health Adviser 1, T2)

For peer review only - http://bmjopen.bmj.com/site/about/guidelines.xhtml

Table 2: Changes along the (walk-in) male patient pathway

	Before rapid STI service	Rapid STI service	
**Registering at reception**	All walk-in patients allocated an appointment in order of queue – may have to come back later that day.	Receptionist triages each patient to appropriate pathway referring to guide/pathway	
		Patients not eligible for rapid STI service are given an appointment for later that day and continue on old pathway	Rapid STI service-eligible patients wait after registering to be called up.
	Triage form used to register patients.	Triage form amended to be gender neutral and to make categories clearer.	
**Seeing a clinician/ health adviser**	At first appointment	First appointment very brief– reduced history taking. Unless uncomplicated vaginal discharge.	
	MSM and all patients needing partner notification/ risk reduction/safer sex advice see health adviser	High-risk patients (MSM or new to service) see health adviser at initial appointment.	
		Symptomatic men see doctor/trained nurse only at follow-up not drop-off, usually on same day.	
**Providing samples**	Urine and swabs: taken at time of consultation. Self-taken if asymptomatic. If symptomatic: clinician taken swab for microscopy (to detect NGU and gonorrhoea) and gonorrhoea culture; NAAT self-taken	Self-sample drop-off - Urine and swabs NAAT self-sampled in toilets, putting samples through a hatch to the lab. Instructions are on posters in the toilet and from nursing staff/NAs. Gonorrhoea culture taken by clinician on return only if NAAT positive. Swab for microscopy to detect NGU if NAAT-negative (see text above).	
	Blood samples taken by doctor/nurse/nursing assistant (NA)	Blood samples taken by doctor/nurse/NA	
**Tests results**	All STI test results in 2 -3 weeks	Chlamydia and gonorrhoea processed on Panther - results within 48 hours. Others still 2-3 weeks	
	If negative, text sent	Results by text if asymptomatic and negative.	
	If positive, HA phones patient to discuss result and arrange treatment (unless already received presumptive treatment).	Results given at follow up appointment with clinician if symptomatic or asymptomatic positive.	
**Treatment**	Treat presumptively	Wait for results before treating	
	Treat at first (only) appointment	Treat at follow-up appointment	
**Notified partners**	Treated immediately	Only treat on positive results (if sexual contact was >2 weeks; otherwise treat immediately)	

For peer review only

For peer review only - http://bmjopen.bmj.com/site/about/guidelines.xhtml

**Table 3: Collective action quotes**
**Designing and documenting new processes**<br>We need to be really clear about what we're doing when they drop-off [patients drop-off samples], what we're doing when they come back, what are we going to do about contraception, which questions are okay to leave out of the proforma. ..for consultants because we have a lot of experience and because we're used to making decisions then I think we can [unclear] a bit and we can be flexible and can you know think about the individual patient. But for nurses who work a lot to PGD's [guidelines] and like to have clear guidance. And some of the juniors as well, who will be quite new - you know they've just been changed. Because it's going to be bewildering and chaotic you know it's doesn't feel good when there's chaos on the shop floor. (Doctor 3, T1)
**Flexibility in pathways**<br>It's a case by case situation and it does help to have helpful medical staff that have been willing to make an exception. (Admin 18, T2)<br>I think the health advisers also are more able to …know the guidelines but in some situations know that you have to approach things differently, … for me personally if I was seeing someone and they kind of said 'actually I have got this dis[ease]' - you know, real clear symptoms, you know, 'and I'm really fed up with it', I'd be more inclined to say 'okay then let's get you treated…I think you can have your general thing of saying to someone 'look you know come back in the afternoon' but if you've got someone who's kind of 'actually no but I've had these symptoms for two days I've really had enough of it'. (Health Adviser 13, implementation group member, T2)
**Guidelines**<br>It's a work in progress but the problem is as the pathway evolves then the guideline will change again…because this is so rapidly moving actually, I don't think I really want to do a guideline. So it's kind of hard to have a guideline anyway but we need some kind of guidance. (Doctor 3, T1)
**Teething issues**<br>It was chaos, the first few weeks were chaos. Reception didn't know what they were doing… there was hundreds of patients around the reception, we didn't know what we were doing, so yeah, it was chaos, but it has slowly got better. (Nurse 17, T2)<br>The waiting area fills up and people are filling out the Panther triage forms on windowsills. After a while [clinic coordinator] tells receptionists on Panther desk that he had given out 16 forms. Once they get to 10 people booked for Panther returner pathway they need to go check with the lab regarding further capacity. (Observation notes, Reception area)<br>Two reception staff were unsure whether one person should be panther/same day or walk-in due to the information provided on the form. Staff consulted with person entering data on computer. They checked whether person was returning for results/treatment. They explained to the patient that a new system is in place, so they want to make sure they do the best for him. (Observation notes, Reception area)
**Understaffing**<br>It's been very stressful for staff and I think it has been an enormous amount of work for the implementation group, that I think in the private industry you'd be given huge amounts of time, whereas we virtually squeezed it in amongst everything else we've done, but that's just the NHS. (Doctor 11, T2)<br>Admin / reception team has three staff vacancies, and today there are two members of clinic staff off sick – one clinician, and one admin (clinic coordinator). The clinician would have been doing

For peer review only

For peer review only - http://bmjopen.bmj.com/site/about/guidelines.xhtml

sample drop-off, and walk-in, so have had to reduce slots for both until they get confirmation of clinical capacity from clinicians when they arrive. (Observation notes, Reception area)
**Changing ingrained behaviours**<br>It's been quite hard on staff and obviously there's a lot of – you know, if you've been doing something the same way for 10/20/maybe 30 years, that's quite a massive change for people. (Nurse 21, T3)
**Changes to clinician contact**<br>We've actually ended up seeing a lot more complicated or complex patients, at least that's how it feels. The easy patients get siphoned off quite quickly and that means that more patients [can be seen, especially the complex patients, which the nurses are less able to deal with and require a lot more consultant supervision. I think there has been a general feeling in the department that the consultant cover job is busier than it ever was before. (Doctor 11, T2)
**Challenges of changes at reception**<br>[Receptionists] were worried that they were looking like they didn't know what they were doing, because it was new and they weren't quite sure. So I think it took a, it was a lot to ask for them all really because it was a big change, but it is just that keep reminding everybody that actually, in the long term, you will get it, and it's much better for other patients once it's in place. (Admin 12, implementation group member, T2)<br>Reception staff on male desk refresh the panther decision pathway together using A4 sheet. Female staff member commented that she always has to double check the process. Reception staff discussed male staff member's confusion about eligibility for Panther. (Observation notes, Reception area)
**Concern about shorter consultations**<br>It was a huge change, because, we, it is quite a detailed consultation. We have been told time and time again that 'oh you need to ask patients about domestic violence, ask the women about female genital mutilation, you need to do this'. Then all of a sudden, they are saying, 'no, don't ask any of these things', it's like aargh! (Nurse 17, T2)<br>It does sometimes feel if I'm absolutely honest a little bit less than a level three service, you know, people are just coming in and dropping off a sample. I know that's possibly better use of our time, but it seems a little bit spurious to call it level three. (Nurse 10, T1)
**Perceived patient views on waiting for treatment**<br>I think the major anxiety that patients have is around not being treated immediately and not being treated necessarily as a contact of infection and anxiety around that. I often find that with a bit of educating that that is overcome and my major impression is that patients really appreciate it. (Doctor 11, T2)<br>I haven't had anybody who's been absolutely, you know, anti about it but there have been a couple of people who I've thought 'I'm going to treat you mate, I'm not going to wait on results' do you know what I mean? ... they're anxious, they've maybe got another partner, a regular partner, who they don't want to infect, which, you know, I can see the reasoning behind that. But I think, you know, once that kind of idea has got out amongst our regular clientele I think it will be a lot easier. (Nurse 10, T1)<br>Four young men approach the door together. [Name] lets them know that he has just 2 forms/slots left at present, and suggests that he gives these to them on the basis of who came through the door first – these two seem pleased, and head in with their forms. He asks the other two to wait here for a minute and they seem OK with this. He tells them there is a new service which means they can give people results/treatment faster, and this is why things are different.

For peer review only - http://bmjopen.bmj.com/site/about/guidelines.xhtml

He asks them to give him a yes/no answer as to whether they have any symptoms. When they say no he says that it is probably not worth them waiting as they are unlikely to be seen today, and that they could come back another morning for when the doors first open. They seem to find this acceptable. (Observation notes, front door)
**Benefit of evaluation process**<br>Staff member commented that it was helpful to have an outside voice (research team) feeding back, because sometimes when you are within the structure you can be shouting stuff and nobody hears you. (Observation notes)

**Table 4: Reflexive monitoring quotes**
**Success**<br>When you speak at the national [sexual health] meetings, people, it's a bit of a no-brainer, what we're supposed to do, and people are amazed that we've been able to introduce it [rapid STI service] cost-neutrally. Because when you look at the point-of-care systems which other people are researching, it's… more expensive, so we've adopted an innovative approach. (Doctor 19, T3)
**Quality of care**<br>The person I saw was really brilliant, like, yeah. I felt really comfortable… I really felt like I could ask anything I want and felt sort of safe (Andy, results by text1)
**Benefits**<br>It was the immediacy and the kind of reassurance that … if something was positive that you would be able to treat it straightaway. (Harry, follow-up appointment)<br>They're [patients] very happy. I mean, who wouldn't be? You find out the same day that you have got chlamydia and you can start your treatment. I mean that is brilliant. (Nurse 17, T3)<br>Dr 11: One of the advantages that we hoped would come out of introducing Panther would be that we would attract more high-risk people, because it would be seen as an attractive place to come and test, and also that it would free up staff time so that we could spend more time with risk reduction etc.<br>Interviewer: Do you think that is happening? Or is it not there yet?<br>Dr 11: I think it has started to happen, I don't think that's only down to Panther, I think that's down to some other stuff like PrEP and things like that as well. I feel like the cohort of patients that we see is increasingly complex. (Doctor 11, T2)<br>I think the most positive things are seeing your symptomatic patients with knowing what is going on with them. You know what infection they have, you know what treatment they require or if they don't have anything you can then take the time to discuss that. (Doctor 11, T2)<br>I think it's [rapid service implementation] made the staff more able to deal with change [to telephone clinics], because they had undergone experience of change with Panther pathways over the past 12-18 months…. Yes, it probably made it smoother and more efficient. (Doctor 22,T4)
**Suggested improvements**<br>The main issues that have arisen have been when the [Panther] machine fails and that can be pretty catastrophic (laughs), just because you have booked slots and patients come back and you don't, you can't even tell them whether they have chlamydia or gonorrhoea and they've, kind of, come with that expectation. (Doctor 20, T3)<br>Interviewer: If you had an imaginary clinic who were going to set out on this path, what would your advice be to them overall? With the knowledge that you've now gathered from your experience, is there a way that you could help them?

For peer review only

Dr 11: I think preparation, preparing all of the documents that support your staff on a day-to-day basis, clarifying your communication pathways, giving your lead clinicians adequate time in their work plans to do all of that. I would definitely support it, I think it's definitely been a major benefit. (Doctor 11, T2)<br>It has been a big change for all staff working, and it's difficult to know whether there was any way of realising some of the things we hadn't realised. I don't think we could have done. I think they were literally just things of implementation that have caused some additional tweaks required - and that in itself has been stressful because it's been the realisation of what are we doing in this scenario and not being quite prepared for it. (Admin 12, T2)<br>The thing that I found most interesting is the communication difficulties in amongst the staff and how difficult that has been, having an implementation group that I think represents most of the groups that it's impacted upon and the difficulty that the messages just have not got to the clinic floor, and that's an on-going issue. (Doctor 11, T2)

For peer review only

For peer review only - http://bmjopen.bmj.com/site/about/guidelines.xhtml

Elements of rapid STI service implementation	
**What**	**Who should do this**
**Changes to documentation**	
Rewrite and sign-off treatment guidelines and SOPs when new processes are agreed.	Project leads, clinical lead, project implementation/operations team and clinical governance guideline group
Change the triage process and form.	Project operations team, to include reception staff
Consider changes to IT system/medical records system.	Project leads, consultant and project implementation team
**Implementation of the actual machine and process**	
Write business case for new rapid STI service and have it signed off by PHE.	Clinic lead, clinic manager, lead consultant, project manager, in collaboration with PHE.
Source the machine, find space for it (with waste disposal) and install it. Arrange insurance (including negotiations with PHE and legal teams)	Clinic lead, operations manager, lead consultant, PHE, nursing lead
Ensure IT systems allow direct transfer of data from Panther	Project lead, clinic manager
Pilot before implementing with all patients	All staff including reception teams
Write protocol for Panther outages	Project leads, in collaboration with PHE.
Quality assessment scheme /UKAS accreditation	PHE team
**Services**	
Consider impact on other services	Project operations team
Adjust clinic timetabling to accommodate rapid STI service appointments	Operations manager
Changing medical history forms and process to accommodate the new appointment structure	Project leads and implementation team
Changes to the IT coding	Clinic data manager, project lead, clinic manager, clinical lead (minor)
**Staff engagement, training and communication**	
Put together an implementation team, to oversee implementation, and put in place mechanisms for all staff to feedback to this team	Representative from each staff group and clinic manager.
Clarify communication pathways between all staff and the implementation team	Clinic Manager, project leads
Consider the impact on staff roles and workload and if staffing changes are therefore needed	Project operations team
Regular meetings for staff involved in the new service	Possible staff to include: project leads, HAs, consultants, nursing assistants, nurses, administrative staff, researchers, IT lead, clinic manager, data manager, chlamydia screening program team lead.
Staff training and regular updates at existing staff training sessions	Led by project leads, all staff to attend
Regular departmental meetings	Project leads and clinical lead
**Patient communication**	
Communicate changes to patients – write leaflets/posters/website	Project leads

For peer review only

COREQ (COnsolidated criteria for REporting Qualitative research) Checklist

A checklist of items that should be included in reports of qualitative research. You must report the page number in your manuscript where you consider each of the items listed in this checklist. If you have not included this information, either revise your manuscript accordingly before submitting or note N/A.

Topic	Item No.	Guide Questions/Description	Reported on Page No.
**Domain 1: Research team and reflexivity**			
Personal characteristics			
Interviewer/facilitator	1	Which author/s conducted the interview or focus group?	
Credentials	2	What were the researcher's credentials? E.g. PhD, MD	
Occupation	3	What was their occupation at the time of the study?	
Gender	4	Was the researcher male or female?	
Experience and training	5	What experience or training did the researcher have?	
Relationship with participants			
Relationship established	6	Was a relationship established prior to study commencement?	
Participant knowledge of the interviewer	7	What did the participants know about the researcher? e.g. personal goals, reasons for doing the research	
Interviewer characteristics	8	What characteristics were reported about the inter viewer/facilitator? e.g. Bias, assumptions, reasons and interests in the research topic	
**Domain 2: Study design**			
Theoretical framework			
Methodological orientation and Theory	9	What methodological orientation was stated to underpin the study? e.g. grounded theory, discourse analysis, ethnography, phenomenology, content analysis	
Participant selection			
Sampling	10	How were participants selected? e.g. purposive, convenience, consecutive, snowball	
Method of approach	11	How were participants approached? e.g. face-to-face, telephone, mail, email	
Sample size	12	How many participants were in the study?	
Non-participation	13	How many people refused to participate or dropped out? Reasons?	
Setting			
Setting of data collection	14	Where was the data collected? e.g. home, clinic, workplace	
Presence of non-participants	15	Was anyone else present besides the participants and researchers?	
Description of sample	16	What are the important characteristics of the sample? e.g. demographic data, date	
Data collection			
Interview guide	17	Were questions, prompts, guides provided by the authors? Was it pilot tested?	
Repeat interviews	18	Were repeat inter views carried out? If yes, how many?	
Audio/visual recording	19	Did the research use audio or visual recording to collect the data?	
Field notes	20	Were field notes made during and/or after the inter view or focus group?	
Duration	21	What was the duration of the inter views or focus group?	
Data saturation	22	Was data saturation discussed?	
Transcripts returned	23	Were transcripts returned to participants for comment and/or	

For peer review only

Topic	Item No.	Guide Questions/Description	Reported on Page No.
		correction?	
**Domain 3: analysis and findings**			
Data analysis			
Number of data coders	24	How many data coders coded the data?	
Description of the coding tree	25	Did authors provide a description of the coding tree?	
Derivation of themes	26	Were themes identified in advance or derived from the data?	
Software	27	What software, if applicable, was used to manage the data?	
Participant checking	28	Did participants provide feedback on the findings?	
Reporting			
Quotations presented	29	Were participant quotations presented to illustrate the themes/findings? Was each quotation identified? e.g. participant number	
Data and findings consistent	30	Was there consistency between the data presented and the findings?	
Clarity of major themes	31	Were major themes clearly presented in the findings?	
Clarity of minor themes	32	Is there a description of diverse cases or discussion of minor themes?	

Developed from: Tong A, Sainsbury P, Craig J. Consolidated criteria for reporting qualitative research (COREQ): a 32-item checklist for interviews and focus groups. *International Journal for Quality in Health Care*. 2007. Volume 19, Number 6: pp. 349 – 357

Once you have completed this checklist, please save a copy and upload it as part of your submission. DO NOT include this checklist as part of the main manuscript document. It must be uploaded as a separate file.

For peer review only

BMJ Open

What can be learnt from a qualitative evaluation of implementing a rapid sexual health testing, diagnosis and treatment service?

Journal:	*BMJ Open*
Manuscript ID	bmjopen-2021-050109.R1
Article Type:	Original research
Date Submitted by the Author:	11-Aug-2021
Complete List of Authors:	Lorenc, Ava; University of Bristol, Population Health Sciences, Bristol Medical School; National Institute for Health Research, Applied Research Collaboration West<br>Brangan, Emer; University of the West of England, Department of Nursing and Midwifery<br>Kesten, Joanna; University of Bristol, National Institute for Health Research, Applied Research Collaboration West (NIHR ARC West); University of Bristol, NIHR Health Protection Research Unit in Behavioural Science and Evaluation, Population Health Sciences, Bristol Medical School<br>Horner, Paddy; University of Bristol, NIHR Health Protection Research Unit in Behavioural Science and Evaluation, Population Health Sciences, Bristol Medical School; University Hospitals Bristol and Weston NHS Foundation Trust, Unity Sexual Health<br>Clarke, Michael; University Hospitals Bristol and Weston NHS Foundation Trust, Unity Sexual Health<br>Crofts, Megan; University Hospitals Bristol and Weston NHS Foundation Trust, Unity Sexual Health<br>Steer, Jonathan; Public Health England, South West Regional Laboratory, National Infection Service<br>Turner, Jonathan; Public Health England, South West Regional Laboratory, National Infection Service<br>Muir, Peter; Public Health England, South West Regional Laboratory, National Infection Service<br>Horwood, Jeremy; University of Bristol, National Institute for Health Research, Applied Research Collaboration West (NIHR ARC West), University Hospitals Bristol and Weston NHS Foundation Trust; University of Bristol, NIHR Health Protection Research Unit in Behavioural Science and Evaluation, Population Health Sciences
**Primary Subject Heading**:	Sexual health
Secondary Subject Heading:	Health services research, Qualitative research
Keywords:	QUALITATIVE RESEARCH, SEXUAL MEDICINE, Organisational development < HEALTH SERVICES ADMINISTRATION & MANAGEMENT, Change management < HEALTH SERVICES ADMINISTRATION & MANAGEMENT, COVID-19

SCHOLARONE™
Manuscripts

I, the Submitting Author has the right to grant and does grant on behalf of all authors of the Work (as defined in the below author licence), an exclusive licence and/or a non-exclusive licence for contributions from authors who are: i) UK Crown employees; ii) where BMJ has agreed a CC-BY licence shall apply, and/or iii) in accordance with the terms applicable for US Federal Government officers or employees acting as part of their official duties; on a worldwide, perpetual, irrevocable, royalty-free basis to BMJ Publishing Group Ltd ("BMJ") its licensees and where the relevant Journal is co-owned by BMJ to the co-owners of the Journal, to publish the Work in this journal and any other BMJ products and to exploit all rights, as set out in our licence.

The Submitting Author accepts and understands that any supply made under these terms is made by BMJ to the Submitting Author unless you are acting as an employee on behalf of your employer or a postgraduate student of an affiliated institution which is paying any applicable article publishing charge ("APC") for Open Access articles. Where the Submitting Author wishes to make the Work available on an Open Access basis (and intends to pay the relevant APC), the terms of reuse of such Open Access shall be governed by a Creative Commons licence – details of these licences and which Creative Commons licence will apply to this Work are set out in our licence referred to above.

Other than as permitted in any relevant BMJ Author's Self Archiving Policies, I confirm this Work has not been accepted for publication elsewhere, is not being considered for publication elsewhere and does not duplicate material already published. I confirm all authors consent to publication of this Work and authorise the granting of this licence.

For peer review only

What can be learnt from a qualitative evaluation of implementing a rapid sexual health testing, diagnosis and treatment service?

Authors

Ava Lorenc[1,2], Emer Brangan[1,3], Joanna M Kesten[1,2,4], Paddy Horner[1,4,5], Michael Clarke[5], Megan Crofts[5], Jonathan Steer[6], Jonathan Turner[6], Peter Muir[6], Jeremy Horwood[1,2,4]

[1.] Population Health Sciences, Bristol Medical School, University of Bristol, Canynge Hall, 39 Whatley Road, Bristol, BS8 2PS

[2.] National Institute for Health Research, Applied Research Collaboration West (NIHR ARC West), University Hospitals Bristol and Weston NHS Foundation Trust, 9th Floor, Whitefriars, Lewins Mead, Bristol, BS1 2NT

[3] University of the West of England
Department of Nursing and Midwifery, Faculty of Health and Applied Sciences, University of the West of England, Frenchay Campus, Coldharbour Lane, Bristol, BS16 1QY

[4] NIHR Health Protection Research Unit in Behavioural Science and Evaluation, Population Health Sciences, Bristol Medical School, University of Bristol, Canynge Hall, 39 Whatley Road, Bristol, BS8 2PS

[5] Unity Sexual Health, University Hospitals Bristol and Weston NHS Foundation Trust, Tower Hill, Avon, Bristol BS2 0JD

[6] Public Health England South West Regional Laboratory, National Infection Service, Pathology Sciences Building, Science Quarter, Southmead Hospital, Bristol BS10 5NB

Corresponding author: Jeremy Horwood, J.Horwood@bristol.ac.uk

Word count: 4640

For peer review only

Abstract

Objectives

To investigate experiences of implementing a new rapid sexual health testing, diagnosis and treatment service.

Design

A theory-based qualitative evaluation with a focused ethnographic approach using non-participant observations and interviews with patient and clinic staff. Normalisation Process Theory was used to structure interview questions and thematic analysis.

Setting

A sexual health centre in Bristol, UK.

Participants

26 patients and 21 staff involved in the rapid sexually transmitted infection (STI) service were interviewed. Purposive sampling aimed for a range of views and experiences and socio-demographics and STI results for patients, job grades and roles for staff. 40 hours of observations conducted.

Results

Implementation of the new service required co-ordinated changes in practice across multiple staff teams. Patients also needed to make changes to how they accessed the service. Multiple small ‘pilots’ of process changes were necessary to find workable options. For example, the service was introduced in phases beginning with male patients. This responsive operating mode created challenges for delivering comprehensive training and communication in advance to all staff. However, staff worked together to adjust and improve the new service, and morale was buoyed through observing positive impacts on patient care. Patients valued faster results and avoiding unnecessary treatment. Patients reported that they were willing to drop-off self-samples and return for a follow-up appointment, enabling infection-specific treatment in accordance with test results thus improving antimicrobial stewardship.

Conclusions

The new service was acceptable to staff and patients. Implementation of service changes to improve access and delivery of care in the context of stretched resources can pose challenges for staff at all levels. Early evaluation of pilots of process changes, played an important role in the success of the service by rapidly feeding back issues for adjustment. Visibility to staff of positive impacts on patient care is important in maintaining morale.

Strengths and limitations of this study

- The ‘trial, assess, adapt’ strategy (reflexive process of observation, feedback, and resulting action) meant that evaluation and implementation occurred in parallel and allowed researchers to capture the active process.
- The evaluation benefitted the staff, as researchers provided ongoing feedback and suggestions for service improvements and provided a space for reflection.

For peer review only - http://bmjopen.bmj.com/site/about/guidelines.xhtml

For peer review only

- A strong and trusting relationship between research and clinic staff arose from researcher flexibility and timely responsiveness and allowed good researcher access to spaces, staff and meetings.
- Frequent, regular and extensive physical presence of the researcher in various clinic settings was crucial as much of the process was not documented.
- The patient sample was limited due to recruitment being cut short by the COVID-19 pandemic lockdown, and we only interviewed males due to the pathway being initially implemented for male patients during the evaluation period.

For peer review only

For peer review only - http://bmjopen.bmj.com/site/about/guidelines.xhtml

Introduction

Rates of sexually transmitted infections (STIs) continue to increase in England despite control efforts, with a 5% increase between 2018-2019[1]. *Chlamydia trachomatis* (chlamydia) and *Neisseria gonorrhoeae* (gonorrhoea) are the most common, with 226,411, and 70,982 diagnoses reported in England in 2019, a 5% and 26% increase since 2018[2]. The rise in gonorrhoea is particularly concerning as first line treatment effectiveness is threatened by the development of antimicrobial resistance (AMR)[3,4]. Most STIs are diagnosed through Specialist Sexual Health Services (SSHS), the provision of which is increasingly challenging as funding (via government public health grant), has been steadily cut since 2015[5].

Chlamydia and gonorrhoea if left untreated may cause pelvic inflammatory disease (PID) in women, which can result in infertility, ectopic pregnancy, and chronic pelvic pain[6-8]. Infections are often asymptomatic, particularly in women, and when they do cause symptoms and/or signs these are not pathognomonic[6 7]. Nucleic acid amplification tests (NAATs) provide accurate detection. Early detection and treatment helps prevent the spread of STIs and the development of complications. Point-of-care testing (POCT; results within 15-30mins)[9] and rapid STI services (results on the same day) can potentially improve care and reduce costs, due to reduced time from diagnosis to treatment and number lost to follow up. This can increase testing uptake, improve partner notification rates and enable better and timelier clinician decisions, improving outcomes such as fewer unnecessary treatments and reduced PID risk[10-13]. Patients prefer rapid STI testing[14-16] and are happy to wait at clinic for results. Rapid testing can reduce anxiety[17 18] and improve patient acceptability of services and uptake of testing[19-22]. HIV POCT is well established and preferred by high risk men who have sex with men (MSM)[23 24]. Although studies suggest a limit of 30 minutes to wait for results[25-28], experience from our service indicates patients would be prepared to wait longer than 20 minutes for their result[29].

However, much of the evidence is from modelling and hypothetical views of clinicians and/or patients[10-12 25-28 30], with little real-life implementation evaluation[31], and rarely considering the complexity of patient visits including both asymptomatic and symptomatic patients with multiple needs e.g. female contraception. There is an urgent need to evaluate staff and patient preferences, and clinical benefits and cost effectiveness in practice.

In November 2018, a UK SSHS implemented a first-of-its-kind rapid STI testing, diagnosis and treatment service, using a clinic-based Hologic 'Panther' NAAT diagnostic machine. In 2017, the clinic introduced an online STI and HIV testing postal service for asymptomatic patients[32]. The new rapid service provides chlamydia and gonorrhoea results in 3.5 hours (previously over a week when tested in the microbiology laboratory), to improve patient care while reducing costs (see figure 1 for an overview of service redesign). This evaluation assessed the best service model and patient and staff acceptability, to refine and improve the service and support implementation in other SSHSs. We report the qualitative evaluation of male patient and staff views and experiences of the implementation of the first phase of this new rapid STI service.

For peer review only - http://bmjopen.bmj.com/site/about/guidelines.xhtml

Methods

Design

The evaluation was ethnographic, theory-based (informed by Normalisation Process Theory (NPT) a sociological theory that has been widely promoted as a means to understand implementation, embedding and integration of innovation in healthcare settings [33]) and used observations and interviews[34]. This approach provided insights into peoples' views and actions via the contexts and locations they inhabited[35] and supported real-time feedback to refine and improve the service. The study focussed on four timepoints selected pragmatically during 16 months of evaluation: T1 at start of implementation; T2 after 6 months; T3 after 14 months; T4 at 16 months during the COVID-19 pandemic lockdown.

Setting

A sexual health clinic in Bristol (population 450,000), UK.

Participants

Due to the new services being initially introduced for the male pathway only, male patients (over 16 years old) and staff at the sexual health clinic were interviewed. Patients were invited to take part, via a clinic survey about PrEP (pre-exposure prophylaxis for HIV) [36] and physically attending the clinic at T1, T2 and T3. Cross sectional interviewed were conducted with staff at four timepoints at T1, T2, T3, and T4. One staff member was interviewed twice. Purposive sampling[34] attempted to capture maximum variation in views and experiences, and socio-demographics and STI test results for patients, and job grades and roles for staff (administrative staff, consultants, doctors, nurses/nursing assistants, health advisers, Public Health England (PHE)); responsible for the Panther lab and administration. Information sheets were provided to male patients by staff at the clinic or via email from researchers, with patients asked to contact the researcher and ask questions before deciding to take part. Staff were emailed by the researcher about the study.

Data collection

Following the concept of information power, data collection continued until sufficient data to meet the study objectives had been collected with continuous, pragmatic assessment of information within our sample [37]. Issues informing information power include the study aim (i.e. broader aims require a larger sample), the sample (i.e. a smaller sample is needed if participants have rich experiences relevant to the research), use of theory (studies supported by theory require smaller sample sizes), depth and quality of the data (i.e. smaller samples are needed with focused and clear data) and the analysis type (larger samples are needed for exploratory analysis) [37].

In the first 6 months of service implementation, observations were conducted by EB and JK at varying times/days, in reception, laboratory and waiting areas. Non-participant observations focussed on day-to-day operations, how clinic staff integrated the new service and any factors which promoted or inhibited successful incorporation [35]. Written accounts based on brief field notes taken at the time included observations, conversations with staff, and reflection on what has been observed[38]. Observations recorded activities, events, their time and location and described interactions, communication patterns, workflows and tasks in the Unity clinic environment.

For peer review only - http://bmjopen.bmj.com/site/about/guidelines.xhtml

Interview topic guides informed by NPT explored: views and experiences of the service; impact on workload and clinical practice; information and support needs, sustainability and future implementation of the service. Patient interviews took place throughout the evaluation period and explored their experience and views of the service including acceptability, barriers and facilitators to uptake. Patients were offered a £10 High Street shopping voucher. Interviews were conducted by experienced qualitative senior research associates AL/JMK/EB, used flexible topic guides and open-ended questioning, were face-to-face (at the clinic or University) or by telephone, and lasted around 30 minutes. Participants were told that the study was evaluating the rapid results service and that interviewers were independent of the service.

Analysis

Interviews were audio recorded, transcribed verbatim and imported into QSR NVivo (version 10) with transcribed observation fieldnotes. Ongoing and iterative analysis informed further data collection through changes to the topic guide and feedback to healthcare staff to aid the adaptation and refinement of the rapid service. 'Codebook' thematic, inductive analysis by EB/AL identified and analysed patterns and themes salient to participants and observations [39]. Initial noting of ideas was followed by line-by-line examination and inductive coding. A subset of transcripts and observations were independently double-coded by EB/JH and discrepancies discussed to contribute to the generation and refinement of codes to maximise rigour. Themes were discussed by the multi-disciplinary research team to ensure credibility and confirmability. Negative cases and reasons for deviance were explored. The four NPT constructs[33] were used to further develop themes deductively. NPT proposes that successful implementation of an intervention is dependent on participants ability to fulfil four criteria : 1) Coherence - (sense-making - understanding and opinion of the intervention purpose); 2) Cognitive Participation (commitment and engagement with the intervention); 3) Collective Action (the work that individuals and organisations have to do to make the intervention function); 4) Reflexive Monitoring (appraisal of the intervention once it is in use).

Ethical approval

South West Frenchay Research Ethics Committee granted approval, reference 18/SW/0090.

Patient and Public Involvement

PPI meetings with three people who recently used the clinic informed the study design. These meetings reviewed patient-facing materials and discussed the acceptability of proposed recruitment and data collection.

Results

Participants/hours of observation

25 observations were conducted, approximately 40 hours total, 25 staff interviews (24 participants), 26 patient interviews. Patients ranged in age from 19 to 57 years, average 34 years, index of multiple deprivation scores ranged from 2 to 10, average 5.4, and most identified as MSM. Two had positive STI test results.

For peer review only - http://bmjopen.bmj.com/site/about/guidelines.xhtml

Coherence (sense-making)

Staff and patients welcomed rapid testing (Table 1). All staff saw it as beneficial and many were excited about doing something new, particularly to improve service access which was limited by a lack of pre-bookable appointments, high observed demand (manifesting in long queues outside before the clinic opened each morning to access limited capacity walk-in appointments) and staff shortages. Some staff had concerns around anticipated reduced clinician contact and shorter consultations in the new service. Patients most valued a potentially quicker and more convenient service, but also reduced anxiety from waiting for results, which may increase testing frequency. Staff also welcomed being able to provide treatment based on results and avoiding unnecessary antibiotic prescribing (previously treatment was prescribed presumptively for symptomatic patients due to the week-long wait for test results). Some patients valued avoiding unnecessary antibiotic treatment on a personal basis and acknowledged wider societal issues.

Cognitive participation (buy in)

The importance of engaging the whole clinic team in the service redesign was recognised but challenging with a large team and many part-time staff (Table 1). Formal engagement was via an implementation team, project meetings and staff training sessions. Engagement of 'on the ground' staff was inadequate, with administrative and nursing staff feeling particularly disengaged and having limited preparation time, citing little communication (due to busy work schedules with limited time for accessing emails), training (lack of access as many staff were part-time and did not work on the day training was delivered) or involvement in project meetings or the implementation group (which was initially only senior staff, although this did improve).

Engagement was also limited by a lack of protected project time – implementation work was fitted around existing high workloads, rapid changes made timely feedback difficult, and burn out from staff pressures (funding cuts, understaffing, and high service demand).

Collective action (putting rapid STI test results service into operation)

The service was implemented for males in November 2018 and for all in August 2019. The changes to the patient pathway and quotes relating to collective action are listed in Table 2 and 3 respectively. Some of these caused challenges:

- After registering, eligible patients wait at clinic to be seen within the first hour of session, rather than coming back hours or days later to a "slot" on the walk-in clinic.

- The shorter initial appointments, with reduced medical record completion and fewer physical examinations, was a 'huge change' and source of concern and anxiety for clinicians both before and during the changes, due to perceived loss of opportunities for patient discussions e.g. about domestic violence, female genital mutilation, alcohol use, and contraception, seen as essential for a 'holistic', 'integrated' 'level 3 service'. This did improve with practice, and patients with particularly concerning issues were referred for a health adviser consultation, which was longer under the new service.

- Self-sampling drop-off meant reduced clinical contact, particularly for asymptomatic, low-risk men with negative test results (health advisers only see high-risk/new MSM patients at the first visit). The walk-in clinic was therefore more demanding, as the case mix changed, seeing more symptomatic patients and with complex presentations. Although reduced clinical contact with asymptomatic patients was a planned cost-saving benefit, nursing assistants (running the sample

For peer review only - http://bmjopen.bmj.com/site/about/guidelines.xhtml

drop-off sessions) often ended up collecting mandatory data (GUMCAD surveillance system[40]) and answering patient clinical queries, which they were not qualified/paid/willing to do.

- Chlamydia and gonorrhoea treatments were to be given based on results, not presumptively unless sexual contact with a case was within the 2 weeks window period and patient requested treatment[6 7]
    - Men with symptoms of urethritis were first tested for chlamydia/gonorrhoea and booked to return more than 4 hours later. If NAAT-positive they were treated according to British Association for Sexual Health and HIV (BASHH) chlamydia and gonorrhoea guidelines[6 7] and if negative tested for urethritis and managed according to BASHH guidelines[41] [with reassurance, including a leaflet, if negative] and told to re-attend for an early morning smear if their symptoms did not resolve. Some patients, particularly regulars, were not keen on this longer wait for treatment, although this did improve.
    - A minority of clinicians deviated from protocol and treated presumptively, especially for patients who were particularly anxious. Staff reported mixed patient understanding of only treating when results were available, with detailed explanations needed, but patients were amenable once they understood.

Important in collective action was designing and documenting the new patient pathways, which needed to be clear but flexible, responding to individual patient situations and need e.g. anxiety, medical history, relationship status, availability to attend clinic, with some staff deviation from protocol. Guidelines, SOPs (Standard Operating Procedures) and pathways had to be rewritten. However, detailed SOPs were not always in place prior to implementation of a new modification to a pathway, making it difficult for staff to keep up with current processes. This was due to the repeated and frequent changes to clinic processes/patient pathways, and the lack of protected administrative staff time which meant that when patient pathways were revised following staff feedback these could take over 6 weeks to review and be signed off by the clinical governance group. The triage form for patients to self-identify at reception whether symptomatic, their risk level, and if they had had sex against their will was revised three times during implementation to make it clearer which pathway should be followed. This was stressful for reception staff, particularly given their limited engagement and training. Observation showed that they annotated a copy of the triage form to remind them of the pathways for different responses.

Patients were happy with communication about the changes made (via the website, staff, consultations, on the triage form clipboard).

The implementation process was one of continual adaptations. Although staff accepted this as inevitable due to the novelty of the service, it was difficult. During initial implementation, 'teething issues' were experienced, including administrative staff not knowing which patients were eligible for the service, dealing with the high volume of patients when the doors first open, and the best way to triage patients.

The responsive model meant comprehensive preparatory training and communication to all staff was challenging, although communication challenges were not unique to this project. Multiple methods of communication were essential. The evaluation process aided communication, and researchers were able to suggest solutions to problems based on the non-participant observation. For example, researcher (EB) co-developed with the clinical team a laminated card for patients explaining the new service in response to the researcher observation that patients were given variable information by reception staff. However, staff meetings were often poorly attended as

For peer review only - http://bmjopen.bmj.com/site/about/guidelines.xhtml

For peer review only

many staff were part-time and these were held at a fixed time each week. Lack of training was particularly noticeable and stressful for administrative staff, much of which was ‘on the job’.

Understaffing (a chronic problem in the NHS) and extra workload (due to both the new system and increased demand), affected implementation, caused much stress, and aggravated the teething problems. This applied to all teams, but particularly reception staff. There were ongoing budget cuts and lack of funding during the implementation period.

Many staff found changing ingrained behaviours difficult, particularly reducing the content and duration of consultations when they had been taught to maximise patient contact.

Staff worked together to adjust and improve the new service, identifying problems and opportunities and innovating in their own practice, overseen and supported by the implementation groups, and morale was buoyed by the positive impact on patient care and the positive feedback from the research team.

Reflexive monitoring (appraisal of STI test results service into operation)

Contextual factors

Contextual factors influencing the service experience included: inadequate service funding; understaffing; ongoing communication problems; increased use of postal testing (less complex patients use postal testing so more complex at walk-in); increasing use of PrEP; increasing service demand; and increasing societal awareness of gender issues. Issuing triage forms to male patients on arrival created tensions around sensitively managing patients who did not identify with their sex assigned at birth (including trans and non-binary patients), this process was amended following feedback from the research team.

Success

Overall, the new service was seen as successful as it was implemented and running fairly smoothly after initial teething problems (Table 4). Although the process was challenging, implementation was an achievement, given the constraints on resources and staffing and lack of additional funding. Staff were credited with being adaptable, highly motivated, hardworking and mutually supportive. They were proud of being part of something new. The evaluation process played an important role in the success of the service by rapidly feeding back issues for resolution.

Patients rated the quality of the new service highly, with some patients specifically requesting it. There was interest/enthusiasm from other UK SSHS.

Although staff were initially concerned that the changes would jeopardise the high quality of care, this does not appear to have been realised, with patients very positive about staff and the ability to raise concerns and discuss issues.

Benefits

Decreased time to diagnosis and treatment meant less patient anxiety while waiting for results. Self-testing and less physical examinations involving invasive sampling (urethral swab) was generally preferred by patients. Staff perceived that the service was able to see more patients, and that clinicians and health advisers could spend more time and better engage with complex and higher risk patients due to more efficient processing of patients with straightforward needs. Staff, and some patients, were pleased to be able to treat with results, which promoted informed discussions

For peer review only - http://bmjopen.bmj.com/site/about/guidelines.xhtml

and reduced antibiotic use, secondary complications, and onward transmission. Most patients were happy to wait up to 48 hours for treatment.

Staff satisfaction was boosted from doing something new and exciting and achieving implementation, contributing to enhanced teamwork and coherence. Staff reported improvements in their work experience and job satisfaction, mainly the improvements to consultations with patients, including consultants seeing more complex patients. These boosts to staff satisfaction and morale, gave the team confidence that they could make further service improvements, demonstrated by the changes made during the COVID-19 pandemic during which staff reported being more 'change-ready'.

Suggested improvements

For many staff the most important implementation improvements were preparation of documentation and engaging and communicating (especially face-to-face) with all staff but particularly nursing and reception. It was also recommended that, if possible, staff needed to be better prepared for behavioural change and multiple continual adaptations, and given protected time for the project, and the impact on staff roles and workloads better considered. Other areas for improvement were: consistency in the rapidity of results and contingency planning for malfunctions (sometimes results were not available on time due to Panther breakdowns); more and earlier information for patients, especially on the process and timings (waiting times, results notification etc).

Based on our data, suggested improvements to the service changes are:

- Document new processes and pathways as soon as possible.
- Engage and involve staff at all levels and with direct experience in each relevant area of work, as well as management and leadership roles, to improve process design iterations.
- Small-scale pilots of the new service with patients, to test and refine draft processes to reduce staff stress and confusion.
- Bring teams together for training to facilitate information exchange and understanding.
- Supportive communication from senior staff, and a variety of methods for communication including written, training sessions, on-the-job support, informal, and nominated individuals for support.
- Consider the impact of the changes on staff roles and workload.
- Consider wider use of phone/video clinics, which were implemented during physical distancing requirements of COVID-19, but may have benefits elsewhere.

The supplementary file summarises service considerations for implementing a rapid STI service, and relevant teams/job roles.

Discussion

Principal findings

The first UK rapid NAAT testing integrated SSHS for chlamydia and gonorrhoea was successfully implemented despite funding and staff shortages. Inevitable initial challenges were resolved and, overall, it was well received. Staff were enthusiastic about it and understood the benefits, although some were concerned about reduced patient contact. The use of NPT allowed for examination of issues with both the design of the rapid service and its implementation. Cognitive participation difficulties included engaging all staff and changing ingrained behaviours (resulting from extensive

For peer review only - http://bmjopen.bmj.com/site/about/guidelines.xhtml

training and audit), especially for administrative and nursing staff, although staff did support each other and work together. Some patients had concerns about waiting for treatment, but most accepted sample drop-off and returning for a follow-up appointment. Reflexive monitoring revealed perceived benefits including reduced patient anxiety, seeing more patients, and boosting staff job satisfaction. Infection-specific treatment based on test results was crucial, enabling informed consultations and improving antimicrobial stewardship. Suggestions for this and other future services included: document new pathways and processes early and comprehensively disseminate to staff; involve all staff in planning, design and implementation; protect staff time for meetings and actions; consider pilots with a small group of staff/patients before sharing more widely or writing guidelines; cross-discipline training; varied methods of, sensitive and supportive communication; consider staff role impact, and ensure staffing to cover changes.

Relation to other studies

Evaluating the real-life implementation of a novel rapid results service confirms previous hypothetical/simulated studies where patients were happy with the service and willing to wait for results before treatment[14-16 25]. Whereas previous research has found that the patients found the hypothetical scenario of waiting up to 40 minutes for test results acceptable[25 26], our findings demonstrate that patients were happy to wait up to 48 hours for treatment based on results. Willingness to wait has been found to be dependent on self-assessed infection risk and anxiety about their infection status [25]. Our findings demonstrate that the rapid service can lead to less patient anxiety due to shorter time waiting for results and therefore should target patients concerned they are infected. Although asymptomatic patients are encouraged to use on-line postal services, some patients may wish to attend in-person clinics[13 42]. The benefits of treating with results and improving antimicrobial stewardship previously anticipated[12 18] are highly valued by staff and patients in our evaluation. Modelling studies have demonstrated that rapid testing can enable faster treatment, reduces infectious periods, and leads to fewer transmissions, partner attendances and clinic costs[43 44]. Rapid diagnostics and treatment can in increase the proportion of individuals receiving timely treatment and decrease community prevalence of STIs[45 46] and recently has been seen as a key factor contributing to in the reducing new HIV infections in London and ensuring those with HV receive fast and optimal care[47]. Our findings also confirm reductions in patient anxiety[12 17 18] and improved testing uptake[19-21] are likely, as well as freeing up clinician time, greater clinician confidence, and efficiencies allowing capacity to be utilised elsewhere[12].

The challenges of communicating with and engaging all staff, especially those ‘on the ground’, and the need for dedicated time for training and implementation[48] are key in healthcare quality improvement[48]. Teething issues experienced in this service – documentation of new pathways, impact on staff roles – and the challenges of changing ingrained behaviour – are common in implementation of a major service change and emphasise the importance of staff training and communication of the reason and implications for change[49].

Our findings demonstrate that successfully implementing a beneficial service change can boost staff job satisfaction and morale. Previous research has found improvements in staff satisfaction following successful sexual healthcare innovation[49]. This finding suggests the implementation realised benefits for staff - previously highlighted as influencing acceptance of change in NHS service improvement programmes[50] – and aligned with professionals values and intrinsic motivation to provide quality and effective care[48].

Implications

For peer review only - http://bmjopen.bmj.com/site/about/guidelines.xhtml

This study shows that a rapid NAAT-testing integrated SSHS for chlamydia and gonorrhoea can be implemented in a constrained NHS system, and is acceptable to patients, with benefits for staff, patients and public health, including reduced patient anxiety. The perceived efficiency (to be clarified in a separate quantitative evaluation) is crucial given the financial and staffing pressures on UK sexual health services[51]. Similarly, the pride of staff in their service, and enhanced staff satisfaction are important in boosting staff morale and is likely to further enhance the provision of high-quality patient care when such a service is introduced.

AMR is a major concern for gonorrhoea, and a priority worldwide[52] and in England[53]. Rapid STI services could play a vital role in reducing unnecessary antibiotic prescribing by providing test results during/soon after consultations, allowing informed clinician choices. When the technology becomes available, the addition of POCTs to detect ciprofloxacin-sensitive gonorrhoea will dramatically reduce reliance on ceftriaxone and selection pressure for AMR[54 55].

Our implementation recommendations for future services echo those from the Health Foundation, such as sensitive leadership oriented towards inclusion, agreeing roles and responsibilities at the outset and 'bringing everyone along with you'[48], as well as early documentation, piloting pathways, varying communication methods and adequate staffing. The willingness of symptomatic male patients to wait for treatment can inform development of new care pathways using POCTs[12 56], although results are limited to a single service and male patients.

Strengths and limitations

Project strengths include: integration of findings from multiple qualitative methods generating rich insights, a multidisciplinary team including clinical academics; a strong trusting relationship between research team and clinical staff due to existing relationships and research team flexibility and responsiveness; regular feedback from researchers to clinicians using a 'trial, assess, adapt' strategy. EB and JK came to the observations as experienced researchers and with good knowledge of the plans for the service changes and reasons for them. The researchers were surprised at how quickly it was possible to provide information and feedback to the implementation team which they clearly valued highly and rapidly implemented changes based on it. The researchers could move freely between different physical areas of the clinic and stages of the process in a way which clinic staff were not free to do, which provided early insights. Due to the study design and relationships, these insights could be discussed promptly with relevant staff - and so sense checked, and action taken in response if appropriate (changes to clinic processes; further data collection etc.). The rapid, supportive, evidence-based feedback which the researchers could provide seemed to quickly build the confidence of the key implementation staff in the research process. The researchers appeared to be quickly accepted as trusted team members, with the capacity to help with the work at hand (rather than creating 'research burden').

Limitations include an all-male patient sample as the service was initially only for males, and when implemented for females, few were eligible and evaluation was hampered by the COVID-19 pandemic. We aimed to include patients with positive STI results but most (although symptomatic) were negative, limiting evaluation of follow-up appointments. COVID-19 meant fewer final batch interviews. As the rapid STI result technology develops, continued implementation evaluation is important[56], capturing, the wide-ranging impact on services, staff and patients. Evaluation for female patients is needed, given the challenges around contraception and STIs/symptoms.

For peer review only - http://bmjopen.bmj.com/site/about/guidelines.xhtml

Conclusion

As the first UK SSHS to implement rapid NAAT testing for chlamydia and gonorrhoea within an integrated service, this project faced the challenge of innovating to save time/money and improve patient experience in a constrained environment, particularly lack of funding and understaffing. Inevitable challenges – mainly related to the impact on patient pathways - were resolved and, overall, it was a success. Perceived benefits included reduced patient anxiety, seeing more patients, treating with results, reduced antibiotics use and boosting staff job satisfaction. Learning for other services considering implementing something similar include more inclusive staff engagement, sensitive communication, better documentation of changes, dealing with constant adaptations, and consideration of the impact on staff and their roles.

Figure 1. Overview of rapid pathway service redesign (MSM = Men who have sex with men; NAAT = Nucleic Acid Amplification Test; GC = Gonorrhoea Culture, appt = appointment)

For peer review only

For peer review only - http://bmjopen.bmj.com/site/about/guidelines.xhtml

Acknowledgements

We would like to thank all study participants.

Funding statement

Funded by the National Institute for Health Research (NIHR) Applied Research Collaboration (ARC) West at University Hospitals Bristol and Weston NHS Foundation Trust and NIHR Health Protection Research Unit (HPRU) in Behavioural Science and Evaluation at University of Bristol in partnership with Public Health England (PHE). Award number P315. The views expressed are those of the authors and not necessarily those of the NIHR, the Department of Health and Social Care or PHE.

Competing interests statement

AL – no competing interests
EB - no competing interests
JK - no competing interests
PH - no competing interests
MC - no competing interests
MC - no competing interests
JS - no competing interests
JT - no competing interests
PM - no competing interests
JH - no competing interests

Author contributions

JH, PH, EB, JK were responsible for the study design. JH and EB were responsible for study management and coordination. EB, AL, JK and JH led data collection and analysis. MC and MC co-led the development and implementation of the new service model. PM, JS and JT supported implementation and accreditation of Point of Care testing. MC, MC, JS, JT, PM supported the study design and interpretation of interview findings. All authors read, commented on and approved the final manuscript.

Data Sharing Statement

Data are available on request from corresponding author.

For peer review only

References

1. England PH. Sexually transmitted infections and screening for chlamydia in England, 2019. 2019
2. Public Health England. Sexually transmitted infections (STIs): annual data tables. London: Public Health England, 2018.
3. Mohammed H, Sile B, Furegato M, et al. Poor adherence to gonorrhoea treatment guidelines in general practice in England. *The British journal of general practice : the journal of the Royal College of General Practitioners* 2016;66(648):352. doi: 10.3399/bjgp16x686077
4. Eyre DW, Town K, Street T, et al. Detection in the United Kingdom of the Neisseria gonorrhoeae FC428 clone, with ceftriaxone resistance and intermediate resistance to azithromycin, October to December 2018. *Euro Surveill* 2019;24(10):1900147. doi: 10.2807/1560-7917.ES.2019.24.10.1900147
5. White C. Sexual health services on the brink. *BMJ* 2017;359 doi: 10.1136/bmj.j5395
6. Fifer H, Saunders J, Soni S, et al. 2018 UK national guideline for the management of infection with Neisseria gonorrhoeae. Updated 2020. *International journal of STD & AIDS* 2020;31(1):4-15. doi: 10.1177/0956462419886775 [published Online First: 2019/12/25]
7. Nwokolo NC, Dragovic B, Patel S, et al. 2015 UK national guideline for the management of infection with Chlamydia trachomatis. Updated 2018. *International journal of STD & AIDS* 2018;27(4):251-67. doi: 10.1177/0956462415615443 [published Online First: 2015/11/06]
8. Price MJ, Ades AE, Soldan K, et al. The natural history of Chlamydia trachomatis infection in women: a multi-parameter evidence synthesis. *Health technology assessment (Winchester, England)* 2016;20(22):1-250. doi: 10.3310/hta20220 [published Online First: 2016/03/24]
9. Harding-Esch EM, Fuller SS, Chow SC, et al. Diagnostic accuracy of a prototype rapid chlamydia and gonorrhoea recombinase polymerase amplification assay: a multicentre cross-sectional preclinical evaluation. *Clinical microbiology and infection : the official publication of the European Society of Clinical Microbiology and Infectious Diseases* 2019;25(3):380.e1-80.e7. doi: 10.1016/j.cmi.2018.06.003 [published Online First: 2018/06/16]
10. Van Der Pol B. Making the Most of Point-of-care Testing for Sexually Transmitted Diseases. *Clinical Infectious Diseases* 2019 doi: 10.1093/cid/ciz523
11. Rönn MM, Menzies NA, Gift TL, et al. Potential for Point-of-Care Tests to Reduce Chlamydia-associated Burden in the United States: A Mathematical Modeling Analysis. *Clinical Infectious Diseases* 2019 doi: 10.1093/cid/ciz519
12. Adams EJ, Ehrlich A, Turner KME, et al. Mapping patient pathways and estimating resource use for point of care versus standard testing and treatment of chlamydia and gonorrhoea in genitourinary medicine clinics in the UK. *BMJ Open* 2014;4(7) doi: 10.1136/bmjopen-2014-005322
13. Whitlock GG, Gibbons DC, Longford N, et al. Rapid testing and treatment for sexually transmitted infections improve patient care and yield public health benefits. *International journal of STD & AIDS* 2018;29(5):14. doi: 10.1177/0956462417736431 [published Online First: 2017/10/24]
14. Llewellyn CD, Sakal C, Lagarde M, et al. Testing for sexually transmitted infections among students: a discrete choice experiment of service preferences. *BMJ Open* 2013;3(10) doi: 10.1136/bmjopen-2013-003240
15. Chislett L, Clarke J. Which elements of a novel self-directed rapid asymptomatic sexually transmitted infection screening service are most important to users? . Sexually Transmitted Infections, 2015:A1–A258.
16. Mahilum-Tapay L, Laitila V, Wawrzyniak JJ, et al. New point of care Chlamydia Rapid Test--bridging the gap between diagnosis and treatment: performance evaluation study. *BMJ (Clinical research ed)* 2007;335(7631):1190-94. doi: 10.1136/bmj.39402.463854.AE [published Online First: 11/30]

For peer review only - http://bmjopen.bmj.com/site/about/guidelines.xhtml

17. Llewellyn C, Pollard A, Miners A, et al. Understanding patient choices for attending sexually transmitted infection testing services: a qualitative study. *Sex Transm Infect* 2012;88(7):504-9. doi: 10.1136/sextrans-2011-050344 [published Online First: 2012/05/26]
18. Turner KME, Round J, Horner P, et al. An early evaluation of clinical and economic costs and benefits of implementing point of care NAAT tests for Chlamydia trachomatis and Neisseria gonorrhoea in genitourinary medicine clinics in England. *Sexually Transmitted Infections* 2013 doi: 10.1136/sextrans-2013-051147
19. Natoli L, Guy RJ, Shephard M, et al. Public health implications of molecular point-of-care testing for chlamydia and gonorrhoea in remote primary care services in Australia: a qualitative study. *BMJ Open* 2015;5(4) doi: 10.1136/bmjopen-2014-006922
20. Rompalo AM, Hsieh Y-H, Hogan T, et al. Point-of-care tests for sexually transmissible infections: what do 'end users' want? *Sexual health* 2013;10(6):541-45. doi: 10.1071/SH13047
21. Horwood J, Ingle SM, Burton D, et al. Sexual health risks, service use, and views of rapid point-of-care testing among men who have sex with men attending saunas: a cross-sectional survey. *International journal of STD & AIDS* 2016;27(4):273-80. doi: 10.1177/0956462415580504 [published Online First: 2015/04/25]
22. Lorenc T, Marrero-Guillamón I, Aggleton P, et al. Promoting the uptake of HIV testing among men who have sex with men: systematic review of effectiveness and cost-effectiveness. *Sexually Transmitted Infections* 2011;87(4):272-78. doi: 10.1136/sti.2010.048280
23. Read TRH, Hocking JS, Bradshaw CS, et al. Provision of rapid HIV tests within a health service and frequency of HIV testing among men who have sex with men: randomised controlled trial. *BMJ : British Medical Journal* 2013;347 doi: 10.1136/bmj.f5086
24. Public Health England. Strategic framework to promote the health and wellbeing of gay, bisexual and other men who have sex with men 2014a [Available from: https://www.gov.uk/government/uploads/system/uploads/attachment_data/file/313692/Strategic_Framework_to_promote_the_health_and_wellbeing_of_MSM_FINAL_DRAFT_For_comment.pdf.
25. Fuller SS, Pacho A, Broad CE, et al. "It's not a time spent issue, it's a 'what have you spent your time doing?' issue. . ." A qualitative study of UK patient opinions and expectations for implementation of Point of Care Tests for sexually transmitted infections and antimicrobial resistance. *PloS one* 2019;14(4):16. doi: 10.1371/journal.pone.0215380
26. Widdice L, Hsieh YH, Silver B, et al. Performance of the Atlas Genetics Rapid Test for Chlamydia trachomatis and women's attitudes toward point-of-care testing. *Sexually Transmitted Diseases* 2018;45(11):5. doi: 10.1097/OLQ.0000000000000865
27. Hsieh YH, Hogan MT, Barnes M, et al. Perceptions of an ideal point-of-care test for sexually transmitted infections--a qualitative study of focus group discussions with medical providers. *PloS one* 2010;5(11):e14144. doi: 10.1371/journal.pone.0014144 [published Online First: 2010/12/15]
28. Hsieh Y-H, Gaydos CA, Hogan MT, et al. What Qualities Are Most Important to Making a Point of Care Test Desirable for Clinicians and Others Offering Sexually Transmitted Infection Testing? *PloS one* 2011;6(4):e19263. doi: 10.1371/journal.pone.0019263
29. Hall C, Carter N, Crofts M, et al. P. P155 "time in clinic" survey to evaluate the potential for use of online registration. . *Sexually Transmitted Infections* 2015;91:A66-A67.
30. Huntington SE, Burns RM, Harding-Esch E, et al. Modelling-based evaluation of the costs, benefits and cost-effectiveness of multipathogen point-of-care tests for sexually transmitted infections in symptomatic genitourinary medicine clinic attendees. *BMJ Open* 2018;8
31. Natoli L, Guy RJ, Shephard M, et al. "I Do Feel Like a Scientist at Times": A Qualitative Study of the Acceptability of Molecular Point-Of-Care Testing for Chlamydia and Gonorrhoea to Primary Care Professionals in a Remote High STI Burden Setting. *PloS one* 2016;10(12):e0145993. doi: 10.1371/journal.pone.0145993

For peer review only - http://bmjopen.bmj.com/site/about/guidelines.xhtml

32. Turner KME, Looker KJ, Syred J, et al. Online testing for sexually transmitted infections: A whole systems approach to predicting value. *PloS one* 2019;14(2):e0212420. doi: 10.1371/journal.pone.0212420 [published Online First: 2019/02/23]
33. Murray E, Treweek S, Pope C, et al. Normalisation process theory: a framework for developing, evaluating and implementing complex interventions. *BMC medicine* 2010;8:63. doi: 10.1186/1741-7015-8-63 [published Online First: 2010/10/22]
34. Higginbottom GM, Pillay JJ, Boadu NY. Guidance on Performing Focused Ethnographies with an Emphasis on Healthcare Research *The Qualitative Report* 2013;18(9):1-6.
35. Reeves S, Kuper A, Hodges BD. Qualitative research methodologies: ethnography. *BMJ* 2008;337 doi: 10.1136/bmj.a1020
36. MacGregor L, Speare N, Nicholls J, et al. The role of the evolving trends in sexual behaviours and clinic attendance patterns on increasing diagnoses of STIs: a perspective from Bristol, UK. *Sexually Transmitted Infections* 2020;Accepted for Publication
37. Malterud K, Siersma VD, Guassora AD. Sample Size in Qualitative Interview Studies:Guided by Information Power. *Qualitative Health Research* 2016;26(13):1753-60. doi: 10.1177/1049732315617444
38. Emerson R, Fretz R, Shaw L. Writing ethnographic fieldnotes. Chicago and London: University of Chicago Press 1995.
39. Braun V, Clarke V. One size fits all? What counts as quality practice in (reflexive) thematic analysis? *Qualitative Research in Psychology* 2020:1-25. doi: 10.1080/14780887.2020.1769238
40. Public Health England. Guidance on the collection and reporting of data for GUMCAD, the surveillance system for sexually transmitted infections (STIs) in England. 2019 [Available from: https://www.gov.uk/guidance/gumcad-sti-surveillance-system
41. Horner P, Blee K, O'Mahony C, et al. 2015 UK National Guideline on the management of non-gonococcal urethritis. *International journal of STD & AIDS* 2016;27(2):85-96. doi: 10.1177/0956462415586675 [published Online First: 2015/05/24]
42. Turner KME, Zienkiewicz AK, Syred J, et al. Web-Based Activity Within a Sexual Health Economy: Observational Study. *J Med Internet Res* 2018;20(3):e74. doi: 10.2196/jmir.8101
43. Mohiuddin S, Gardiner R, Crofts M, et al. Modelling patient flows and resource use within a sexual health clinic through discrete event simulation to inform service redesign. *BMJ Open* 2020;10(7):e037084. doi: 10.1136/bmjopen-2020-037084 [published Online First: 2020/07/10]
44. Whitlock GG, Gibbons DC, Longford N, et al. Rapid testing and treatment for sexually transmitted infections improve patient care and yield public health benefits. *International journal of STD & AIDS* 2018;29(5):474-82. doi: 10.1177/0956462417736431 [published Online First: 2017/10/24]
45. Keizur EM, Goldbeck C, Vavala G, et al. Safety and Effectiveness of Same-Day Chlamydia trachomatis and Neisseria gonorrhoeae Screening and Treatment Among Gay, Bisexual, Transgender, and Homeless Youth in Los Angeles, California, and New Orleans, Louisiana. *Sex Transm Dis* 2020;47(1):19-23. doi: 10.1097/olq.0000000000001088 [published Online First: 2019/11/07]
46. Natoli L, Guy RJ, Shephard M, et al. Public health implications of molecular point-of-care testing for chlamydia and gonorrhoea in remote primary care services in Australia: a qualitative study. *BMJ Open* 2015;5(4):e006922. doi: 10.1136/bmjopen-2014-006922 [published Online First: 2015/04/30]
47. Girometti N, Delpech V, McCormack S, et al. The success of HIV combination prevention: The Dean Street model. *HIV medicine* 2021 doi: 10.1111/hiv.13149 [published Online First: 2021/07/30]

For peer review only - http://bmjopen.bmj.com/site/about/guidelines.xhtml

48. Dixon-Woods M, McNicol S, Martin G. Ten challenges in improving quality in healthcare: lessons from the Health Foundation's programme evaluations and relevant literature. *BMJ Quality & Safety* 2012;21(10):876-84. doi: 10.1136/bmjqs-2011-000760
49. Boog K, Heanue J, Kumar V. Implementing integrated sexual and reproductive healthcare in a large sexual health service in England: challenges and opportunities for the provider. *BMJ Sexual & Reproductive Health* 2019;45(2):164-67. doi: 10.1136/bmjsrh-2018-200090
50. Gollop R, Whitby E, Buchanan D, et al. Influencing sceptical staff to become supporters of service improvement: a qualitative study of doctors' and managers' views. *Quality and Safety in Health Care* 2004;13(2):108-14. doi: 10.1136/qshc.2003.007450
51. The Kings Fund. Understanding NHS financial pressures: How are they affecting patient care?, 2017.
52. World Health Organisation. Emergence of multi-drug-resistant Neisseria gonorrhoeae—threat of global rise in untreatable sexually transmitted infections, 2012.
53. Public Health England. PHE infectious diseases strategy 2020 to 2025, 2019.
54. Town K, Harris S, Sánchez-Busó L, et al. Genomic and Phenotypic Variability in Neisseria gonorrhoeae Antimicrobial Susceptibility, England. *Emerg Infect Dis* 2020;26(3):505-15. doi: 10.3201/eid2603.190732
55. Zienkiewicz AK, Verschueren van Rees N, Homer M, et al. Agent-based modelling study of antimicrobial-resistant Neisseria gonorrhoeae transmission in men who have sex with men: towards individualised diagnosis and treatment. *Sex Health* 2019;16(5):514-22. doi: 10.1071/sh18235 [published Online First: 2019/09/03]
56. Toskin I, Govender V, Blondeel K, et al. Call to action for health systems integration of point-of-care testing to mitigate the transmission and burden of sexually transmitted infections. *Sex Transm Infect* 2020;96(5):342-47. doi: 10.1136/sextrans-2019-054358 [published Online First: 2020/04/04]

For peer review only

For peer review only - http://bmjopen.bmj.com/site/about/guidelines.xhtml

Table 1: Quotes on Coherence and Cognitive Participation	
**Coherence**	**Cognitive participation**
**Staff enthusiasm and concerns**<br>It all sounded quite exciting and I was quite, not excited, but it was like, this is really good, the first one in the UK and, you know, this will be excellent, so I was quite open minded about it... lowering the use of antibiotics and fewer invasive procedures for women. (Nurse 17, T3)<br>It's an exciting opportunity, um, but also there's a bit of, um, er, you know, nerves I suppose about how it will actually be, actually run from day one, really, and how it would go (Public Health 14, T2)	**Disengaged staff**<br>It was very much the higher-up staff that kind of organised it all and they're not really the ones that are going to be doing the actual work, so I think it's really important to include clinical staff of all levels, kind of when it's getting near to it and you know, really explain, and have their opinions and thoughts on, you know, how it's gonna work (Nurse 16, T2)
**Reduced patient anxiety**<br>Results on the same day would be amazing, yeah, no doubt about that yeah, because there's always quite an – well, for me, it's like an anxious wait otherwise. Yeah, that sense of not knowing and actually "how do I manage my sex life in case anything comes back positive?" Yeah, quick results can definitely make a big difference (Mike, didn't use rapid STI service)<br>The thing that would encourage me to test more regularly, [is] if the whole thing [time spent in clinic] could be done in a shorter time slot for me (Ben, used rapid STI service)	**Barriers to engagement**<br>[Clinic managers need to] canvass people's feelings about it because I don't know that we do that very well. I think we just crack on. We don't say 'how was it for you that first week did you cope? Did you keep your head above water?' (Nurse 10, T1)

For peer review only

**Avoiding inappropriate antibiotics prescriptions**	**Inadequate preparation**
With antibiotics I'm really – they really mess with my stomach. I really feel really sick whenever I do take them. So, I'm just pro not taking them for that reason alone. Yeah, like – I try to think about the bigger picture of the world and stuff…but I think about my own stomach more than the wider world. So, yeah, I'd – I'm always pleased to like not have to do any unnecessary drugs (Andy, results by text)<br>I just think I'd hate to kind of like take something that I didn't have and then if I ever got it again it doesn't work - so it's kind of like half society view half personal view (Harry, follow-up appointment)	We were told on the Wednesday that it was supposed to be starting on the Monday and we were like all a bit shocked thinking 'well hang on a minute what about the training? We'll look really, really stupid in front of patients' (Administrative staff 6, T1)<br>There was always talk about what it [Panther] could do, never talk about how it's going to function. Even at the last minute, the week before it was meant to start [Lead Consultant] came to me and goes, 'This is what I think the pathway is. Can you make notes on it?' Nobody was really clear about what happened. [Project manager] had sent a PowerPoint around and it was embedded in a way that… some staff who aren't maybe familiar with how embedded links and things work [- couldn't open it]. When the meeting came around, they said, 'Has everyone read the email about the Pathways?' half of the people went, 'What email?' …, it just wasn't presented to us in a clearest way. (Health Adviser 1, T2)

For peer review only

<table>
<tr><th colspan="4">Table 2: Changes along the (walk-in) male patient pathway</th></tr>
<tr><th></th><th>Before rapid STI service</th><th colspan="2">Rapid STI service</th></tr>
<tr><td rowspan="3">Registering at reception</td><td rowspan="2">All walk-in patients allocated an appointment in order of queue – may have to come back later that day.</td><td colspan="2">Receptionist triages each patient to appropriate pathway referring to guide/pathway</td></tr>
<tr><td>Patients not eligible for rapid STI service are given an appointment for later that day and continue on old pathway</td><td>Rapid STI service-eligible patients wait after registering to be called up.</td></tr>
<tr><td>Triage form used to register patients.</td><td colspan="2">Triage form amended to be gender neutral and to make categories clearer.</td></tr>
<tr><td rowspan="3">Seeing a clinician/ health adviser</td><td>At first appointment</td><td colspan="2">First appointment very brief– reduced history taking. Unless uncomplicated vaginal discharge.</td></tr>
<tr><td rowspan="2">MSM and all patients needing partner notification/ risk reduction/safer sex advice see health adviser</td><td colspan="2">High-risk patients (MSM or new to service) see health adviser at initial appointment.</td></tr>
<tr><td colspan="2">Symptomatic men see doctor/trained nurse only at follow-up not drop-off, usually on same day.</td></tr>
<tr><td>Providing samples</td><td>Urine and swabs: taken at time of consultation. Self-taken if asymptomatic. If symptomatic: clinician taken swab for microscopy (to detect NGU and gonorrhoea) and gonorrhoea culture; NAAT self-taken</td><td colspan="2">Self-sample drop-off - Urine and swabs NAAT self-sampled in toilets, putting samples through a hatch to the lab. Instructions are on posters in the toilet and from nursing staff/NAs. Gonorrhoea culture taken by clinician on return only if NAAT positive. Swab for microscopy to detect NGU if NAAT-negative (see text above).</td></tr>
<tr><td></td><td>Blood samples taken by doctor/nurse/nursing assistant (NA)</td><td colspan="2">Blood samples taken by doctor/nurse/NA</td></tr>
<tr><td rowspan="3">Tests results</td><td>All STI test results in 2 -3 weeks</td><td colspan="2">Chlamydia and gonorrhoea processed on Panther - results within 48 hours. Others still 2-3 weeks</td></tr>
<tr><td>If negative, text sent</td><td colspan="2">Results by text if asymptomatic and negative.</td></tr>
<tr><td>If positive, HA phones patient to discuss result and arrange treatment (unless already received presumptive treatment).</td><td colspan="2">Results given at follow up appointment with clinician if symptomatic or asymptomatic positive.</td></tr>
<tr><td rowspan="2">Treatment</td><td>Treat presumptively</td><td colspan="2">Wait for results before treating</td></tr>
<tr><td>Treat at first (only) appointment</td><td colspan="2">Treat at follow-up appointment</td></tr>
<tr><td>Notified partners</td><td>Treated immediately</td><td colspan="2">Only treat on positive results (if sexual contact was >2 weeks; otherwise treat immediately)</td></tr>
</table>

For peer review only - http://bmjopen.bmj.com/site/about/guidelines.xhtml

Table 3: Collective action quotes
**Designing and documenting new processes**
We need to be really clear about what we're doing when they drop-off [patients drop-off samples], what we're doing when they come back, what are we going to do about contraception, which questions are okay to leave out of the proforma. ..for consultants because we have a lot of experience and because we're used to making decisions then I think we can [unclear] a bit and we can be flexible and can you know think about the individual patient. But for nurses who work a lot to PGD's [guidelines] and like to have clear guidance. And some of the juniors as well, who will be quite new - you know they've just been changed. Because it's going to be bewildering and chaotic you know it's doesn't feel good when there's chaos on the shop floor. (Doctor 3, T1)
**Flexibility in pathways**
It's a case by case situation and it does help to have helpful medical staff that have been willing to make an exception. (Administrative staff 18, T2)<br><br>I think the health advisers also are more able to …know the guidelines but in some situations know that you have to approach things differently, … for me personally if I was seeing someone and they kind of said 'actually I have got this dis[ease]' - you know, real clear symptoms, you know, 'and I'm really fed up with it', I'd be more inclined to say 'okay then let's get you treated…I think you can have your general thing of saying to someone 'look you know come back in the afternoon' but if you've got someone who's kind of 'actually no but I've had these symptoms for two days I've really had enough of it'. (Health Adviser 13, implementation group member, T2)
**Guidelines**
It's a work in progress but the problem is as the pathway evolves then the guideline will change again…because this is so rapidly moving actually, I don't think I really want to do a guideline. So it's kind of hard to have a guideline anyway but we need some kind of guidance. (Doctor 3, T1)
**Teething issues**
It was chaos, the first few weeks were chaos. Reception didn't know what they were doing… there was hundreds of patients around the reception, we didn't know what we were doing, so yeah, it was chaos, but it has slowly got better. (Nurse 17, T2)<br><br>The waiting area fills up and people are filling out the Panther triage forms on windowsills. After a while [clinic coordinator] tells receptionists on Panther desk that he had given out 16 forms. Once they get to 10 people booked for Panther returner pathway they need to go check with the lab regarding further capacity. (Observation notes, Reception area)<br><br>Two reception staff were unsure whether one person should be panther/same day or walk-in due to the information provided on the form. Staff consulted with person entering data on computer. They checked whether person was returning for results/treatment. They explained to the patient that a new system is in place, so they want to make sure they do the best for him. (Observation notes, Reception area)
**Understaffing**
It's been very stressful for staff and I think it has been an enormous amount of work for the implementation group, that I think in the private industry you'd be given huge amounts of time, whereas we virtually squeezed it in amongst everything else we've done, but that's just the NHS. (Doctor 11, T2)<br><br>Administrative staff / reception team has three staff vacancies, and today there are two members of clinic staff off sick – one clinician, and one Administrative staff (clinic coordinator). The clinician

For peer review only - http://bmjopen.bmj.com/site/about/guidelines.xhtml

would have been doing sample drop-off, and walk-in, so have had to reduce slots for both until they get confirmation of clinical capacity from clinicians when they arrive. (Observation notes, Reception area)

Changing ingrained behaviours

It's been quite hard on staff and obviously there's a lot of – you know, if you've been doing something the same way for 10/20/maybe 30 years, that's quite a massive change for people. (Nurse 21, T3)

Changes to clinician contact

We've actually ended up seeing a lot more complicated or complex patients, at least that's how it feels. The easy patients get siphoned off quite quickly and that means that more patients [can be seen], especially the complex patients, which the nurses are less able to deal with and require a lot more consultant supervision. I think there has been a general feeling in the department that the consultant cover job is busier than it ever was before. (Doctor 11, T2)

Challenges of changes at reception

[Receptionists] were worried that they were looking like they didn't know what they were doing, because it was new and they weren't quite sure. So I think it took a, it was a lot to ask for them all really because it was a big change, but it is just that keep reminding everybody that actually, in the long term, you will get it, and it's much better for other patients once it's in place. (Administrative staff 12, implementation group member, T2)

Reception staff on male desk refresh the panther decision pathway together using A4 sheet. Female staff member commented that she always has to double check the process. Reception staff discussed male staff member's confusion about eligibility for Panther. (Observation notes, Reception area)

Concern about shorter consultations

It was a huge change, because, we, it is quite a detailed consultation. We have been told time and time again that 'oh you need to ask patients about domestic violence, ask the women about female genital mutilation, you need to do this'. Then all of a sudden, they are saying, 'no, don't ask any of these things', it's like aargh! (Nurse 17, T2)

It does sometimes feel if I'm absolutely honest a little bit less than a level three service, you know, people are just coming in and dropping off a sample. I know that's possibly better use of our time, but it seems a little bit spurious to call it level three. (Nurse 10, T1)

Perceived patient views on waiting for treatment

I think the major anxiety that patients have is around not being treated immediately and not being treated necessarily as a contact of infection and anxiety around that. I often find that with a bit of educating that that is overcome and my major impression is that patients really appreciate it. (Doctor 11, T2)

I haven't had anybody who's been absolutely, you know, anti about it but there have been a couple of people who I've thought 'I'm going to treat you mate, I'm not going to wait on results' do you know what I mean? ... they're anxious, they've maybe got another partner, a regular partner, who they don't want to infect, which, you know, I can see the reasoning behind that. But I think, you know, once that kind of idea has got out amongst our regular clientele I think it will be a lot easier. (Nurse 10, T1)

Four young men approach the door together. [Name] lets them know that he has just 2 forms/slots left at present, and suggests that he gives these to them on the basis of who came through the door first – these two seem pleased, and head in with their forms. He asks the other two to wait here for a minute and they seem OK with this. He tells them there is a new service

For peer review only

which means they can give people results/treatment faster, and this is why things are different. He asks them to give him a yes/no answer as to whether they have any symptoms. When they say no he says that it is probably not worth them waiting as they are unlikely to be seen today, and that they could come back another morning for when the doors first open. They seem to find this acceptable. (Observation notes, front door)
**Benefit of evaluation process**   Staff member commented that it was helpful to have an outside voice (research team) feeding back, because sometimes when you are within the structure you can be shouting stuff and nobody hears you. (Observation notes)

**Table 4: Reflexive monitoring quotes**
**Success**   When you speak at the national [sexual health] meetings, people, it's a bit of a no-brainer, what we're supposed to do, and people are amazed that we've been able to introduce it [rapid STI service] cost-neutrally. Because when you look at the point-of-care systems which other people are researching, it's… more expensive, so we've adopted an innovative approach. (Doctor 19, T3)
**Quality of care**   The person I saw was really brilliant, like, yeah. I felt really comfortable… I really felt like I could ask anything I want and felt sort of safe (Andy, results by text1)
**Benefits**   It was the immediacy and the kind of reassurance that … if something was positive that you would be able to treat it straightaway. (Harry, follow-up appointment)   They're [patients] very happy. I mean, who wouldn't be? You find out the same day that you have got chlamydia and you can start your treatment. I mean that is brilliant. (Nurse 17, T3)   Dr 11: One of the advantages that we hoped would come out of introducing Panther would be that we would attract more high-risk people, because it would be seen as an attractive place to come and test, and also that it would free up staff time so that we could spend more time with risk reduction etc.   Interviewer: Do you think that is happening? Or is it not there yet?   Dr 11: I think it has started to happen, I don't think that's only down to Panther, I think that's down to some other stuff like PrEP and things like that as well. I feel like the cohort of patients that we see is increasingly complex. (Doctor 11, T2)   I think the most positive things are seeing your symptomatic patients with knowing what is going on with them. You know what infection they have, you know what treatment they require or if they don't have anything you can then take the time to discuss that. (Doctor 11, T2)   I think it's [rapid service implementation] made the staff more able to deal with change [to telephone clinics], because they had undergone experience of change with Panther pathways over the past 12-18 months…. Yes, it probably made it smoother and more efficient. (Doctor 22,T4)
**Suggested improvements**   The main issues that have arisen have been when the [Panther] machine fails and that can be pretty catastrophic (laughs), just because you have booked slots and patients come back and you don't, you can't even tell them whether they have chlamydia or gonorrhoea and they've, kind of, come with that expectation. (Doctor 20, T3)

For peer review only

Interviewer: If you had an imaginary clinic who were going to set out on this path, what would your advice be to them overall? With the knowledge that you've now gathered from your experience, is there a way that you could help them?

Dr 11: I think preparation, preparing all of the documents that support your staff on a day-to-day basis, clarifying your communication pathways, giving your lead clinicians adequate time in their work plans to do all of that. I would definitely support it, I think it's definitely been a major benefit. (Doctor 11, T2)

It has been a big change for all staff working, and it's difficult to know whether there was any way of realising some of the things we hadn't realised. I don't think we could have done. I think they were literally just things of implementation that have caused some additional tweaks required - and that in itself has been stressful because it's been the realisation of what are we doing in this scenario and not being quite prepared for it. (Administrative staff 12, T2)

The thing that I found most interesting is the communication difficulties in amongst the staff and how difficult that has been, having an implementation group that I think represents most of the groups that it's impacted upon and the difficulty that the messages just have not got to the clinic floor, and that's an on-going issue. (Doctor 11, T2)

For peer review only

Pathway stage	Usual care pathway	Rapid pathway
**Reception**	- Walk-in patients' queue as clinic opens and appt are allocated on first-come-first-served basis	Patients are triaged to eligible for rapid STI service or not eligible.<br>• Eligible – Registered<br>• Non-eligible - continue along old pathway
**Clinical review**	Appt with clinician, full history taken.	Brief history taken at initial appt<br>MSM referred to Health Adviser<br>If follow up appt needed then full history taken at that time
**Sample testing**	Asymptomatic: Self-taken samples<br>Symptomatic: Clinician-taken samples for NAAT, GC culture and microscopy (as appropriate)	Self-taken NAAT testing **only** at initial appt<br>GC cultures taken **only** if GC positive on NAAT<br>Samples taken for microscopy only if NAAT negative and symptomatic
**Test results**	All test results available after approximately 10-14 days	Symptomatic: results available within the same day (triggering same/next day follow-up appt)<br>Asymptomatic: within 48 hours
**Treatment**	Patients treated syndromically prior to NAAT results.<br>Patient return for infection-specific treatment when test results are known (10-14 days)	Infection-specific treatment provided same/next day based on rapid test results
**Partner notification**	Started when positive test results returned (at 10-14 days)	Immediately commenced at same/next day follow-up appt

Figure 1. Overview of rapid pathway service redesign (MSM = Men who have sex with men; NAAT = Nucleic Acid Amplification Test; GC = Gonorrhoea Culture, appt = appointment)

For peer review only - http://bmjopen.bmj.com/site/about/guidelines.xhtml

Elements of rapid STI service implementation	
**What**	**Who should do this**
**Changes to documentation**	
Rewrite and sign-off treatment guidelines and SOPs when new processes are agreed.	Project leads, clinical lead, project implementation/operations team and clinical governance guideline group
Change the triage process and form.	Project operations team, to include reception staff
Consider changes to IT system/medical records system.	Project leads, consultant and project implementation team
**Implementation of the actual machine and process**	
Write business case for new rapid STI service and have it signed off by PHE.	Clinic lead, clinic manager, lead consultant, project manager, in collaboration with PHE.
Source the machine, find space for it (with waste disposal) and install it. Arrange insurance (including negotiations with PHE and legal teams)	Clinic lead, operations manager, lead consultant, PHE, nursing lead
Ensure IT systems allow direct transfer of data from Panther	Project lead, clinic manager
Pilot before implementing with all patients	All staff including reception teams
Write protocol for Panther outages	Project leads, in collaboration with PHE.
Quality assessment scheme /UKAS accreditation	PHE team
**Services**	
Consider impact on other services	Project operations team
Adjust clinic timetabling to accommodate rapid STI service appointments	Operations manager
Changing medical history forms and process to accommodate the new appointment structure	Project leads and implementation team
Changes to the IT coding	Clinic data manager, project lead, clinic manager, clinical lead (minor)
**Staff engagement, training and communication**	
Put together an implementation team, to oversee implementation, and put in place mechanisms for all staff to feedback to this team	Representative from each staff group and clinic manager.
Clarify communication pathways between all staff and the implementation team	Clinic Manager, project leads
Consider the impact on staff roles and workload and if staffing changes are therefore needed	Project operations team
Regular meetings for staff involved in the new service	Possible staff to include: project leads, HAs, consultants, nursing assistants, nurses, administrative staff, researchers, IT lead, clinic manager, data manager, chlamydia screening program team lead.
Staff training and regular updates at existing staff training sessions	Led by project leads, all staff to attend
Regular departmental meetings	Project leads and clinical lead
**Patient communication**	
Communicate changes to patients – write leaflets/posters/website	Project leads

For peer review only

COREQ (COnsolidated criteria for REporting Qualitative research) Checklist

A checklist of items that should be included in reports of qualitative research. You must report the page number in your manuscript where you consider each of the items listed in this checklist. If you have not included this information, either revise your manuscript accordingly before submitting or note N/A.

Topic	Item No.	Guide Questions/Description	Reported on Page No.
**Domain 1: Research team and reflexivity**			
Personal characteristics			
Interviewer/facilitator	1	Which author/s conducted the interview or focus group?	
Credentials	2	What were the researcher's credentials? E.g. PhD, MD	
Occupation	3	What was their occupation at the time of the study?	
Gender	4	Was the researcher male or female?	
Experience and training	5	What experience or training did the researcher have?	
Relationship with participants			
Relationship established	6	Was a relationship established prior to study commencement?	
Participant knowledge of the interviewer	7	What did the participants know about the researcher? e.g. personal goals, reasons for doing the research	
Interviewer characteristics	8	What characteristics were reported about the inter viewer/facilitator? e.g. Bias, assumptions, reasons and interests in the research topic	
**Domain 2: Study design**			
Theoretical framework			
Methodological orientation and Theory	9	What methodological orientation was stated to underpin the study? e.g. grounded theory, discourse analysis, ethnography, phenomenology, content analysis	
Participant selection			
Sampling	10	How were participants selected? e.g. purposive, convenience, consecutive, snowball	
Method of approach	11	How were participants approached? e.g. face-to-face, telephone, mail, email	
Sample size	12	How many participants were in the study?	
Non-participation	13	How many people refused to participate or dropped out? Reasons?	
Setting			
Setting of data collection	14	Where was the data collected? e.g. home, clinic, workplace	
Presence of non-participants	15	Was anyone else present besides the participants and researchers?	
Description of sample	16	What are the important characteristics of the sample? e.g. demographic data, date	
Data collection			
Interview guide	17	Were questions, prompts, guides provided by the authors? Was it pilot tested?	
Repeat interviews	18	Were repeat inter views carried out? If yes, how many?	
Audio/visual recording	19	Did the research use audio or visual recording to collect the data?	
Field notes	20	Were field notes made during and/or after the inter view or focus group?	
Duration	21	What was the duration of the inter views or focus group?	
Data saturation	22	Was data saturation discussed?	
Transcripts returned	23	Were transcripts returned to participants for comment and/or	

For peer review only

Topic	Item No.	Guide Questions/Description	Reported on Page No.
		correction?	
**Domain 3: analysis and findings**			
Data analysis			
Number of data coders	24	How many data coders coded the data?	
Description of the coding tree	25	Did authors provide a description of the coding tree?	
Derivation of themes	26	Were themes identified in advance or derived from the data?	
Software	27	What software, if applicable, was used to manage the data?	
Participant checking	28	Did participants provide feedback on the findings?	
Reporting			
Quotations presented	29	Were participant quotations presented to illustrate the themes/findings? Was each quotation identified? e.g. participant number	
Data and findings consistent	30	Was there consistency between the data presented and the findings?	
Clarity of major themes	31	Were major themes clearly presented in the findings?	
Clarity of minor themes	32	Is there a description of diverse cases or discussion of minor themes?	

Developed from: Tong A, Sainsbury P, Craig J. Consolidated criteria for reporting qualitative research (COREQ): a 32-item checklist for interviews and focus groups. *International Journal for Quality in Health Care*. 2007. Volume 19, Number 6: pp. 349 – 357

Once you have completed this checklist, please save a copy and upload it as part of your submission. DO NOT include this checklist as part of the main manuscript document. It must be uploaded as a separate file.

For peer review only

What can be learnt from a qualitative evaluation of implementing a rapid sexual health testing, diagnosis and treatment service?

Journal:	*BMJ Open*
Manuscript ID	bmjopen-2021-050109.R2
Article Type:	Original research
Date Submitted by the Author:	11-Sep-2021
Complete List of Authors:	Lorenc, Ava; University of Bristol, Population Health Sciences, Bristol Medical School; National Institute for Health Research, Applied Research Collaboration West<br>Brangan, Emer; University of the West of England, Department of Nursing and Midwifery<br>Kesten, Joanna; University of Bristol, National Institute for Health Research, Applied Research Collaboration West (NIHR ARC West); University of Bristol, NIHR Health Protection Research Unit in Behavioural Science and Evaluation, Population Health Sciences, Bristol Medical School<br>Horner, Paddy; University of Bristol, NIHR Health Protection Research Unit in Behavioural Science and Evaluation, Population Health Sciences, Bristol Medical School; University Hospitals Bristol and Weston NHS Foundation Trust, Unity Sexual Health<br>Clarke, Michael; University Hospitals Bristol and Weston NHS Foundation Trust, Unity Sexual Health<br>Crofts, Megan; University Hospitals Bristol and Weston NHS Foundation Trust, Unity Sexual Health<br>Steer, Jonathan; Public Health England, South West Regional Laboratory, National Infection Service<br>Turner, Jonathan; Public Health England, South West Regional Laboratory, National Infection Service<br>Muir, Peter; Public Health England, South West Regional Laboratory, National Infection Service<br>Horwood, Jeremy; University of Bristol, National Institute for Health Research, Applied Research Collaboration West (NIHR ARC West), University Hospitals Bristol and Weston NHS Foundation Trust; University of Bristol, NIHR Health Protection Research Unit in Behavioural Science and Evaluation, Population Health Sciences
**Primary Subject Heading**:	Sexual health
Secondary Subject Heading:	Health services research, Qualitative research
Keywords:	QUALITATIVE RESEARCH, SEXUAL MEDICINE, Organisational development < HEALTH SERVICES ADMINISTRATION & MANAGEMENT, Change management < HEALTH SERVICES ADMINISTRATION & MANAGEMENT, COVID-19

SCHOLARONE™
Manuscripts

I, the Submitting Author has the right to grant and does grant on behalf of all authors of the Work (as defined in the below author licence), an exclusive licence and/or a non-exclusive licence for contributions from authors who are: i) UK Crown employees; ii) where BMJ has agreed a CC-BY licence shall apply, and/or iii) in accordance with the terms applicable for US Federal Government officers or employees acting as part of their official duties; on a worldwide, perpetual, irrevocable, royalty-free basis to BMJ Publishing Group Ltd ("BMJ") its licensees and where the relevant Journal is co-owned by BMJ to the co-owners of the Journal, to publish the Work in this journal and any other BMJ products and to exploit all rights, as set out in our licence.

The Submitting Author accepts and understands that any supply made under these terms is made by BMJ to the Submitting Author unless you are acting as an employee on behalf of your employer or a postgraduate student of an affiliated institution which is paying any applicable article publishing charge ("APC") for Open Access articles. Where the Submitting Author wishes to make the Work available on an Open Access basis (and intends to pay the relevant APC), the terms of reuse of such Open Access shall be governed by a Creative Commons licence – details of these licences and which Creative Commons licence will apply to this Work are set out in our licence referred to above.

Other than as permitted in any relevant BMJ Author's Self Archiving Policies, I confirm this Work has not been accepted for publication elsewhere, is not being considered for publication elsewhere and does not duplicate material already published. I confirm all authors consent to publication of this Work and authorise the granting of this licence.

For peer review only

What can be learnt from a qualitative evaluation of implementing a rapid sexual health testing, diagnosis and treatment service?

Authors

Ava Lorenc[1,2], Emer Brangan[1,3], Joanna M Kesten[1,2,4], Paddy Horner[1,4,5], Michael Clarke[5], Megan Crofts[5], Jonathan Steer[6], Jonathan Turner[6], Peter Muir[6], Jeremy Horwood[1,2,4]

[1.] Population Health Sciences, Bristol Medical School, University of Bristol, Canynge Hall, 39 Whatley Road, Bristol, BS8 2PS

[2.] National Institute for Health Research, Applied Research Collaboration West (NIHR ARC West), University Hospitals Bristol and Weston NHS Foundation Trust, 9th Floor, Whitefriars, Lewins Mead, Bristol, BS1 2NT

[3] University of the West of England
Department of Nursing and Midwifery, Faculty of Health and Applied Sciences, University of the West of England, Frenchay Campus, Coldharbour Lane, Bristol, BS16 1QY

[4] NIHR Health Protection Research Unit in Behavioural Science and Evaluation, Population Health Sciences, Bristol Medical School, University of Bristol, Canynge Hall, 39 Whatley Road, Bristol, BS8 2PS

[5] Unity Sexual Health, University Hospitals Bristol and Weston NHS Foundation Trust, Tower Hill, Avon, Bristol BS2 0JD

[6] Public Health England South West Regional Laboratory, National Infection Service, Pathology Sciences Building, Science Quarter, Southmead Hospital, Bristol BS10 5NB

Corresponding author: Jeremy Horwood, J.Horwood@bristol.ac.uk

Word count: 4521

For peer review only

Abstract

Objectives

To investigate experiences of implementing a new rapid sexual health testing, diagnosis and treatment service.

Design

A theory-based qualitative evaluation with a focused ethnographic approach using non-participant observations and interviews with patient and clinic staff. Normalisation Process Theory was used to structure interview questions and thematic analysis.

Setting

A sexual health centre in Bristol, UK.

Participants

26 patients and 21 staff involved in the rapid sexually transmitted infection (STI) service were interviewed. Purposive sampling aimed for a range of views and experiences and socio-demographics and STI results for patients, job grades and roles for staff. 40 hours of observations conducted.

Results

Implementation of the new service required co-ordinated changes in practice across multiple staff teams. Patients also needed to make changes to how they accessed the service. Multiple small 'pilots' of process changes were necessary to find workable options. For example, the service was introduced in phases beginning with male patients. This responsive operating mode created challenges for delivering comprehensive training and communication in advance to all staff. However, staff worked together to adjust and improve the new service, and morale was buoyed through observing positive impacts on patient care. Patients valued faster results and avoiding unnecessary treatment. Patients reported that they were willing to drop-off self-samples and return for a follow-up appointment, enabling infection-specific treatment in accordance with test results thus improving antimicrobial stewardship.

Conclusions

The new service was acceptable to staff and patients. Implementation of service changes to improve access and delivery of care in the context of stretched resources can pose challenges for staff at all levels. Early evaluation of pilots of process changes, played an important role in the success of the service by rapidly feeding back issues for adjustment. Visibility to staff of positive impacts on patient care is important in maintaining morale.

Strengths and limitations of this study

- The 'trial, assess, adapt' strategy (reflexive process of observation, feedback, and resulting action) meant that evaluation and implementation occurred in parallel and allowed researchers to capture the active process.
- The evaluation benefitted the staff, as researchers provided ongoing feedback and suggestions for service improvements and provided a space for reflection.

For peer review only - http://bmjopen.bmj.com/site/about/guidelines.xhtml

- A strong and trusting relationship between research and clinic staff arose from researcher flexibility and timely responsiveness and allowed good researcher access to spaces, staff and meetings.
- Frequent, regular and extensive physical presence of the researcher in various clinic settings was crucial as much of the process was not documented.
- The patient sample was limited due to recruitment being cut short by the COVID-19 pandemic lockdown, and we only interviewed males due to the pathway being initially implemented for male patients during the evaluation period.

For peer review only

For peer review only - http://bmjopen.bmj.com/site/about/guidelines.xhtml

Introduction

Rates of sexually transmitted infections (STIs) continue to increase in England despite control efforts, with a 5% increase between 2018-2019[1]. *Chlamydia trachomatis* (chlamydia) and *Neisseria gonorrhoeae* (gonorrhoea) are the most common, with 226,411, and 70,982 diagnoses reported in England in 2019, a 5% and 26% increase since 2018[2]. The rise in gonorrhoea is particularly concerning as first line treatment effectiveness is threatened by the development of antimicrobial resistance (AMR)[3,4]. Most STIs are diagnosed through Specialist Sexual Health Services (SSHS), the provision of which is increasingly challenging as funding (via government public health grant), has been steadily cut since 2015[5].

Chlamydia and gonorrhoea if left untreated may cause pelvic inflammatory disease (PID) in women, which can result in infertility, ectopic pregnancy, and chronic pelvic pain[6-8]. Infections are often asymptomatic, particularly in women, and when they do cause symptoms and/or signs these are not pathognomonic[6 7]. Nucleic acid amplification tests (NAATs) provide accurate detection. Early detection and treatment helps prevent the spread of STIs and the development of complications. Point-of-care testing (POCT; results within 15-30mins)[9] and rapid STI services (results on the same day) can potentially improve care and reduce costs, due to reduced time from diagnosis to treatment and number lost to follow up. This can increase testing uptake, improve partner notification rates and enable better and timelier clinician decisions, improving outcomes such as fewer unnecessary treatments and reduced PID risk[10-13]. Patients prefer rapid STI testing[14-16] and are happy to wait at clinic for results. Rapid testing can reduce anxiety[17 18] and improve patient acceptability of services and uptake of testing[19-22]. HIV POCT is well established and preferred by high risk men who have sex with men (MSM)[23 24]. Although studies suggest a limit of 30 minutes to wait for results[25-28], experience from our service indicates patients would be prepared to wait longer than 20 minutes for their result[29].

However, much of the evidence is from modelling and hypothetical views of clinicians and/or patients[10-12 25-28 30], with little real-life implementation evaluation[31], and rarely considering the complexity of patient visits including both asymptomatic and symptomatic patients with multiple needs e.g. female contraception. There is an urgent need to evaluate staff and patient preferences, and clinical benefits and cost effectiveness in practice.

In November 2018, a UK SSHS implemented a first-of-its-kind rapid STI testing, diagnosis and treatment service, using a clinic-based Hologic 'Panther' NAAT diagnostic machine. In 2017, the clinic introduced an online STI and HIV testing postal service for asymptomatic patients[32]. The new rapid service provides chlamydia and gonorrhoea results in 3.5 hours (previously over a week when tested in the microbiology laboratory), to improve patient care while reducing costs (see figure 1 for an overview of the service redesign). This evaluation assessed the best service model and patient and staff acceptability, to refine and improve the service and support implementation in other SSHSs. We report the qualitative evaluation of male patient and staff views and experiences of the implementation of the first phase of this new rapid STI service.

For peer review only - http://bmjopen.bmj.com/site/about/guidelines.xhtml

Methods

Design

The evaluation was ethnographic, used observations and interviews[33] and was informed by Normalisation Process Theory (NPT). NPT is a sociological theory that has been widely promoted as a means to understand implementation, embedding and integration of innovation in healthcare settings until they become normalised and routine[34]. This approach focuses on actions people perform to normalise an intervention within the contexts and locations they inhabit[34]. NPT proposes that successful implementation of an intervention is dependent on participants ability to fulfil four inter-related criteria which interact with the wider intervention context[34]: 1) Coherence - (sense-making - understanding and opinion of the intervention's purpose); 2) Cognitive Participation (commitment and engagement with the intervention); 3) Collective Action (the work that individuals and organisations have to do to make the intervention function); 4) Reflexive Monitoring (appraisal of the intervention once it is in use). NPT supported real-time feedback to refine and improve the service. The study focussed on four timepoints selected pragmatically during 16 months of evaluation: T1 at start of implementation; T2 after 6 months; T3 after 14 months; T4 at 16 months during the COVID-19 pandemic lockdown.

Setting

A sexual health clinic in Bristol (population 450,000), UK.

Participants

Due to the new service being initially introduced for the male pathway only, male patients (over 16 years old) and staff at the sexual health clinic were interviewed. Patients were invited to take part, via a clinic survey about PrEP (pre-exposure prophylaxis for HIV) [35] and when physically attending the clinic at T1, T2 and T3. Cross sectional interviews were conducted with staff at four timepoints at T1, T2, T3, and T4. One staff member was interviewed twice. Purposive sampling[33] attempted to capture maximum variation in views and experiences, and socio-demographics and STI test results for patients, and job grades and roles for staff (administrative staff, consultants, doctors, nurses/nursing assistants, health advisers, Public Health England (PHE)); responsible for the Panther laboratory and administration. Information sheets were provided to male patients by staff at the clinic or via email from researchers, with patients asked to contact the researcher and ask questions before deciding to take part. Staff were emailed by the researcher about the study.

Data collection

Following the concept of information power, data collection continued until sufficient data to meet the study objectives had been collected with continuous, pragmatic assessment of information within our sample [36]. Issues informing information power include the study aim (i.e. broader aims require a larger sample), the sample (i.e. a smaller sample is needed if participants have rich experiences relevant to the research), use of theory (studies supported by theory require smaller sample sizes), depth and quality of the data (i.e. smaller samples are needed with focused and clear data) and the analysis type (larger samples are needed for exploratory analysis) [36].

For peer review only - http://bmjopen.bmj.com/site/about/guidelines.xhtml

In the first 6 months of service implementation, observations were conducted by EB and JK at varying times/days, in reception, laboratory and waiting areas. Non-participant observations focussed on day-to-day operations, how clinic staff integrated the new service and any factors which promoted or inhibited successful incorporation [37]. Written accounts based on brief field notes taken at the time included observations, conversations with staff, and reflection on what had been observed[38]. Observations recorded activities, events, their time and location and described interactions, communication patterns, workflows and tasks in the clinic environment.

Interview topic guides informed by NPT explored: views and experiences of the service; impact on workload and clinical practice; information and support needs, sustainability and future implementation of the service. Patient interviews took place throughout the evaluation period and explored their experience and views of the service including acceptability, barriers and facilitators to uptake. Patients were offered a £10 High Street shopping voucher. Interviews were conducted by experienced qualitative senior research associates AL/JMK/EB, used flexible topic guides and open-ended questioning, were face-to-face (at the clinic or University) or by telephone, and lasted around 30 minutes. Participants were told that the study was evaluating the rapid results service and that interviewers were independent of the service.

Analysis

Interviews were audio recorded, transcribed verbatim and imported into QSR NVivo (version 10) with transcribed observation fieldnotes. Ongoing and iterative analysis informed further data collection through changes to the topic guide and feedback to healthcare staff to aid the adaptation and refinement of the rapid service. 'Codebook' thematic, inductive analysis by EB/AL identified and analysed patterns and themes salient to interviews and observations [39]. Initial noting of ideas was followed by line-by-line examination and inductive coding. A subset of transcripts and observations were independently double-coded by EB/JH and discrepancies discussed to contribute to the generation and refinement of codes to maximise rigour. Themes were discussed by the multi-disciplinary research team to ensure credibility and confirmability. Negative cases and reasons for deviance were explored. The four NPT constructs[34] were used to further develop themes deductively.

Ethical approval

South West Frenchay Research Ethics Committee granted approval, reference 18/SW/0090.

Patient and Public Involvement

PPI meetings with three people who recently used the clinic informed the study design. These meetings reviewed patient-facing materials and discussed the acceptability of proposed recruitment and data collection.

Results

Participants/hours of observation

We conducted 25 observations over approximately 40 hours, 25 staff interviews (24 participants) and 26 patient interviews. Patients were aged 34 years on average (range 19 to 57 years), most

For peer review only - http://bmjopen.bmj.com/site/about/guidelines.xhtml

identified as MSM, two had positive STI test results and the index of multiple deprivation scores averaged 5.4 (range 2 to 10).

Coherence (sense-making)

Staff and patients welcomed rapid testing (Table 1). All staff saw it as beneficial and many were excited about doing something new, particularly to improve service access which was limited by a lack of pre-bookable appointments, high observed demand (manifesting in long queues outside the clinic before it opened each morning to access limited capacity walk-in appointments) and staff shortages. Staff welcomed being able to provide treatment based on results and avoiding unnecessary antibiotic prescribing (previously treatment was prescribed presumptively for symptomatic patients due to the week-long wait for test results). However, some staff had concerns around anticipated reduced clinician contact with patients and shorter consultations in the new service. Patients valued a potentially quicker and more convenient service, but also reduced anxiety from long waiting times for results,. Some patients valued avoiding unnecessary antibiotic treatment for personal and wider societal reasons.

Cognitive participation (buy-in)

The importance of engaging the whole clinic team in the service redesign was recognised but challenging with a large team and many part-time staff (Table 1). Formal engagement was via an implementation team, project meetings and staff training sessions. Engagement of 'on the ground' staff was inadequate, with administrative and nursing staff feeling particularly disengaged and having limited time to prepare for the new service. Staff cited the following issues around engagement:poor communication (due to busy work schedules with limited time for accessing emails), a lack of access to training as many staff were part-time and did not work on the day training was delivered or lack of involvement in project meetings or the implementation group which was initially only senior staff, although the latter improved as the project progressed. Engagement was also limited by a lack of staff protected project time and a context of burn out from staff pressures (e.g. funding cuts, understaffing, and high service demand). Implementation work was fitted around existing high workloads and rapid service changes made timely feedback to staff difficult.

Collective action (putting rapid STI test results service into operation)

The service was implemented for male patients in November 2018 and for all patients in August 2019.

Important in collective action was designing and documenting the new patient pathways, which needed to be clear but flexible to allow staff deviation from protocol to respond to individual patient situations and need (e.g. anxiety, medical history, relationship status, availability to attend clinic). Guidelines, Standard Operating Procedures (SOPs) and pathways had to be rewritten as initial implementation issues were resolved. However, detailed SOPs were not always in place prior to implementation of a new modification to a pathway, making it difficult for staff to keep up with current processes. This was due to the repeated and frequent changes to clinic processes/patient pathways to resolve initial implementation issues, and the lack of protected administrative staff time meant that when patient pathways were revised following staff feedback these could take over 6 weeks to review and be signed off by the clinical governance group. For example, the triage form asking patients to self-identify at reception whether they were symptomatic, their risk level, and if they had had sex against their will was revised three times during implementation to make it clearer

For peer review only - http://bmjopen.bmj.com/site/about/guidelines.xhtml

For peer review only

which pathway should be followed. Observation showed that they annotated a copy of the triage form to remind them of the pathways for different responses. In contrast, patients were happy with communication about the changes made (via the website, staff, consultations, on the triage form).

Although staff accepted the continual adaptations as inevitable due to the novelty of the service, it was difficult. During initial implementation, 'teething issues' were experienced, including administrative staff not knowing which patients were eligible for the service, dealing with the high volume of patients when the doors first open, and the best way to triage patients. Staff worked together to adjust and improve the new service, identifying problems and opportunities and innovating in their own practice, overseen and supported by the implementation groups, and morale was buoyed by the positive impact on patient care and the positive feedback from the research team.

The evaluation process played an important role in the success of the service by rapidly feeding back issues for resolution. The evaluation process aided communication, and researchers were able to suggest solutions to problems based on the non-participant observation. For example, researcher (EB) co-developed with the clinical team a laminated card for patients explaining the new service in response to the researcher observation that patients were given variable information by reception staff. Some of the changes to the patient pathway (Table 2 and 3) caused challenges. The responsive model meant comprehensive preparatory training and communication to all staff was challenging, and multiple methods of communication were essential. Many staff found changing ingrained behaviours difficult, particularly reducing the content and duration of consultations when they had been taught to maximise patient contact. The shorter initial appointments, with reduced medical record completion and fewer physical examinations, was a 'huge change' and source of concern and anxiety for clinicians both before and during the changes, due to perceived loss of opportunities for patient discussions about domestic violence, female genital mutilation, alcohol use, and contraception etc. which are seen as essential for a 'holistic', 'integrated' 'level 3 service'. This did improve with practice, and patients with particularly concerning issues were referred for a health adviser consultation, which was longer under the new service. Self-sampling drop-off also meant reduced clinical contact, particularly for asymptomatic, low-risk men with negative test results (health advisers only see high-risk/new MSM patients at the first visit). The walk-in clinic was therefore more demanding, as the case mix changed, seeing more symptomatic patients with complex presentations. Although reduced clinical contact with asymptomatic patients was a planned cost-saving benefit, it meant nursing assistants (running the sample drop-off sessions) collected mandatory data (GUMCAD surveillance system[40]) and answered patient clinical queries, which they were not qualified/paid/willing to do.

In the new service, chlamydia and gonorrhoea treatments were to be given based on results, not presumptively unless sexual contact with a case was within the 2-week window period and patients requested treatment[6 7]. Men with symptoms of urethritis were first tested for chlamydia/gonorrhoea and booked to return more than 4 hours later. If NAAT-positive they were treated according to British Association for Sexual Health and HIV (BASHH) chlamydia and gonorrhoea guidelines[6 7] and if negative tested for urethritis and managed according to BASHH guidelines[41] [with reassurance, including a leaflet, if negative] and told to re-attend for an early morning smear if their symptoms did not resolve. Some patients, particularly regular clinic attendees, were initially not keen on this longer wait for treatment, although this did improve. A minority of clinicians deviated from protocol and treated presumptively, especially for patients who were particularly anxious. Staff reported mixed patient understanding of only treating when results

For peer review only - http://bmjopen.bmj.com/site/about/guidelines.xhtml

were available, with detailed explanations needed, but patients were amenable once they understood.

Reflexive monitoring (appraisal of STI test results service into operation)

Contextual factors

In addition to the contextual factors described above of inadequate service funding, understaffing and, ongoing communication problems, increased use of postal testing (meaning less complex patients used postal testing and more complex patients used the walk-in clinic), and increasing use of PrEP increasing service demand. Increasing societal awareness of gender issues also influenced the service experience, with triage forms issued to male patients on arrival creating tensions around sensitively managing patients who did not identify with their sex assigned at birth (including trans and non-binary patients). This process was amended following feedback from the research team.

Success

Overall, the new service was seen as successful as it was implemented and running fairly smoothly after initial problems (Table 4). Although the process was challenging, implementation was an achievement, given the constraints on resources and staffing and lack of additional funding highlighted above. Staff were credited with being adaptable, highly motivated, hardworking, and mutually supportive. Staff job satisfaction and morale was boosted from doing something new and exciting and they felt proud about achieving implementation which contributed to enhanced teamwork and coherence. Better job satisfaction was mainly due to improvements to consultations with patients, including consultants seeing more complex patients. These boosts gave the team confidence that they could make further service improvements, demonstrated by the rapid changes made during the first wave of the COVID-19 pandemic during which staff reported being more 'change-ready'.

Although staff were initially concerned that the changes would jeopardise the quality of care, this does not appear to have been realised and patients felt very positive about staff and the ability to raise concerns and discuss issues. Staff perceived that the service was able to see more patients, and that clinicians and health advisers could spend more time and better engage with complex and higher risk patients due to more efficient processing of patients attending for routine testing . Self-testing and fewer physical examinations involving invasive sampling (urethral swab) was generally preferred by patients. Decreased time to diagnosis and treatment meant less patient anxiety while waiting for results and most patients were happy to wait up to 48 hours for treatment. Indeed, patients rated the quality of the new service highly, with some patients specifically requesting it. Staff, and some patients, were pleased to be able to treat with results, which promoted informed discussions and reduced antibiotic use, secondary complications, and onward transmission.

Suggested improvements

For many staff the most important improvements to the implementation were preparation of documentation of new processes and pathways as soon as possible and engaging and supportive communication from senior staff with all staff but particularly nursing and reception teams to improve process design iterations. This communication should use a variety of methods (especially face-to-face) including written, training sessions, on-the-job support, informal, and nominated individuals for support. Bringing teams together for training was recommended to facilitate information exchange and understanding. It was recommended that, if possible, staff needed to be better prepared for behaviour change and multiple continual adaptations. Staff also need protected

For peer review only - http://bmjopen.bmj.com/site/about/guidelines.xhtml

time for the project, and the impact on staff roles and workloads needs to be better considered. Small-scale pilots of the new service with patients, to test and refine draft processes to reduce staff stress and confusion were proposed. Other areas for improvement were: consistency in the rapidity of results and contingency planning for malfunctions (sometimes results were not available on time due to Panther machine breakdowns); more and earlier information for patients, especially on the process and timings (waiting times, results notification etc). Finally, the use of phone/video clinics, which were implemented during physical distancing requirements of COVID-19, may have benefits elsewhere.

The Supplementary File summarises service considerations for implementing a rapid STI service, and relevant teams/job roles.

Discussion

Principal findings

The first UK rapid NAAT testing integrated SSHS for chlamydia and gonorrhoea was successfully implemented despite funding and staff shortages. Inevitable initial challenges were resolved and, overall, it was well received. Staff were enthusiastic about it and understood the benefits, although some were concerned about reduced patient contact. The use of NPT allowed for examination of issues with both the design of the rapid service and its implementation. Cognitive participation difficulties included engaging all staff and changing ingrained behaviours (resulting from extensive training and audit), especially for administrative and nursing staff, although staff did support each other and work together. Some patients had concerns about waiting for treatment, but most accepted sample drop-off and returning for a follow-up appointment. Reflexive monitoring revealed perceived benefits including reduced patient anxiety, seeing more patients, and boosting staff job satisfaction. Infection-specific treatment based on test results was crucial, enabling informed consultations and improving antimicrobial stewardship. Suggestions for this and other future services included: documenting new pathways and processes early and comprehensively disseminating to staff; involving all staff in planning, design and implementation; protecting staff time for meetings and actions; considering pilots with a small group of staff/patients before sharing more widely or writing guidelines; cross-discipline training; varied methods of, sensitive and supportive communication; considering staff role impact, and ensuring staffing to cover changes.

Relation to other studies

Evaluating the real-life implementation of a novel rapid results service confirms previous hypothetical/simulated studies where patients were happy with the service and willing to wait for results before treatment[14-16 25]. Whereas previous research has found that the patients found the hypothetical scenario of waiting up to 40 minutes for test results acceptable[25 26], our findings demonstrate that patients were happy to wait up to 48 hours for treatment based on results. Willingness to wait has been found to be dependent on self-assessed infection risk and anxiety about their infection status [25]. Our findings demonstrate that the rapid service can lead to less patient anxiety due to shorter time waiting for results and therefore should target patients concerned they are infected. Although asymptomatic patients are encouraged to use on-line postal services, some patients may wish to attend in-person clinics[13 42]. The previously anticipated[12 18] benefits of treating with results and improving antimicrobial stewardship are highly valued by staff and patients in our evaluation. Modelling studies have demonstrated that rapid testing can enable faster treatment, reduces infectious periods, and leads to fewer transmissions, partner attendances and clinic costs[43 44]. Rapid diagnostics and treatment can increase the proportion of individuals

For peer review only - http://bmjopen.bmj.com/site/about/guidelines.xhtml

receiving timely treatment and decrease community prevalence of STIs[45 46] and recently has been seen as a key factor contributing to the reducing new HIV infections in London and ensuring those with HV receive fast and optimal care[47]. Our findings also confirm reductions in patient anxiety[12 17 18] and improved testing uptake[19-21] are likely, as well as freeing up clinician time, greater clinician confidence, and efficiencies allowing capacity to be utilised elsewhere[12].

The challenges of communicating with and engaging all staff, especially those 'on the ground', and the need for dedicated time for training and implementation[48] are key in healthcare quality improvement[48]. Teething issues experienced in this service – documentation of new pathways, impact on staff roles – and the challenges of changing ingrained behaviour – are common in implementation of a major service change and emphasise the importance of staff training and communication of the reason and implications for change[49].

Our findings demonstrate that successfully implementing a beneficial service change can boost staff job satisfaction and morale. Previous research has found improvements in staff satisfaction following successful sexual healthcare innovation[49]. This finding suggests the implementation realised benefits for staff - previously highlighted as influencing acceptance of change in NHS service improvement programmes[50] – and aligned with professionals values and intrinsic motivation to provide quality and effective care[48].

Implications

This study shows that a rapid NAAT-testing integrated SSHS for chlamydia and gonorrhoea can be implemented in a constrained NHS system, and is acceptable to patients, with benefits for staff, patients and public health, including reduced patient anxiety. The perceived efficiency (to be clarified in a separate quantitative evaluation) is crucial given the financial and staffing pressures on UK sexual health services[51]. Similarly, the pride of staff in their service, and enhanced staff satisfaction are important in boosting staff morale and is likely to further enhance the provision of high-quality patient care when such a service is introduced.

AMR is a major concern for gonorrhoea, and a priority worldwide[52] and in England[53]. Rapid STI services could play a vital role in reducing unnecessary antibiotic prescribing by providing test results during/soon after consultations, allowing informed clinician choices. When the technology becomes available, the addition of POCTs to detect ciprofloxacin-sensitive gonorrhoea will dramatically reduce reliance on ceftriaxone and selection pressure for AMR[54 55].

Our implementation recommendations for future services echo those from the Health Foundation, such as sensitive leadership oriented towards inclusion, agreeing roles and responsibilities at the outset and 'bringing everyone along with you'[48], as well as early documentation, piloting pathways, varying communication methods and adequate staffing. The willingness of symptomatic male patients to wait for treatment can inform development of new care pathways using POCTs[12 56], although results are limited to a single service and male patients.

Strengths and limitations

Project strengths include: integration of findings from multiple qualitative methods generating rich insights, a multidisciplinary team including clinical academics; a strong trusting relationship between research team and clinical staff due to existing relationships and research team flexibility and responsiveness; regular feedback from researchers to clinicians using a 'trial, assess, adapt' strategy. EB and JK came to the observations as experienced researchers and with good knowledge of the plans for the service changes and reasons for them. The researchers were surprised at how quickly it was possible to provide information and feedback to the implementation team which they clearly

For peer review only - http://bmjopen.bmj.com/site/about/guidelines.xhtml

valued highly and rapidly implemented changes based on it. The researchers could move freely between different physical areas of the clinic and stages of the process in a way which clinic staff were not free to do, which provided early insights. Due to the study design and relationships, these insights could be discussed promptly with relevant staff - and so sense checked, and action taken in response if appropriate (changes to clinic processes; further data collection etc.). The rapid, supportive, evidence-based feedback which the researchers could provide seemed to quickly build the confidence of the key implementation staff in the research process. The researchers appeared to be quickly accepted as trusted team members, with the capacity to help with the work at hand (rather than creating 'research burden').

Limitations include an all-male patient sample as the service was initially only for males, and when implemented for females, few were eligible and evaluation was hampered by the COVID-19 pandemic. We aimed to include patients with positive STI results but most (although symptomatic) were negative, limiting evaluation of follow-up appointments. COVID-19 meant fewer final batch interviews. As the rapid STI result technology develops, continued implementation evaluation is important[56], capturing, the wide-ranging impact on services, staff and patients. Evaluation for female patients is needed, given the challenges around contraception and STIs/symptoms.

Conclusion

As the first UK SSHS to implement rapid NAAT testing for chlamydia and gonorrhoea within an integrated service, this project faced the challenge of innovating to save time/money and improve patient experience in a constrained environment, particularly lack of funding and understaffing. Inevitable challenges – mainly related to the impact on patient pathways - were resolved and, overall, it was a success. Perceived benefits included reduced patient anxiety, seeing more patients, treating with results, reduced antibiotics use and boosting staff job satisfaction. Learning for other services considering implementing something similar includes more inclusive staff engagement, sensitive communication, better documentation of changes, dealing with constant adaptations, and consideration of the impact on staff and their roles.

Figure 1. Overview of rapid pathway service redesign (MSM = Men who have sex with men; NAAT = Nucleic Acid Amplification Test; GC = Gonorrhoea Culture, appt = appointment)

For peer review only - http://bmjopen.bmj.com/site/about/guidelines.xhtml

Acknowledgements

We would like to thank all study participants.

Funding statement

Funded by the National Institute for Health Research (NIHR) Applied Research Collaboration (ARC) West at University Hospitals Bristol and Weston NHS Foundation Trust and NIHR Health Protection Research Unit (HPRU) in Behavioural Science and Evaluation at University of Bristol in partnership with Public Health England (PHE). Award number P315. The views expressed are those of the authors and not necessarily those of the NIHR, the Department of Health and Social Care or PHE.

Competing interests statement

AL – no competing interests
EB - no competing interests
JK - no competing interests
PH - no competing interests
MC - no competing interests
MC - no competing interests
JS - no competing interests
JT - no competing interests
PM - no competing interests
JH - no competing interests

Author contributions

JH, PH, EB, JK were responsible for the study design. JH and EB were responsible for study management and coordination. EB, AL, JK and JH led data collection and analysis. MC and MC co-led the development and implementation of the new service model. PM, JS and JT supported implementation and accreditation of Point of Care testing. MC, MC, JS, JT, PM supported the study design and interpretation of interview findings. All authors read, commented on and approved the final manuscript.

Data Sharing Statement

Data are available on request from corresponding author.

For peer review only - http://bmjopen.bmj.com/site/about/guidelines.xhtml

References

1. England PH. Sexually transmitted infections and screening for chlamydia in England, 2019. 2019
2. Public Health England. Sexually transmitted infections (STIs): annual data tables. London: Public Health England, 2018.
3. Mohammed H, Sile B, Furegato M, et al. Poor adherence to gonorrhoea treatment guidelines in general practice in England. *The British journal of general practice : the journal of the Royal College of General Practitioners* 2016;66(648):352. doi: 10.3399/bjgp16x686077
4. Eyre DW, Town K, Street T, et al. Detection in the United Kingdom of the Neisseria gonorrhoeae FC428 clone, with ceftriaxone resistance and intermediate resistance to azithromycin, October to December 2018. *Euro Surveill* 2019;24(10):1900147. doi: 10.2807/1560-7917.ES.2019.24.10.1900147
5. White C. Sexual health services on the brink. *BMJ* 2017;359 doi: 10.1136/bmj.j5395
6. Fifer H, Saunders J, Soni S, et al. 2018 UK national guideline for the management of infection with Neisseria gonorrhoeae. Updated 2020. *International journal of STD & AIDS* 2020;31(1):4-15. doi: 10.1177/0956462419886775 [published Online First: 2019/12/25]
7. Nwokolo NC, Dragovic B, Patel S, et al. 2015 UK national guideline for the management of infection with Chlamydia trachomatis. Updated 2018. *International journal of STD & AIDS* 2018;27(4):251-67. doi: 10.1177/0956462415615443 [published Online First: 2015/11/06]
8. Price MJ, Ades AE, Soldan K, et al. The natural history of Chlamydia trachomatis infection in women: a multi-parameter evidence synthesis. *Health technology assessment (Winchester, England)* 2016;20(22):1-250. doi: 10.3310/hta20220 [published Online First: 2016/03/24]
9. Harding-Esch EM, Fuller SS, Chow SC, et al. Diagnostic accuracy of a prototype rapid chlamydia and gonorrhoea recombinase polymerase amplification assay: a multicentre cross-sectional preclinical evaluation. *Clinical microbiology and infection : the official publication of the European Society of Clinical Microbiology and Infectious Diseases* 2019;25(3):380.e1-80.e7. doi: 10.1016/j.cmi.2018.06.003 [published Online First: 2018/06/16]
10. Van Der Pol B. Making the Most of Point-of-care Testing for Sexually Transmitted Diseases. *Clinical Infectious Diseases* 2019 doi: 10.1093/cid/ciz523
11. Rönn MM, Menzies NA, Gift TL, et al. Potential for Point-of-Care Tests to Reduce Chlamydia-associated Burden in the United States: A Mathematical Modeling Analysis. *Clinical Infectious Diseases* 2019 doi: 10.1093/cid/ciz519
12. Adams EJ, Ehrlich A, Turner KME, et al. Mapping patient pathways and estimating resource use for point of care versus standard testing and treatment of chlamydia and gonorrhoea in genitourinary medicine clinics in the UK. *BMJ Open* 2014;4(7) doi: 10.1136/bmjopen-2014-005322
13. Whitlock GG, Gibbons DC, Longford N, et al. Rapid testing and treatment for sexually transmitted infections improve patient care and yield public health benefits. *International journal of STD & AIDS* 2018;29(5):14. doi: 10.1177/0956462417736431 [published Online First: 2017/10/24]
14. Llewellyn CD, Sakal C, Lagarde M, et al. Testing for sexually transmitted infections among students: a discrete choice experiment of service preferences. *BMJ Open* 2013;3(10) doi: 10.1136/bmjopen-2013-003240
15. Chislett L, Clarke J. Which elements of a novel self-directed rapid asymptomatic sexually transmitted infection screening service are most important to users? . Sexually Transmitted Infections, 2015:A1–A258.
16. Mahilum-Tapay L, Laitila V, Wawrzyniak JJ, et al. New point of care Chlamydia Rapid Test--bridging the gap between diagnosis and treatment: performance evaluation study. *BMJ (Clinical research ed)* 2007;335(7631):1190-94. doi: 10.1136/bmj.39402.463854.AE [published Online First: 11/30]

For peer review only - http://bmjopen.bmj.com/site/about/guidelines.xhtml

17. Llewellyn C, Pollard A, Miners A, et al. Understanding patient choices for attending sexually transmitted infection testing services: a qualitative study. *Sex Transm Infect* 2012;88(7):504-9. doi: 10.1136/sextrans-2011-050344 [published Online First: 2012/05/26]
18. Turner KME, Round J, Horner P, et al. An early evaluation of clinical and economic costs and benefits of implementing point of care NAAT tests for Chlamydia trachomatis and Neisseria gonorrhoea in genitourinary medicine clinics in England. *Sexually Transmitted Infections* 2013 doi: 10.1136/sextrans-2013-051147
19. Natoli L, Guy RJ, Shephard M, et al. Public health implications of molecular point-of-care testing for chlamydia and gonorrhoea in remote primary care services in Australia: a qualitative study. *BMJ Open* 2015;5(4) doi: 10.1136/bmjopen-2014-006922
20. Rompalo AM, Hsieh Y-H, Hogan T, et al. Point-of-care tests for sexually transmissible infections: what do 'end users' want? *Sexual health* 2013;10(6):541-45. doi: 10.1071/SH13047
21. Horwood J, Ingle SM, Burton D, et al. Sexual health risks, service use, and views of rapid point-of-care testing among men who have sex with men attending saunas: a cross-sectional survey. *International journal of STD & AIDS* 2016;27(4):273-80. doi: 10.1177/0956462415580504 [published Online First: 2015/04/25]
22. Lorenc T, Marrero-Guillamón I, Aggleton P, et al. Promoting the uptake of HIV testing among men who have sex with men: systematic review of effectiveness and cost-effectiveness. *Sexually Transmitted Infections* 2011;87(4):272-78. doi: 10.1136/sti.2010.048280
23. Read TRH, Hocking JS, Bradshaw CS, et al. Provision of rapid HIV tests within a health service and frequency of HIV testing among men who have sex with men: randomised controlled trial. *BMJ : British Medical Journal* 2013;347 doi: 10.1136/bmj.f5086
24. Public Health England. Strategic framework to promote the health and wellbeing of gay, bisexual and other men who have sex with men 2014a [Available from: https://www.gov.uk/government/uploads/system/uploads/attachment_data/file/313692/Strategic_Framework_to_promote_the_health_and_wellbeing_of_MSM_FINAL_DRAFT_For_comment.pdf.
25. Fuller SS, Pacho A, Broad CE, et al. "It's not a time spent issue, it's a 'what have you spent your time doing?' issue. . ." A qualitative study of UK patient opinions and expectations for implementation of Point of Care Tests for sexually transmitted infections and antimicrobial resistance. *PloS one* 2019;14(4):16. doi: 10.1371/journal.pone.0215380
26. Widdice L, Hsieh YH, Silver B, et al. Performance of the Atlas Genetics Rapid Test for Chlamydia trachomatis and women's attitudes toward point-of-care testing. *Sexually Transmitted Diseases* 2018;45(11):5. doi: 10.1097/OLQ.0000000000000865
27. Hsieh YH, Hogan MT, Barnes M, et al. Perceptions of an ideal point-of-care test for sexually transmitted infections--a qualitative study of focus group discussions with medical providers. *PloS one* 2010;5(11):e14144. doi: 10.1371/journal.pone.0014144 [published Online First: 2010/12/15]
28. Hsieh Y-H, Gaydos CA, Hogan MT, et al. What Qualities Are Most Important to Making a Point of Care Test Desirable for Clinicians and Others Offering Sexually Transmitted Infection Testing? *PloS one* 2011;6(4):e19263. doi: 10.1371/journal.pone.0019263
29. Hall C, Carter N, Crofts M, et al. P. P155 "time in clinic" survey to evaluate the potential for use of online registration. . *Sexually Transmitted Infections* 2015;91:A66-A67.
30. Huntington SE, Burns RM, Harding-Esch E, et al. Modelling-based evaluation of the costs, benefits and cost-effectiveness of multipathogen point-of-care tests for sexually transmitted infections in symptomatic genitourinary medicine clinic attendees. *BMJ Open* 2018;8
31. Natoli L, Guy RJ, Shephard M, et al. "I Do Feel Like a Scientist at Times": A Qualitative Study of the Acceptability of Molecular Point-Of-Care Testing for Chlamydia and Gonorrhoea to Primary Care Professionals in a Remote High STI Burden Setting. *PloS one* 2016;10(12):e0145993. doi: 10.1371/journal.pone.0145993

For peer review only - http://bmjopen.bmj.com/site/about/guidelines.xhtml

32. Turner KME, Looker KJ, Syred J, et al. Online testing for sexually transmitted infections: A whole systems approach to predicting value. *PloS one* 2019;14(2):e0212420. doi: 10.1371/journal.pone.0212420 [published Online First: 2019/02/23]
33. Higginbottom GM, Pillay JJ, Boadu NY. Guidance on Performing Focused Ethnographies with an Emphasis on Healthcare Research *The Qualitative Report* 2013;18(9):1-6.
34. Murray E, Treweek S, Pope C, et al. Normalisation process theory: a framework for developing, evaluating and implementing complex interventions. *BMC medicine* 2010;8:63. doi: 10.1186/1741-7015-8-63 [published Online First: 2010/10/22]
35. MacGregor L, Speare N, Nicholls J, et al. The role of the evolving trends in sexual behaviours and clinic attendance patterns on increasing diagnoses of STIs: a perspective from Bristol, UK. *Sexually Transmitted Infections* 2020;Accepted for Publication
36. Malterud K, Siersma VD, Guassora AD. Sample Size in Qualitative Interview Studies:Guided by Information Power. *Qualitative Health Research* 2016;26(13):1753-60. doi: 10.1177/1049732315617444
37. Reeves S, Kuper A, Hodges BD. Qualitative research methodologies: ethnography. *BMJ* 2008;337 doi: 10.1136/bmj.a1020
38. Emerson R, Fretz R, Shaw L. Writing ethnographic fieldnotes. Chicago and London: University of Chicago Press 1995.
39. Braun V, Clarke V. One size fits all? What counts as quality practice in (reflexive) thematic analysis? *Qualitative Research in Psychology* 2020:1-25. doi: 10.1080/14780887.2020.1769238
40. Public Health England. Guidance on the collection and reporting of data for GUMCAD, the surveillance system for sexually transmitted infections (STIs) in England. 2019 [Available from: https://www.gov.uk/guidance/gumcad-sti-surveillance-system
41. Horner P, Blee K, O'Mahony C, et al. 2015 UK National Guideline on the management of non-gonococcal urethritis. *International journal of STD & AIDS* 2016;27(2):85-96. doi: 10.1177/0956462415586675 [published Online First: 2015/05/24]
42. Turner KME, Zienkiewicz AK, Syred J, et al. Web-Based Activity Within a Sexual Health Economy: Observational Study. *J Med Internet Res* 2018;20(3):e74. doi: 10.2196/jmir.8101
43. Mohiuddin S, Gardiner R, Crofts M, et al. Modelling patient flows and resource use within a sexual health clinic through discrete event simulation to inform service redesign. *BMJ Open* 2020;10(7):e037084. doi: 10.1136/bmjopen-2020-037084 [published Online First: 2020/07/10]
44. Whitlock GG, Gibbons DC, Longford N, et al. Rapid testing and treatment for sexually transmitted infections improve patient care and yield public health benefits. *International journal of STD & AIDS* 2018;29(5):474-82. doi: 10.1177/0956462417736431 [published Online First: 2017/10/24]
45. Keizur EM, Goldbeck C, Vavala G, et al. Safety and Effectiveness of Same-Day Chlamydia trachomatis and Neisseria gonorrhoeae Screening and Treatment Among Gay, Bisexual, Transgender, and Homeless Youth in Los Angeles, California, and New Orleans, Louisiana. *Sex Transm Dis* 2020;47(1):19-23. doi: 10.1097/olq.0000000000001088 [published Online First: 2019/11/07]
46. Natoli L, Guy RJ, Shephard M, et al. Public health implications of molecular point-of-care testing for chlamydia and gonorrhoea in remote primary care services in Australia: a qualitative study. *BMJ Open* 2015;5(4):e006922. doi: 10.1136/bmjopen-2014-006922 [published Online First: 2015/04/30]
47. Girometti N, Delpech V, McCormack S, et al. The success of HIV combination prevention: The Dean Street model. *HIV medicine* 2021 doi: 10.1111/hiv.13149 [published Online First: 2021/07/30]

For peer review only - http://bmjopen.bmj.com/site/about/guidelines.xhtml

48. Dixon-Woods M, McNicol S, Martin G. Ten challenges in improving quality in healthcare: lessons from the Health Foundation's programme evaluations and relevant literature. *BMJ Quality & Safety* 2012;21(10):876-84. doi: 10.1136/bmjqs-2011-000760
49. Boog K, Heanue J, Kumar V. Implementing integrated sexual and reproductive healthcare in a large sexual health service in England: challenges and opportunities for the provider. *BMJ Sexual & Reproductive Health* 2019;45(2):164-67. doi: 10.1136/bmjsrh-2018-200090
50. Gollop R, Whitby E, Buchanan D, et al. Influencing sceptical staff to become supporters of service improvement: a qualitative study of doctors’ and managers’ views. *Quality and Safety in Health Care* 2004;13(2):108-14. doi: 10.1136/qshc.2003.007450
51. The Kings Fund. Understanding NHS financial pressures: How are they affecting patient care?, 2017.
52. World Health Organisation. Emergence of multi-drug-resistant Neisseria gonorrhoeae—threat of global rise in untreatable sexually transmitted infections, 2012.
53. Public Health England. PHE infectious diseases strategy 2020 to 2025, 2019.
54. Town K, Harris S, Sánchez-Busó L, et al. Genomic and Phenotypic Variability in Neisseria gonorrhoeae Antimicrobial Susceptibility, England. *Emerg Infect Dis* 2020;26(3):505-15. doi: 10.3201/eid2603.190732
55. Zienkiewicz AK, Verschueren van Rees N, Homer M, et al. Agent-based modelling study of antimicrobial-resistant Neisseria gonorrhoeae transmission in men who have sex with men: towards individualised diagnosis and treatment. *Sex Health* 2019;16(5):514-22. doi: 10.1071/sh18235 [published Online First: 2019/09/03]
56. Toskin I, Govender V, Blondeel K, et al. Call to action for health systems integration of point-of-care testing to mitigate the transmission and burden of sexually transmitted infections. *Sex Transm Infect* 2020;96(5):342-47. doi: 10.1136/sextrans-2019-054358 [published Online First: 2020/04/04]

For peer review only

For peer review only - http://bmjopen.bmj.com/site/about/guidelines.xhtml

Table 1: Quotes on Coherence and Cognitive Participation	
**Coherence**	**Cognitive participation**
**Staff enthusiasm and concerns**<br>It all sounded quite exciting and I was quite, not excited, but it was like, this is really good, the first one in the UK and, you know, this will be excellent, so I was quite open minded about it... lowering the use of antibiotics and fewer invasive procedures for women. (Nurse 17, T3)<br>It's an exciting opportunity, um, but also there's a bit of, um, er, you know, nerves I suppose about how it will actually be, actually run from day one, really, and how it would go (Public Health 14, T2)	**Disengaged staff**<br>It was very much the higher-up staff that kind of organised it all and they're not really the ones that are going to be doing the actual work, so I think it's really important to include clinical staff of all levels, kind of when it's getting near to it and you know, really explain, and have their opinions and thoughts on, you know, how it's gonna work (Nurse 16, T2)
**Reduced patient anxiety**<br>Results on the same day would be amazing, yeah, no doubt about that yeah, because there's always quite an – well, for me, it's like an anxious wait otherwise. Yeah, that sense of not knowing and actually "how do I manage my sex life in case anything comes back positive?" Yeah, quick results can definitely make a big difference (Mike, didn't use rapid STI service)<br>The thing that would encourage me to test more regularly, [is] if the whole thing [time spent in clinic] could be done in a shorter time slot for me (Ben, used rapid STI service)	**Barriers to engagement**<br>[Clinic managers need to] canvass people's feelings about it because I don't know that we do that very well. I think we just crack on. We don't say 'how was it for you that first week did you cope? Did you keep your head above water?' (Nurse 10, T1)

For peer review only

For peer review only - http://bmjopen.bmj.com/site/about/guidelines.xhtml

Avoiding inappropriate antibiotics prescriptions	Inadequate preparation
With antibiotics I'm really – they really mess with my stomach. I really feel really sick whenever I do take them. So, I'm just pro not taking them for that reason alone. Yeah, like – I try to think about the bigger picture of the world and stuff…but I think about my own stomach more than the wider world. So, yeah, I'd – I'm always pleased to like not have to do any unnecessary drugs (Andy, results by text)<br>I just think I'd hate to kind of like take something that I didn't have and then if I ever got it again it doesn't work - so it's kind of like half society view half personal view (Harry, follow-up appointment)	We were told on the Wednesday that it was supposed to be starting on the Monday and we were like all a bit shocked thinking 'well hang on a minute what about the training? We'll look really, really stupid in front of patients' (Administrative staff 6, T1)<br>There was always talk about what it [Panther] could do, never talk about how it's going to function. Even at the last minute, the week before it was meant to start [Lead Consultant] came to me and goes, 'This is what I think the pathway is. Can you make notes on it?' Nobody was really clear about what happened. [Project manager] had sent a PowerPoint around and it was embedded in a way that… some staff who aren't maybe familiar with how embedded links and things work [- couldn't open it]. When the meeting came around, they said, 'Has everyone read the email about the Pathways?' half of the people went, 'What email?' …, it just wasn't presented to us in a clearest way. (Health Adviser 1, T2)

For peer review only

Table 2: Changes along the (walk-in) male patient pathway

	Before rapid STI service	Rapid STI service
**Registering at reception**	All walk-in patients allocated an appointment in order of queue – may have to come back later that day.	Receptionist triages each patient to appropriate pathway referring to guide/pathway
		Patients not eligible for rapid STI service are given an appointment for later that day and continue on old pathway
		Rapid STI service-eligible patients wait after registering to be called up.
	Triage form used to register patients.	Triage form amended to be gender neutral and to make categories clearer.
**Seeing a clinician/ health adviser**	At first appointment	First appointment very brief– reduced history taking. Unless uncomplicated vaginal discharge.
	MSM and all patients needing partner notification/ risk reduction/safer sex advice see health adviser	High-risk patients (MSM or new to service) see health adviser at initial appointment.
		Symptomatic men see doctor/trained nurse only at follow-up not drop-off, usually on same day.
**Providing samples**	Urine and swabs: taken at time of consultation. Self-taken if asymptomatic. If symptomatic: clinician taken swab for microscopy (to detect NGU and gonorrhoea) and gonorrhoea culture; NAAT self-taken	Self-sample drop-off - Urine and swabs NAAT self-sampled in toilets, putting samples through a hatch to the laboratory. Instructions are on posters in the toilet and from nursing staff/NAs. Gonorrhoea culture taken by clinician on return only if NAAT positive. Swab for microscopy to detect NGU if NAAT-negative (see text above).
	Blood samples taken by doctor/nurse/nursing assistant (NA)	Blood samples taken by doctor/nurse/NA
**Tests results**	All STI test results in 2 -3 weeks	Chlamydia and gonorrhoea processed on Panther - results within 48 hours. Others still 2-3 weeks
	If negative, text sent	Results by text if asymptomatic and negative.
	If positive, HA phones patient to discuss result and arrange treatment (unless already received presumptive treatment).	Results given at follow up appointment with clinician if symptomatic or asymptomatic positive.
**Treatment**	Treat presumptively	Wait for results before treating
	Treat at first (only) appointment	Treat at follow-up appointment
**Notified partners**	Treated immediately	Only treat on positive results (if sexual contact was >2 weeks; otherwise treat immediately)

For peer review only

For peer review only - http://bmjopen.bmj.com/site/about/guidelines.xhtml

Table 3: Collective action quotes
**Designing and documenting new processes**<br>We need to be really clear about what we're doing when they drop-off [patients drop-off samples], what we're doing when they come back, what are we going to do about contraception, which questions are okay to leave out of the proforma. ..for consultants because we have a lot of experience and because we're used to making decisions then I think we can [unclear] a bit and we can be flexible and can you know think about the individual patient. But for nurses who work a lot to PGD's [guidelines] and like to have clear guidance. And some of the juniors as well, who will be quite new - you know they've just been changed. Because it's going to be bewildering and chaotic you know it's doesn't feel good when there's chaos on the shop floor. (Doctor 3, T1)
**Flexibility in pathways**<br>It's a case by case situation and it does help to have helpful medical staff that have been willing to make an exception. (Administrative staff 18, T2)<br>I think the health advisers also are more able to …know the guidelines but in some situations know that you have to approach things differently, … for me personally if I was seeing someone and they kind of said 'actually I have got this dis[ease]' - you know, real clear symptoms, you know, 'and I'm really fed up with it', I'd be more inclined to say 'okay then let's get you treated…I think you can have your general thing of saying to someone 'look you know come back in the afternoon' but if you've got someone who's kind of 'actually no but I've had these symptoms for two days I've really had enough of it'. (Health Adviser 13, implementation group member, T2)
**Guidelines**<br>It's a work in progress but the problem is as the pathway evolves then the guideline will change again…because this is so rapidly moving actually, I don't think I really want to do a guideline. So it's kind of hard to have a guideline anyway but we need some kind of guidance. (Doctor 3, T1)
**Teething issues**<br>It was chaos, the first few weeks were chaos. Reception didn't know what they were doing… there was hundreds of patients around the reception, we didn't know what we were doing, so yeah, it was chaos, but it has slowly got better. (Nurse 17, T2)<br>The waiting area fills up and people are filling out the Panther triage forms on windowsills. After a while [clinic coordinator] tells receptionists on Panther desk that he had given out 16 forms. Once they get to 10 people booked for Panther returner pathway they need to go check with the laboratory regarding further capacity. (Observation notes, Reception area)<br><br>Two reception staff were unsure whether one person should be panther/same day or walk-in due to the information provided on the form. Staff consulted with person entering data on computer. They checked whether person was returning for results/treatment. They explained to the patient that a new system is in place, so they want to make sure they do the best for him. (Observation notes, Reception area)
**Understaffing**<br>It's been very stressful for staff and I think it has been an enormous amount of work for the implementation group, that I think in the private industry you'd be given huge amounts of time, whereas we virtually squeezed it in amongst everything else we've done, but that's just the NHS. (Doctor 11, T2)<br>Administrative staff / reception team has three staff vacancies, and today there are two members of clinic staff off sick – one clinician, and one Administrative staff (clinic coordinator). The clinician would have been doing sample drop-off, and walk-in, so have had to reduce slots for both until they get confirmation of clinical capacity from clinicians when they arrive. (Observation notes, Reception area)
**Changing ingrained behaviours**

For peer review only - http://bmjopen.bmj.com/site/about/guidelines.xhtml

It's been quite hard on staff and obviously there's a lot of – you know, if you've been doing something the same way for 10/20/maybe 30 years, that's quite a massive change for people. (Nurse 21, T3)
**Changes to clinician contact**<br>We've actually ended up seeing a lot more complicated or complex patients, at least that's how it feels. The easy patients get siphoned off quite quickly and that means that more patients [can be seen], especially the complex patients, which the nurses are less able to deal with and require a lot more consultant supervision. I think there has been a general feeling in the department that the consultant cover job is busier than it ever was before. (Doctor 11, T2)
**Challenges of changes at reception**<br>[Receptionists] were worried that they were looking like they didn't know what they were doing, because it was new and they weren't quite sure. So I think it took a, it was a lot to ask for them all really because it was a big change, but it is just that keep reminding everybody that actually, in the long term, you will get it, and it's much better for other patients once it's in place. (Administrative staff 12, implementation group member, T2)<br>Reception staff on male desk refresh the panther decision pathway together using A4 sheet. Female staff member commented that she always has to double check the process. Reception staff discussed male staff member's confusion about eligibility for Panther. (Observation notes, Reception area)
**Concern about shorter consultations**<br>It was a huge change, because, we, it is quite a detailed consultation. We have been told time and time again that 'oh you need to ask patients about domestic violence, ask the women about female genital mutilation, you need to do this'. Then all of a sudden, they are saying, 'no, don't ask any of these things', it's like aargh! (Nurse 17, T2)<br>It does sometimes feel if I'm absolutely honest a little bit less than a level three service, you know, people are just coming in and dropping off a sample. I know that's possibly better use of our time, but it seems a little bit spurious to call it level three. (Nurse 10, T1)
**Perceived patient views on waiting for treatment**<br>I think the major anxiety that patients have is around not being treated immediately and not being treated necessarily as a contact of infection and anxiety around that. I often find that with a bit of educating that that is overcome and my major impression is that patients really appreciate it. (Doctor 11, T2)<br>I haven't had anybody who's been absolutely, you know, anti about it but there have been a couple of people who I've thought 'I'm going to treat you mate, I'm not going to wait on results' do you know what I mean? ... they're anxious, they've maybe got another partner, a regular partner, who they don't want to infect, which, you know, I can see the reasoning behind that. But I think, you know, once that kind of idea has got out amongst our regular clientele I think it will be a lot easier. (Nurse 10, T1)<br>Four young men approach the door together. [Name] lets them know that he has just 2 forms/slots left at present, and suggests that he gives these to them on the basis of who came through the door first – these two seem pleased, and head in with their forms. He asks the other two to wait here for a minute and they seem OK with this. He tells them there is a new service which means they can give people results/treatment faster, and this is why things are different. He asks them to give him a yes/no answer as to whether they have any symptoms. When they say no he says that it is probably not worth them waiting as they are unlikely to be seen today, and that they could come back another morning for when the doors first open. They seem to find this acceptable. (Observation notes, front door)
**Benefit of evaluation process**<br>Staff member commented that it was helpful to have an outside voice (research team) feeding back, because sometimes when you are within the structure you can be shouting stuff and nobody hears you. (Observation notes)

For peer review only - http://bmjopen.bmj.com/site/about/guidelines.xhtml

Table 4: Reflexive monitoring quotes
**Success**
When you speak at the national [sexual health] meetings, people, it's a bit of a no-brainer, what we're supposed to do, and people are amazed that we've been able to introduce it [rapid STI service] cost-neutrally. Because when you look at the point-of-care systems which other people are researching, it's… more expensive, so we've adopted an innovative approach. (Doctor 19, T3)
**Quality of care**
The person I saw was really brilliant, like, yeah. I felt really comfortable… I really felt like I could ask anything I want and felt sort of safe (Andy, results by text1)
**Benefits**
It was the immediacy and the kind of reassurance that … if something was positive that you would be able to treat it straightaway. (Harry, follow-up appointment)
They're [patients] very happy. I mean, who wouldn't be? You find out the same day that you have got chlamydia and you can start your treatment. I mean that is brilliant. (Nurse 17, T3)
Dr 11: One of the advantages that we hoped would come out of introducing Panther would be that we would attract more high-risk people, because it would be seen as an attractive place to come and test, and also that it would free up staff time so that we could spend more time with risk reduction etc.
Interviewer: Do you think that is happening? Or is it not there yet?
Dr 11: I think it has started to happen, I don't think that's only down to Panther, I think that's down to some other stuff like PrEP and things like that as well. I feel like the cohort of patients that we see is increasingly complex. (Doctor 11, T2)
I think the most positive things are seeing your symptomatic patients with knowing what is going on with them. You know what infection they have, you know what treatment they require or if they don't have anything you can then take the time to discuss that. (Doctor 11, T2)
I think it's [rapid service implementation] made the staff more able to deal with change [to telephone clinics], because they had undergone experience of change with Panther pathways over the past 12-18 months…. Yes, it probably made it smoother and more efficient. (Doctor 22,T4)
**Suggested improvements**
The main issues that have arisen have been when the [Panther] machine fails and that can be pretty catastrophic (laughs), just because you have booked slots and patients come back and you don't, you can't even tell them whether they have chlamydia or gonorrhoea and they've, kind of, come with that expectation. (Doctor 20, T3)
Interviewer: If you had an imaginary clinic who were going to set out on this path, what would your advice be to them overall? With the knowledge that you've now gathered from your experience, is there a way that you could help them?
Dr 11: I think preparation, preparing all of the documents that support your staff on a day-to-day basis, clarifying your communication pathways, giving your lead clinicians adequate time in their work plans to do all of that. I would definitely support it, I think it's definitely been a major benefit. (Doctor 11, T2)
It has been a big change for all staff working, and it's difficult to know whether there was any way of realising some of the things we hadn't realised. I don't think we could have done. I think they were literally just things of implementation that have caused some additional tweaks required - and that in itself has been stressful because it's been the realisation of what are we doing in this scenario and not being quite prepared for it. (Administrative staff 12, T2)

For peer review only

The thing that I found most interesting is the communication difficulties in amongst the staff and how difficult that has been, having an implementation group that I think represents most of the groups that it's impacted upon and the difficulty that the messages just have not got to the clinic floor, and that's an on-going issue. (Doctor 11, T2)

For peer review only

For peer review only - http://bmjopen.bmj.com/site/about/guidelines.xhtml

Pathway stage	Usual care pathway	Rapid pathway
**Reception**	- Walk-in patients' queue as clinic opens and appt are allocated on first-come-first-served basis	Patients are triaged to eligible for rapid STI service or not eligible.<br>• Eligible – Registered<br>• Non-eligible - continue along old pathway
**Clinical review**	Appt with clinician, full history taken.	Brief history taken at initial appt<br>MSM referred to Health Adviser<br>If follow up appt needed then full history taken at that time
**Sample testing**	Asymptomatic: Self-taken samples<br>Symptomatic: Clinician-taken samples for NAAT, GC culture and microscopy (as appropriate)	Self-taken NAAT testing **only** at initial appt<br>GC cultures taken **only** if GC positive on NAAT<br>Samples taken for microscopy only if NAAT negative and symptomatic
**Test results**	All test results available after approximately 10-14 days	Symptomatic: results available within the same day (triggering same/next day follow-up appt)<br>Asymptomatic: within 48 hours
**Treatment**	Patients treated syndromically prior to NAAT results.<br>Patient return for infection-specific treatment when test results are known (10-14 days)	Infection-specific treatment provided same/next day based on rapid test results
**Partner notification**	Started when positive test results returned (at 10-14 days)	Immediately commenced at same/next day follow-up appt

Figure 1. Overview of rapid pathway service redesign (MSM = Men who have sex with men; NAAT = Nucleic Acid Amplification Test; GC = Gonorrhoea Culture, appt = appointment)

For peer review only - http://bmjopen.bmj.com/site/about/guidelines.xhtml

Elements of rapid STI service implementation	
**What**	**Who should do this**
**Changes to documentation**	
Rewrite and sign-off treatment guidelines and SOPs when new processes are agreed.	Project leads, clinical lead, project implementation/operations team and clinical governance guideline group
Change the triage process and form.	Project operations team, to include reception staff
Consider changes to IT system/medical records system.	Project leads, consultant and project implementation team
**Implementation of the actual machine and process**	
Write business case for new rapid STI service and have it signed off by PHE.	Clinic lead, clinic manager, lead consultant, project manager, in collaboration with PHE.
Source the machine, find space for it (with waste disposal) and install it. Arrange insurance (including negotiations with PHE and legal teams)	Clinic lead, operations manager, lead consultant, PHE, nursing lead
Ensure IT systems allow direct transfer of data from Panther	Project lead, clinic manager
Pilot before implementing with all patients	All staff including reception teams
Write protocol for Panther outages	Project leads, in collaboration with PHE.
Quality assessment scheme /UKAS accreditation	PHE team
**Services**	
Consider impact on other services	Project operations team
Adjust clinic timetabling to accommodate rapid STI service appointments	Operations manager
Changing medical history forms and process to accommodate the new appointment structure	Project leads and implementation team
Changes to the IT coding	Clinic data manager, project lead, clinic manager, clinical lead (minor)
**Staff engagement, training and communication**	
Put together an implementation team, to oversee implementation, and put in place mechanisms for all staff to feedback to this team	Representative from each staff group and clinic manager.
Clarify communication pathways between all staff and the implementation team	Clinic Manager, project leads
Consider the impact on staff roles and workload and if staffing changes are therefore needed	Project operations team
Regular meetings for staff involved in the new service	Possible staff to include: project leads, HAs, consultants, nursing assistants, nurses, administrative staff, researchers, IT lead, clinic manager, data manager, chlamydia screening program team lead.
Staff training and regular updates at existing staff training sessions	Led by project leads, all staff to attend
Regular departmental meetings	Project leads and clinical lead
**Patient communication**	
Communicate changes to patients – write leaflets/posters/website	Project leads

For peer review only

COREQ (COnsolidated criteria for REporting Qualitative research) Checklist

A checklist of items that should be included in reports of qualitative research. You must report the page number in your manuscript where you consider each of the items listed in this checklist. If you have not included this information, either revise your manuscript accordingly before submitting or note N/A.

Topic	Item No.	Guide Questions/Description	Reported on Page No.
**Domain 1: Research team and reflexivity**			
Personal characteristics			
Interviewer/facilitator	1	Which author/s conducted the interview or focus group?	
Credentials	2	What were the researcher's credentials? E.g. PhD, MD	
Occupation	3	What was their occupation at the time of the study?	
Gender	4	Was the researcher male or female?	
Experience and training	5	What experience or training did the researcher have?	
Relationship with participants			
Relationship established	6	Was a relationship established prior to study commencement?	
Participant knowledge of the interviewer	7	What did the participants know about the researcher? e.g. personal goals, reasons for doing the research	
Interviewer characteristics	8	What characteristics were reported about the inter viewer/facilitator? e.g. Bias, assumptions, reasons and interests in the research topic	
**Domain 2: Study design**			
Theoretical framework			
Methodological orientation and Theory	9	What methodological orientation was stated to underpin the study? e.g. grounded theory, discourse analysis, ethnography, phenomenology, content analysis	
Participant selection			
Sampling	10	How were participants selected? e.g. purposive, convenience, consecutive, snowball	
Method of approach	11	How were participants approached? e.g. face-to-face, telephone, mail, email	
Sample size	12	How many participants were in the study?	
Non-participation	13	How many people refused to participate or dropped out? Reasons?	
Setting			
Setting of data collection	14	Where was the data collected? e.g. home, clinic, workplace	
Presence of non-participants	15	Was anyone else present besides the participants and researchers?	
Description of sample	16	What are the important characteristics of the sample? e.g. demographic data, date	
Data collection			
Interview guide	17	Were questions, prompts, guides provided by the authors? Was it pilot tested?	
Repeat interviews	18	Were repeat inter views carried out? If yes, how many?	
Audio/visual recording	19	Did the research use audio or visual recording to collect the data?	
Field notes	20	Were field notes made during and/or after the inter view or focus group?	
Duration	21	What was the duration of the inter views or focus group?	
Data saturation	22	Was data saturation discussed?	
Transcripts returned	23	Were transcripts returned to participants for comment and/or	

For peer review only

Topic	Item No.	Guide Questions/Description	Reported on Page No.
		correction?	
**Domain 3: analysis and findings**			
Data analysis			
Number of data coders	24	How many data coders coded the data?	
Description of the coding tree	25	Did authors provide a description of the coding tree?	
Derivation of themes	26	Were themes identified in advance or derived from the data?	
Software	27	What software, if applicable, was used to manage the data?	
Participant checking	28	Did participants provide feedback on the findings?	
Reporting			
Quotations presented	29	Were participant quotations presented to illustrate the themes/findings? Was each quotation identified? e.g. participant number	
Data and findings consistent	30	Was there consistency between the data presented and the findings?	
Clarity of major themes	31	Were major themes clearly presented in the findings?	
Clarity of minor themes	32	Is there a description of diverse cases or discussion of minor themes?	

Developed from: Tong A, Sainsbury P, Craig J. Consolidated criteria for reporting qualitative research (COREQ): a 32-item checklist for interviews and focus groups. *International Journal for Quality in Health Care*. 2007. Volume 19, Number 6: pp. 349 – 357

Once you have completed this checklist, please save a copy and upload it as part of your submission. DO NOT include this checklist as part of the main manuscript document. It must be uploaded as a separate file.

For peer review only

BMJ Open

What can be learnt from a qualitative evaluation of implementing a rapid sexual health testing, diagnosis and treatment service?

Journal:	*BMJ Open*
Manuscript ID	bmjopen-2021-050109.R3
Article Type:	Original research
Date Submitted by the Author:	30-Sep-2021
Complete List of Authors:	Lorenc, Ava; University of Bristol, Population Health Sciences, Bristol Medical School; National Institute for Health Research, Applied Research Collaboration West<br>Brangan, Emer; University of the West of England, Department of Nursing and Midwifery<br>Kesten, Joanna; University of Bristol, National Institute for Health Research, Applied Research Collaboration West (NIHR ARC West); University of Bristol, NIHR Health Protection Research Unit in Behavioural Science and Evaluation, Population Health Sciences, Bristol Medical School<br>Horner, Paddy; University of Bristol, NIHR Health Protection Research Unit in Behavioural Science and Evaluation, Population Health Sciences, Bristol Medical School; University Hospitals Bristol and Weston NHS Foundation Trust, Unity Sexual Health<br>Clarke, Michael; University Hospitals Bristol and Weston NHS Foundation Trust, Unity Sexual Health<br>Crofts, Megan; University Hospitals Bristol and Weston NHS Foundation Trust, Unity Sexual Health<br>Steer, Jonathan; Public Health England, South West Regional Laboratory, National Infection Service<br>Turner, Jonathan; Public Health England, South West Regional Laboratory, National Infection Service<br>Muir, Peter; Public Health England, South West Regional Laboratory, National Infection Service<br>Horwood, Jeremy; University of Bristol, National Institute for Health Research, Applied Research Collaboration West (NIHR ARC West), University Hospitals Bristol and Weston NHS Foundation Trust; University of Bristol, NIHR Health Protection Research Unit in Behavioural Science and Evaluation, Population Health Sciences
**Primary Subject Heading**:	Sexual health
Secondary Subject Heading:	Health services research, Qualitative research
Keywords:	QUALITATIVE RESEARCH, SEXUAL MEDICINE, Organisational development < HEALTH SERVICES ADMINISTRATION & MANAGEMENT, Change management < HEALTH SERVICES ADMINISTRATION & MANAGEMENT, COVID-19

SCHOLARONE™
Manuscripts

For peer review only - http://bmjopen.bmj.com/site/about/guidelines.xhtml

I, the Submitting Author has the right to grant and does grant on behalf of all authors of the Work (as defined in the below author licence), an exclusive licence and/or a non-exclusive licence for contributions from authors who are: i) UK Crown employees; ii) where BMJ has agreed a CC-BY licence shall apply, and/or iii) in accordance with the terms applicable for US Federal Government officers or employees acting as part of their official duties; on a worldwide, perpetual, irrevocable, royalty-free basis to BMJ Publishing Group Ltd ("BMJ") its licensees and where the relevant Journal is co-owned by BMJ to the co-owners of the Journal, to publish the Work in this journal and any other BMJ products and to exploit all rights, as set out in our licence.

The Submitting Author accepts and understands that any supply made under these terms is made by BMJ to the Submitting Author unless you are acting as an employee on behalf of your employer or a postgraduate student of an affiliated institution which is paying any applicable article publishing charge ("APC") for Open Access articles. Where the Submitting Author wishes to make the Work available on an Open Access basis (and intends to pay the relevant APC), the terms of reuse of such Open Access shall be governed by a Creative Commons licence – details of these licences and which Creative Commons licence will apply to this Work are set out in our licence referred to above.

Other than as permitted in any relevant BMJ Author's Self Archiving Policies, I confirm this Work has not been accepted for publication elsewhere, is not being considered for publication elsewhere and does not duplicate material already published. I confirm all authors consent to publication of this Work and authorise the granting of this licence.

For peer review only

What can be learnt from a qualitative evaluation of implementing a rapid sexual health testing, diagnosis and treatment service?

Authors

Ava Lorenc[1,2], Emer Brangan[1,3], Joanna M Kesten[1,2,4], Paddy Horner[1,4,5], Michael Clarke[5], Megan Crofts[5], Jonathan Steer[6], Jonathan Turner[6], Peter Muir[6], Jeremy Horwood[1,2,4]

[1.] Population Health Sciences, Bristol Medical School, University of Bristol, Canynge Hall, 39 Whatley Road, Bristol, BS8 2PS

[2.] National Institute for Health Research, Applied Research Collaboration West (NIHR ARC West), University Hospitals Bristol and Weston NHS Foundation Trust, 9th Floor, Whitefriars, Lewins Mead, Bristol, BS1 2NT

[3] University of the West of England
Department of Nursing and Midwifery, Faculty of Health and Applied Sciences, University of the West of England, Frenchay Campus, Coldharbour Lane, Bristol, BS16 1QY

[4] NIHR Health Protection Research Unit in Behavioural Science and Evaluation, Population Health Sciences, Bristol Medical School, University of Bristol, Canynge Hall, 39 Whatley Road, Bristol, BS8 2PS

[5] Unity Sexual Health, University Hospitals Bristol and Weston NHS Foundation Trust, Tower Hill, Avon, Bristol BS2 0JD

[6] Public Health England South West Regional Laboratory, National Infection Service, Pathology Sciences Building, Science Quarter, Southmead Hospital, Bristol BS10 5NB

Corresponding author: Jeremy Horwood, J.Horwood@bristol.ac.uk

Word count: 4521

For peer review only

For peer review only - http://bmjopen.bmj.com/site/about/guidelines.xhtml

Abstract

Objectives

To investigate experiences of implementing a new rapid sexual health testing, diagnosis and treatment service.

Design

A theory-based qualitative evaluation with a focused ethnographic approach using non-participant observations and interviews with patient and clinic staff. Normalisation Process Theory was used to structure interview questions and thematic analysis.

Setting

A sexual health centre in Bristol, UK.

Participants

26 patients and 21 staff involved in the rapid sexually transmitted infection (STI) service were interviewed. Purposive sampling aimed for a range of views and experiences and socio-demographics and STI results for patients, job grades and roles for staff. 40 hours of observations conducted.

Results

Implementation of the new service required co-ordinated changes in practice across multiple staff teams. Patients also needed to make changes to how they accessed the service. Multiple small 'pilots' of process changes were necessary to find workable options. For example, the service was introduced in phases beginning with male patients. This responsive operating mode created challenges for delivering comprehensive training and communication in advance to all staff. However, staff worked together to adjust and improve the new service, and morale was buoyed through observing positive impacts on patient care. Patients valued faster results and avoiding unnecessary treatment. Patients reported that they were willing to drop-off self-samples and return for a follow-up appointment, enabling infection-specific treatment in accordance with test results thus improving antimicrobial stewardship.

Conclusions

The new service was acceptable to staff and patients. Implementation of service changes to improve access and delivery of care in the context of stretched resources can pose challenges for staff at all levels. Early evaluation of pilots of process changes, played an important role in the success of the service by rapidly feeding back issues for adjustment. Visibility to staff of positive impacts on patient care is important in maintaining morale.

Strengths and limitations of this study

- The 'trial, assess, adapt' strategy (reflexive process of observation, feedback, and resulting action) meant that evaluation and implementation occurred in parallel and allowed researchers to capture the active process.
- The evaluation benefitted the staff, as researchers provided ongoing feedback and suggestions for service improvements and provided a space for reflection.

For peer review only

- A strong and trusting relationship between research and clinic staff arose from researcher flexibility and timely responsiveness and allowed good researcher access to spaces, staff and meetings.
- Frequent, regular and extensive physical presence of the researcher in various clinic settings was crucial as much of the process was not documented.
- The patient sample was limited due to recruitment being cut short by the COVID-19 pandemic lockdown, and we only interviewed males due to the pathway being initially implemented for male patients during the evaluation period.

For peer review only

For peer review only - http://bmjopen.bmj.com/site/about/guidelines.xhtml

Introduction

Rates of sexually transmitted infections (STIs) continue to increase in England despite control efforts, with a 5% increase between 2018-2019[1]. *Chlamydia trachomatis* (chlamydia) and *Neisseria gonorrhoeae* (gonorrhoea) are the most common, with 226,411, and 70,982 diagnoses reported in England in 2019, a 5% and 26% increase since 2018[2]. The rise in gonorrhoea is particularly concerning as first line treatment effectiveness is threatened by the development of antimicrobial resistance (AMR)[3,4]. Most STIs are diagnosed through Specialist Sexual Health Services (SSHS), the provision of which is increasingly challenging as funding (via government public health grant), has been steadily cut since 2015[5].

Chlamydia and gonorrhoea if left untreated may cause pelvic inflammatory disease (PID) in women, which can result in infertility, ectopic pregnancy, and chronic pelvic pain[6-8]. Infections are often asymptomatic, particularly in women, and when they do cause symptoms and/or signs these are not pathognomonic[6 7]. Nucleic acid amplification tests (NAATs) provide accurate detection. Early detection and treatment helps prevent the spread of STIs and the development of complications. Point-of-care testing (POCT; results within 15-30mins)[9] and rapid STI services (results on the same day) can potentially improve care and reduce costs, due to reduced time from diagnosis to treatment and number lost to follow up. This can increase testing uptake, improve partner notification rates and enable better and timelier clinician decisions, improving outcomes such as fewer unnecessary treatments and reduced PID risk[10-13]. Patients prefer rapid STI testing[14-16] and are happy to wait at clinic for results. Rapid testing can reduce anxiety[17 18] and improve patient acceptability of services and uptake of testing[19-22]. HIV POCT is well established and preferred by high risk men who have sex with men (MSM)[23 24]. Although studies suggest a limit of 30 minutes to wait for results[25-28], experience from our service indicates patients would be prepared to wait longer than 20 minutes for their result[29].

However, much of the evidence is from modelling and hypothetical views of clinicians and/or patients[10-12 25-28 30], with little real-life implementation evaluation[31], and rarely considering the complexity of patient visits including both asymptomatic and symptomatic patients with multiple needs e.g. female contraception. There is an urgent need to evaluate staff and patient preferences, and clinical benefits and cost effectiveness in practice.

In November 2018, a UK SSHS implemented a first-of-its-kind rapid STI testing, diagnosis and treatment service, using a clinic-based Hologic 'Panther' NAAT diagnostic machine. In 2017, the clinic introduced an online STI and HIV testing postal service for asymptomatic patients[32]. The new rapid service provides chlamydia and gonorrhoea results in 3.5 hours (previously over a week when tested in the microbiology laboratory), to improve patient care while reducing costs (see figure 1 for an overview of the service redesign). This evaluation assessed the best service model and patient and staff acceptability, to refine and improve the service and support implementation in other SSHSs. We report the qualitative evaluation of male patient and staff views and experiences of the implementation of the first phase of this new rapid STI service.

For peer review only - http://bmjopen.bmj.com/site/about/guidelines.xhtml

Methods

Design

The evaluation was ethnographic, used observations and interviews[33] and was informed by Normalisation Process Theory (NPT). NPT is a sociological theory that has been widely promoted as a means to understand implementation, embedding and integration of innovation in healthcare settings until they become normalised and routine[34]. This approach focuses on actions people perform to normalise an intervention within the contexts and locations they inhabit[34]. NPT proposes that successful implementation of an intervention is dependent on participants ability to fulfil four inter-related criteria which interact with the wider intervention context[34]: 1) Coherence - (sense-making - understanding and opinion of the intervention's purpose); 2) Cognitive Participation (commitment and engagement with the intervention); 3) Collective Action (the work that individuals and organisations have to do to make the intervention function); 4) Reflexive Monitoring (appraisal of the intervention once it is in use). NPT supported real-time feedback to refine and improve the service. The study focussed on four timepoints selected pragmatically during 16 months of evaluation: T1 at start of implementation; T2 after 6 months; T3 after 14 months; T4 at 16 months during the COVID-19 pandemic lockdown.

Setting

A sexual health clinic in Bristol (population 450,000), UK.

Participants

Due to the new service being initially introduced for the male pathway only, male patients (over 16 years old) and staff at the sexual health clinic were interviewed. Patients were invited to take part, via a clinic survey about PrEP (pre-exposure prophylaxis for HIV) [35] and when physically attending the clinic at T1, T2 and T3. Cross sectional interviews were conducted with staff at four timepoints at T1, T2, T3, and T4. One staff member was interviewed twice. Purposive sampling[33] attempted to capture maximum variation in views and experiences, and socio-demographics and STI test results for patients, and job grades and roles for staff (administrative staff, consultants, doctors, nurses/nursing assistants, health advisers, Public Health England (PHE)); responsible for the Panther laboratory and administration. Information sheets were provided to male patients by staff at the clinic or via email from researchers, with patients asked to contact the researcher and ask questions before deciding to take part. Staff were emailed by the researcher about the study.

Data collection

Following the concept of information power, data collection continued until sufficient data to meet the study objectives had been collected with continuous, pragmatic assessment of information within our sample [36]. Issues informing information power include the study aim (i.e. broader aims require a larger sample), the sample (i.e. a smaller sample is needed if participants have rich experiences relevant to the research), use of theory (studies supported by theory require smaller sample sizes), depth and quality of the data (i.e. smaller samples are needed with focused and clear data) and the analysis type (larger samples are needed for exploratory analysis) [36].

For peer review only - http://bmjopen.bmj.com/site/about/guidelines.xhtml

In the first 6 months of service implementation, observations were conducted by EB and JK at varying times/days, in reception, laboratory and waiting areas. Non-participant observations focussed on day-to-day operations, how clinic staff integrated the new service and any factors which promoted or inhibited successful incorporation [37]. Written accounts based on brief field notes taken at the time included observations, conversations with staff, and reflection on what had been observed[38]. Observations recorded activities, events, their time and location and described interactions, communication patterns, workflows and tasks in the clinic environment.

Interview topic guides (Supplementary Files 1 and 2) informed by NPT explored: views and experiences of the service; impact on workload and clinical practice; information and support needs, sustainability and future implementation of the service. Patient interviews took place throughout the evaluation period and explored their experience and views of the service including acceptability, barriers and facilitators to uptake. Patients were offered a £10 High Street shopping voucher. Interviews were conducted by experienced qualitative senior research associates AL/JMK/EB, used flexible topic guides and open-ended questioning, were face-to-face (at the clinic or University) or by telephone, and lasted around 30 minutes. Participants were told that the study was evaluating the rapid results service and that interviewers were independent of the service.

Analysis

Interviews were audio recorded, transcribed verbatim and imported into QSR NVivo (version 10) with transcribed observation fieldnotes. Ongoing and iterative analysis informed further data collection through changes to the topic guide and feedback to healthcare staff to aid the adaptation and refinement of the rapid service. 'Codebook' thematic, inductive analysis by EB/AL identified and analysed patterns and themes salient to interviews and observations [39]. Initial noting of ideas was followed by line-by-line examination and inductive coding. A subset of transcripts and observations were independently double-coded by EB/JH and discrepancies discussed to contribute to the generation and refinement of codes to maximise rigour. Themes were discussed by the multi-disciplinary research team to ensure credibility and confirmability. Negative cases and reasons for deviance were explored. The four NPT constructs[34] were used to further develop themes deductively.

Ethical approval

South West Frenchay Research Ethics Committee granted approval, reference 18/SW/0090.

Patient and Public Involvement

PPI meetings with three people who recently used the clinic informed the study design. These meetings reviewed patient-facing materials and discussed the acceptability of proposed recruitment and data collection.

Results

Participants/hours of observation

We conducted 25 observations over approximately 40 hours, 25 staff interviews (24 participants) and 26 patient interviews. Patients were aged 34 years on average (range 19 to 57 years), most

For peer review only - http://bmjopen.bmj.com/site/about/guidelines.xhtml

identified as MSM, two had positive STI test results and the index of multiple deprivation scores averaged 5.4 (range 2 to 10).

Coherence (sense-making)

Staff and patients welcomed rapid testing (Table 1). All staff saw it as beneficial and many were excited about doing something new, particularly to improve service access which was limited by a lack of pre-bookable appointments, high observed demand (manifesting in long queues outside the clinic before it opened each morning to access limited capacity walk-in appointments) and staff shortages. Staff welcomed being able to provide treatment based on results and avoiding unnecessary antibiotic prescribing (previously treatment was prescribed presumptively for symptomatic patients due to the week-long wait for test results). However, some staff had concerns around anticipated reduced clinician contact with patients and shorter consultations in the new service. Patients valued a potentially quicker and more convenient service, but also reduced anxiety from long waiting times for results,. Some patients valued avoiding unnecessary antibiotic treatment for personal and wider societal reasons.

Cognitive participation (buy-in)

The importance of engaging the whole clinic team in the service redesign was recognised but challenging with a large team and many part-time staff (Table 1). Formal engagement was via an implementation team, project meetings and staff training sessions. Engagement of 'on the ground' staff was inadequate, with administrative and nursing staff feeling particularly disengaged and having limited time to prepare for the new service. Staff cited the following issues around engagement:poor communication (due to busy work schedules with limited time for accessing emails), a lack of access to training as many staff were part-time and did not work on the day training was delivered or lack of involvement in project meetings or the implementation group which was initially only senior staff, although the latter improved as the project progressed. Engagement was also limited by a lack of staff protected project time and a context of burn out from staff pressures (e.g. funding cuts, understaffing, and high service demand). Implementation work was fitted around existing high workloads and rapid service changes made timely feedback to staff difficult.

Collective action (putting rapid STI test results service into operation)

The service was implemented for male patients in November 2018 and for all patients in August 2019.

Important in collective action was designing and documenting the new patient pathways, which needed to be clear but flexible to allow staff deviation from protocol to respond to individual patient situations and need (e.g. anxiety, medical history, relationship status, availability to attend clinic). Guidelines, Standard Operating Procedures (SOPs) and pathways had to be rewritten as initial implementation issues were resolved. However, detailed SOPs were not always in place prior to implementation of a new modification to a pathway, making it difficult for staff to keep up with current processes. This was due to the repeated and frequent changes to clinic processes/patient pathways to resolve initial implementation issues, and the lack of protected administrative staff time meant that when patient pathways were revised following staff feedback these could take over 6 weeks to review and be signed off by the clinical governance group. For example, the triage form asking patients to self-identify at reception whether they were symptomatic, their risk level, and if they had had sex against their will was revised three times during implementation to make it clearer

For peer review only - http://bmjopen.bmj.com/site/about/guidelines.xhtml

which pathway should be followed. Observation showed that they annotated a copy of the triage form to remind them of the pathways for different responses. In contrast, patients were happy with communication about the changes made (via the website, staff, consultations, on the triage form).

Although staff accepted the continual adaptations as inevitable due to the novelty of the service, it was difficult. During initial implementation, 'teething issues' were experienced, including administrative staff not knowing which patients were eligible for the service, dealing with the high volume of patients when the doors first open, and the best way to triage patients. Staff worked together to adjust and improve the new service, identifying problems and opportunities and innovating in their own practice, overseen and supported by the implementation groups, and morale was buoyed by the positive impact on patient care and the positive feedback from the research team.

The evaluation process played an important role in the success of the service by rapidly feeding back issues for resolution. The evaluation process aided communication, and researchers were able to suggest solutions to problems based on the non-participant observation. For example, researcher (EB) co-developed with the clinical team a laminated card for patients explaining the new service in response to the researcher observation that patients were given variable information by reception staff. Some of the changes to the patient pathway (Table 2 and 3) caused challenges. The responsive model meant comprehensive preparatory training and communication to all staff was challenging, and multiple methods of communication were essential. Many staff found changing ingrained behaviours difficult, particularly reducing the content and duration of consultations when they had been taught to maximise patient contact. The shorter initial appointments, with reduced medical record completion and fewer physical examinations, was a 'huge change' and source of concern and anxiety for clinicians both before and during the changes, due to perceived loss of opportunities for patient discussions about domestic violence, female genital mutilation, alcohol use, and contraception etc. which are seen as essential for a 'holistic', 'integrated' 'level 3 service'. This did improve with practice, and patients with particularly concerning issues were referred for a health adviser consultation, which was longer under the new service. Self-sampling drop-off also meant reduced clinical contact, particularly for asymptomatic, low-risk men with negative test results (health advisers only see high-risk/new MSM patients at the first visit). The walk-in clinic was therefore more demanding, as the case mix changed, seeing more symptomatic patients with complex presentations. Although reduced clinical contact with asymptomatic patients was a planned cost-saving benefit, it meant nursing assistants (running the sample drop-off sessions) collected mandatory data (GUMCAD surveillance system[40]) and answered patient clinical queries, which they were not qualified/paid/willing to do.

In the new service, chlamydia and gonorrhoea treatments were to be given based on results, not presumptively unless sexual contact with a case was within the 2-week window period and patients requested treatment[6 7]. Men with symptoms of urethritis were first tested for chlamydia/gonorrhoea and booked to return more than 4 hours later. If NAAT-positive they were treated according to British Association for Sexual Health and HIV (BASHH) chlamydia and gonorrhoea guidelines[6 7] and if negative tested for urethritis and managed according to BASHH guidelines[41] [with reassurance, including a leaflet, if negative] and told to re-attend for an early morning smear if their symptoms did not resolve. Some patients, particularly regular clinic attendees, were initially not keen on this longer wait for treatment, although this did improve. A minority of clinicians deviated from protocol and treated presumptively, especially for patients who were particularly anxious. Staff reported mixed patient understanding of only treating when results

For peer review only - http://bmjopen.bmj.com/site/about/guidelines.xhtml

were available, with detailed explanations needed, but patients were amenable once they understood.

Reflexive monitoring (appraisal of STI test results service into operation)

Contextual factors

In addition to the contextual factors described above of inadequate service funding, understaffing and, ongoing communication problems, increased use of postal testing (meaning less complex patients used postal testing and more complex patients used the walk-in clinic), and increasing use of PrEP increasing service demand. Increasing societal awareness of gender issues also influenced the service experience, with triage forms issued to male patients on arrival creating tensions around sensitively managing patients who did not identify with their sex assigned at birth (including trans and non-binary patients). This process was amended following feedback from the research team.

Success

Overall, the new service was seen as successful as it was implemented and running fairly smoothly after initial problems (Table 4). Although the process was challenging, implementation was an achievement, given the constraints on resources and staffing and lack of additional funding highlighted above. Staff were credited with being adaptable, highly motivated, hardworking, and mutually supportive. Staff job satisfaction and morale was boosted from doing something new and exciting and they felt proud about achieving implementation which contributed to enhanced teamwork and coherence. Better job satisfaction was mainly due to improvements to consultations with patients, including consultants seeing more complex patients. These boosts gave the team confidence that they could make further service improvements, demonstrated by the rapid changes made during the first wave of the COVID-19 pandemic during which staff reported being more 'change-ready'.

Although staff were initially concerned that the changes would jeopardise the quality of care, this does not appear to have been realised and patients felt very positive about staff and the ability to raise concerns and discuss issues. Staff perceived that the service was able to see more patients, and that clinicians and health advisers could spend more time and better engage with complex and higher risk patients due to more efficient processing of patients attending for routine testing . Self-testing and fewer physical examinations involving invasive sampling (urethral swab) was generally preferred by patients. Decreased time to diagnosis and treatment meant less patient anxiety while waiting for results and most patients were happy to wait up to 48 hours for treatment. Indeed, patients rated the quality of the new service highly, with some patients specifically requesting it. Staff, and some patients, were pleased to be able to treat with results, which promoted informed discussions and reduced antibiotic use, secondary complications, and onward transmission.

Suggested improvements

For many staff the most important improvements to the implementation were preparation of documentation of new processes and pathways as soon as possible and engaging and supportive communication from senior staff with all staff but particularly nursing and reception teams to improve process design iterations. This communication should use a variety of methods (especially face-to-face) including written, training sessions, on-the-job support, informal, and nominated individuals for support. Bringing teams together for training was recommended to facilitate information exchange and understanding. It was recommended that, if possible, staff needed to be better prepared for behaviour change and multiple continual adaptations. Staff also need protected

For peer review only - http://bmjopen.bmj.com/site/about/guidelines.xhtml

time for the project, and the impact on staff roles and workloads needs to be better considered. Small-scale pilots of the new service with patients, to test and refine draft processes to reduce staff stress and confusion were proposed. Other areas for improvement were: consistency in the rapidity of results and contingency planning for malfunctions (sometimes results were not available on time due to Panther machine breakdowns); more and earlier information for patients, especially on the process and timings (waiting times, results notification etc). Finally, the use of phone/video clinics, which were implemented during physical distancing requirements of COVID-19, may have benefits elsewhere.

The Supplementary File 3 summarises service considerations for implementing a rapid STI service, and relevant teams/job roles.

Discussion

Principal findings

The first UK rapid NAAT testing integrated SSHS for chlamydia and gonorrhoea was successfully implemented despite funding and staff shortages. Inevitable initial challenges were resolved and, overall, it was well received. Staff were enthusiastic about it and understood the benefits, although some were concerned about reduced patient contact. The use of NPT allowed for examination of issues with both the design of the rapid service and its implementation. Cognitive participation difficulties included engaging all staff and changing ingrained behaviours (resulting from extensive training and audit), especially for administrative and nursing staff, although staff did support each other and work together. Some patients had concerns about waiting for treatment, but most accepted sample drop-off and returning for a follow-up appointment. Reflexive monitoring revealed perceived benefits including reduced patient anxiety, seeing more patients, and boosting staff job satisfaction. Infection-specific treatment based on test results was crucial, enabling informed consultations and improving antimicrobial stewardship. Suggestions for this and other future services included: documenting new pathways and processes early and comprehensively disseminating to staff; involving all staff in planning, design and implementation; protecting staff time for meetings and actions; considering pilots with a small group of staff/patients before sharing more widely or writing guidelines; cross-discipline training; varied methods of, sensitive and supportive communication; considering staff role impact, and ensuring staffing to cover changes.

Relation to other studies

Evaluating the real-life implementation of a novel rapid results service confirms previous hypothetical/simulated studies where patients were happy with the service and willing to wait for results before treatment[14-16 25]. Whereas previous research has found that the patients found the hypothetical scenario of waiting up to 40 minutes for test results acceptable[25 26], our findings demonstrate that patients were happy to wait up to 48 hours for treatment based on results. Willingness to wait has been found to be dependent on self-assessed infection risk and anxiety about their infection status [25]. Our findings demonstrate that the rapid service can lead to less patient anxiety due to shorter time waiting for results and therefore should target patients concerned they are infected. Although asymptomatic patients are encouraged to use on-line postal services, some patients may wish to attend in-person clinics[13 42]. The previously anticipated[12 18]benefits of treating with results and improving antimicrobial stewardship are highly valued by staff and patients in our evaluation. Modelling studies have demonstrated that rapid testing can enable faster treatment, reduces infectious periods, and leads to fewer transmissions, partner attendances and clinic costs[43 44]. Rapid diagnostics and treatment can increase the proportion of individuals

For peer review only - http://bmjopen.bmj.com/site/about/guidelines.xhtml

receiving timely treatment and decrease community prevalence of STIs[45 46] and recently has been seen as a key factor contributing to the reducing new HIV infections in London and ensuring those with HV receive fast and optimal care[47]. Our findings also confirm reductions in patient anxiety[12 17 18] and improved testing uptake[19-21] are likely, as well as freeing up clinician time, greater clinician confidence, and efficiencies allowing capacity to be utilised elsewhere[12].

The challenges of communicating with and engaging all staff, especially those 'on the ground', and the need for dedicated time for training and implementation[48] are key in healthcare quality improvement[48]. Teething issues experienced in this service – documentation of new pathways, impact on staff roles – and the challenges of changing ingrained behaviour – are common in implementation of a major service change and emphasise the importance of staff training and communication of the reason and implications for change[49].

Our findings demonstrate that successfully implementing a beneficial service change can boost staff job satisfaction and morale. Previous research has found improvements in staff satisfaction following successful sexual healthcare innovation[49]. This finding suggests the implementation realised benefits for staff - previously highlighted as influencing acceptance of change in NHS service improvement programmes[50] – and aligned with professionals values and intrinsic motivation to provide quality and effective care[48].

Implications

This study shows that a rapid NAAT-testing integrated SSHS for chlamydia and gonorrhoea can be implemented in a constrained NHS system, and is acceptable to patients, with benefits for staff, patients and public health, including reduced patient anxiety. The perceived efficiency (to be clarified in a separate quantitative evaluation) is crucial given the financial and staffing pressures on UK sexual health services[51]. Similarly, the pride of staff in their service, and enhanced staff satisfaction are important in boosting staff morale and is likely to further enhance the provision of high-quality patient care when such a service is introduced.

AMR is a major concern for gonorrhoea, and a priority worldwide[52] and in England[53]. Rapid STI services could play a vital role in reducing unnecessary antibiotic prescribing by providing test results during/soon after consultations, allowing informed clinician choices. When the technology becomes available, the addition of POCTs to detect ciprofloxacin-sensitive gonorrhoea will dramatically reduce reliance on ceftriaxone and selection pressure for AMR[54 55].

Our implementation recommendations for future services echo those from the Health Foundation, such as sensitive leadership oriented towards inclusion, agreeing roles and responsibilities at the outset and 'bringing everyone along with you'[48], as well as early documentation, piloting pathways, varying communication methods and adequate staffing. The willingness of symptomatic male patients to wait for treatment can inform development of new care pathways using POCTs[12 56], although results are limited to a single service and male patients.

Strengths and limitations

Project strengths include: integration of findings from multiple qualitative methods generating rich insights, a multidisciplinary team including clinical academics; a strong trusting relationship between research team and clinical staff due to existing relationships and research team flexibility and responsiveness; regular feedback from researchers to clinicians using a 'trial, assess, adapt' strategy. EB and JK came to the observations as experienced researchers and with good knowledge of the plans for the service changes and reasons for them. The researchers were surprised at how quickly it was possible to provide information and feedback to the implementation team which they clearly

For peer review only - http://bmjopen.bmj.com/site/about/guidelines.xhtml

valued highly and rapidly implemented changes based on it. The researchers could move freely between different physical areas of the clinic and stages of the process in a way which clinic staff were not free to do, which provided early insights. Due to the study design and relationships, these insights could be discussed promptly with relevant staff - and so sense checked, and action taken in response if appropriate (changes to clinic processes; further data collection etc.). The rapid, supportive, evidence-based feedback which the researchers could provide seemed to quickly build the confidence of the key implementation staff in the research process. The researchers appeared to be quickly accepted as trusted team members, with the capacity to help with the work at hand (rather than creating 'research burden').

Limitations include an all-male patient sample as the service was initially only for males, and when implemented for females, few were eligible and evaluation was hampered by the COVID-19 pandemic. We aimed to include patients with positive STI results but most (although symptomatic) were negative, limiting evaluation of follow-up appointments. COVID-19 meant fewer final batch interviews. As the rapid STI result technology develops, continued implementation evaluation is important[56], capturing, the wide-ranging impact on services, staff and patients. Evaluation for female patients is needed, given the challenges around contraception and STIs/symptoms.

Conclusion

As the first UK SSHS to implement rapid NAAT testing for chlamydia and gonorrhoea within an integrated service, this project faced the challenge of innovating to save time/money and improve patient experience in a constrained environment, particularly lack of funding and understaffing. Inevitable challenges – mainly related to the impact on patient pathways - were resolved and, overall, it was a success. Perceived benefits included reduced patient anxiety, seeing more patients, treating with results, reduced antibiotics use and boosting staff job satisfaction. Learning for other services considering implementing something similar includes more inclusive staff engagement, sensitive communication, better documentation of changes, dealing with constant adaptations, and consideration of the impact on staff and their roles.

Figure 1. Overview of rapid pathway service redesign (MSM = Men who have sex with men; NAAT = Nucleic Acid Amplification Test; GC = Gonorrhoea Culture, appt = appointment)

For peer review only - http://bmjopen.bmj.com/site/about/guidelines.xhtml

Acknowledgements

We would like to thank all study participants.

Funding statement

Funded by the National Institute for Health Research (NIHR) Applied Research Collaboration (ARC) West at University Hospitals Bristol and Weston NHS Foundation Trust and NIHR Health Protection Research Unit (HPRU) in Behavioural Science and Evaluation at University of Bristol in partnership with Public Health England (PHE). Award number P315. The views expressed are those of the authors and not necessarily those of the NIHR, the Department of Health and Social Care or PHE.

Competing interests statement

AL – no competing interests
EB - no competing interests
JK - no competing interests
PH - no competing interests
MC - no competing interests
MC - no competing interests
JS - no competing interests
JT - no competing interests
PM - no competing interests
JH - no competing interests

Author contributions

JH, PH, EB, JK were responsible for the study design. JH and EB were responsible for study management and coordination. EB, AL, JK and JH led data collection and analysis. MC and MC co-led the development and implementation of the new service model. PM, JS and JT supported implementation and accreditation of Point of Care testing. MC, MC, JS, JT, PM supported the study design and interpretation of interview findings. All authors read, commented on and approved the final manuscript.

Data Sharing Statement

Data are available on request from corresponding author.

For peer review only

References

1. England PH. Sexually transmitted infections and screening for chlamydia in England, 2019. 2019
2. Public Health England. Sexually transmitted infections (STIs): annual data tables. London: Public Health England, 2018.
3. Mohammed H, Sile B, Furegato M, et al. Poor adherence to gonorrhoea treatment guidelines in general practice in England. *The British journal of general practice : the journal of the Royal College of General Practitioners* 2016;66(648):352. doi: 10.3399/bjgp16x686077
4. Eyre DW, Town K, Street T, et al. Detection in the United Kingdom of the Neisseria gonorrhoeae FC428 clone, with ceftriaxone resistance and intermediate resistance to azithromycin, October to December 2018. *Euro Surveill* 2019;24(10):1900147. doi: 10.2807/1560-7917.ES.2019.24.10.1900147
5. White C. Sexual health services on the brink. *BMJ* 2017;359 doi: 10.1136/bmj.j5395
6. Fifer H, Saunders J, Soni S, et al. 2018 UK national guideline for the management of infection with Neisseria gonorrhoeae. Updated 2020. *International journal of STD & AIDS* 2020;31(1):4-15. doi: 10.1177/0956462419886775 [published Online First: 2019/12/25]
7. Nwokolo NC, Dragovic B, Patel S, et al. 2015 UK national guideline for the management of infection with Chlamydia trachomatis. Updated 2018. *International journal of STD & AIDS* 2018;27(4):251-67. doi: 10.1177/0956462415615443 [published Online First: 2015/11/06]
8. Price MJ, Ades AE, Soldan K, et al. The natural history of Chlamydia trachomatis infection in women: a multi-parameter evidence synthesis. *Health technology assessment (Winchester, England)* 2016;20(22):1-250. doi: 10.3310/hta20220 [published Online First: 2016/03/24]
9. Harding-Esch EM, Fuller SS, Chow SC, et al. Diagnostic accuracy of a prototype rapid chlamydia and gonorrhoea recombinase polymerase amplification assay: a multicentre cross-sectional preclinical evaluation. *Clinical microbiology and infection : the official publication of the European Society of Clinical Microbiology and Infectious Diseases* 2019;25(3):380.e1-80.e7. doi: 10.1016/j.cmi.2018.06.003 [published Online First: 2018/06/16]
10. Van Der Pol B. Making the Most of Point-of-care Testing for Sexually Transmitted Diseases. *Clinical Infectious Diseases* 2019 doi: 10.1093/cid/ciz523
11. Rönn MM, Menzies NA, Gift TL, et al. Potential for Point-of-Care Tests to Reduce Chlamydia-associated Burden in the United States: A Mathematical Modeling Analysis. *Clinical Infectious Diseases* 2019 doi: 10.1093/cid/ciz519
12. Adams EJ, Ehrlich A, Turner KME, et al. Mapping patient pathways and estimating resource use for point of care versus standard testing and treatment of chlamydia and gonorrhoea in genitourinary medicine clinics in the UK. *BMJ Open* 2014;4(7) doi: 10.1136/bmjopen-2014-005322
13. Whitlock GG, Gibbons DC, Longford N, et al. Rapid testing and treatment for sexually transmitted infections improve patient care and yield public health benefits. *International journal of STD & AIDS* 2018;29(5):14. doi: 10.1177/0956462417736431 [published Online First: 2017/10/24]
14. Llewellyn CD, Sakal C, Lagarde M, et al. Testing for sexually transmitted infections among students: a discrete choice experiment of service preferences. *BMJ Open* 2013;3(10) doi: 10.1136/bmjopen-2013-003240
15. Chislett L, Clarke J. Which elements of a novel self-directed rapid asymptomatic sexually transmitted infection screening service are most important to users? . Sexually Transmitted Infections, 2015:A1–A258.
16. Mahilum-Tapay L, Laitila V, Wawrzyniak JJ, et al. New point of care Chlamydia Rapid Test--bridging the gap between diagnosis and treatment: performance evaluation study. *BMJ (Clinical research ed)* 2007;335(7631):1190-94. doi: 10.1136/bmj.39402.463854.AE [published Online First: 11/30]

For peer review only - http://bmjopen.bmj.com/site/about/guidelines.xhtml

17. Llewellyn C, Pollard A, Miners A, et al. Understanding patient choices for attending sexually transmitted infection testing services: a qualitative study. *Sex Transm Infect* 2012;88(7):504-9. doi: 10.1136/sextrans-2011-050344 [published Online First: 2012/05/26]
18. Turner KME, Round J, Horner P, et al. An early evaluation of clinical and economic costs and benefits of implementing point of care NAAT tests for Chlamydia trachomatis and Neisseria gonorrhoea in genitourinary medicine clinics in England. *Sexually Transmitted Infections* 2013 doi: 10.1136/sextrans-2013-051147
19. Natoli L, Guy RJ, Shephard M, et al. Public health implications of molecular point-of-care testing for chlamydia and gonorrhoea in remote primary care services in Australia: a qualitative study. *BMJ Open* 2015;5(4) doi: 10.1136/bmjopen-2014-006922
20. Rompalo AM, Hsieh Y-H, Hogan T, et al. Point-of-care tests for sexually transmissible infections: what do 'end users' want? *Sexual health* 2013;10(6):541-45. doi: 10.1071/SH13047
21. Horwood J, Ingle SM, Burton D, et al. Sexual health risks, service use, and views of rapid point-of-care testing among men who have sex with men attending saunas: a cross-sectional survey. *International journal of STD & AIDS* 2016;27(4):273-80. doi: 10.1177/0956462415580504 [published Online First: 2015/04/25]
22. Lorenc T, Marrero-Guillamón I, Aggleton P, et al. Promoting the uptake of HIV testing among men who have sex with men: systematic review of effectiveness and cost-effectiveness. *Sexually Transmitted Infections* 2011;87(4):272-78. doi: 10.1136/sti.2010.048280
23. Read TRH, Hocking JS, Bradshaw CS, et al. Provision of rapid HIV tests within a health service and frequency of HIV testing among men who have sex with men: randomised controlled trial. *BMJ : British Medical Journal* 2013;347 doi: 10.1136/bmj.f5086
24. Public Health England. Strategic framework to promote the health and wellbeing of gay, bisexual and other men who have sex with men 2014a [Available from: https://www.gov.uk/government/uploads/system/uploads/attachment_data/file/313692/Strategic_Framework_to_promote_the_health_and_wellbeing_of_MSM_FINAL_DRAFT_For_comment.pdf.
25. Fuller SS, Pacho A, Broad CE, et al. "It's not a time spent issue, it's a 'what have you spent your time doing?' issue. . ." A qualitative study of UK patient opinions and expectations for implementation of Point of Care Tests for sexually transmitted infections and antimicrobial resistance. *PloS one* 2019;14(4):16. doi: 10.1371/journal.pone.0215380
26. Widdice L, Hsieh YH, Silver B, et al. Performance of the Atlas Genetics Rapid Test for Chlamydia trachomatis and women's attitudes toward point-of-care testing. *Sexually Transmitted Diseases* 2018;45(11):5. doi: 10.1097/OLQ.0000000000000865
27. Hsieh YH, Hogan MT, Barnes M, et al. Perceptions of an ideal point-of-care test for sexually transmitted infections--a qualitative study of focus group discussions with medical providers. *PloS one* 2010;5(11):e14144. doi: 10.1371/journal.pone.0014144 [published Online First: 2010/12/15]
28. Hsieh Y-H, Gaydos CA, Hogan MT, et al. What Qualities Are Most Important to Making a Point of Care Test Desirable for Clinicians and Others Offering Sexually Transmitted Infection Testing? *PloS one* 2011;6(4):e19263. doi: 10.1371/journal.pone.0019263
29. Hall C, Carter N, Crofts M, et al. P. P155 "time in clinic" survey to evaluate the potential for use of online registration. . *Sexually Transmitted Infections* 2015;91:A66-A67.
30. Huntington SE, Burns RM, Harding-Esch E, et al. Modelling-based evaluation of the costs, benefits and cost-effectiveness of multipathogen point-of-care tests for sexually transmitted infections in symptomatic genitourinary medicine clinic attendees. *BMJ Open* 2018;8
31. Natoli L, Guy RJ, Shephard M, et al. "I Do Feel Like a Scientist at Times": A Qualitative Study of the Acceptability of Molecular Point-Of-Care Testing for Chlamydia and Gonorrhoea to Primary Care Professionals in a Remote High STI Burden Setting. *PloS one* 2016;10(12):e0145993. doi: 10.1371/journal.pone.0145993

For peer review only - http://bmjopen.bmj.com/site/about/guidelines.xhtml

32. Turner KME, Looker KJ, Syred J, et al. Online testing for sexually transmitted infections: A whole systems approach to predicting value. *PloS one* 2019;14(2):e0212420. doi: 10.1371/journal.pone.0212420 [published Online First: 2019/02/23]
33. Higginbottom GM, Pillay JJ, Boadu NY. Guidance on Performing Focused Ethnographies with an Emphasis on Healthcare Research *The Qualitative Report* 2013;18(9):1-6.
34. Murray E, Treweek S, Pope C, et al. Normalisation process theory: a framework for developing, evaluating and implementing complex interventions. *BMC medicine* 2010;8:63. doi: 10.1186/1741-7015-8-63 [published Online First: 2010/10/22]
35. MacGregor L, Speare N, Nicholls J, et al. The role of the evolving trends in sexual behaviours and clinic attendance patterns on increasing diagnoses of STIs: a perspective from Bristol, UK. *Sexually Transmitted Infections* 2020;Accepted for Publication
36. Malterud K, Siersma VD, Guassora AD. Sample Size in Qualitative Interview Studies:Guided by Information Power. *Qualitative Health Research* 2016;26(13):1753-60. doi: 10.1177/1049732315617444
37. Reeves S, Kuper A, Hodges BD. Qualitative research methodologies: ethnography. *BMJ* 2008;337 doi: 10.1136/bmj.a1020
38. Emerson R, Fretz R, Shaw L. Writing ethnographic fieldnotes. Chicago and London: University of Chicago Press 1995.
39. Braun V, Clarke V. One size fits all? What counts as quality practice in (reflexive) thematic analysis? *Qualitative Research in Psychology* 2020:1-25. doi: 10.1080/14780887.2020.1769238
40. Public Health England. Guidance on the collection and reporting of data for GUMCAD, the surveillance system for sexually transmitted infections (STIs) in England. 2019 [Available from: https://www.gov.uk/guidance/gumcad-sti-surveillance-system
41. Horner P, Blee K, O'Mahony C, et al. 2015 UK National Guideline on the management of non-gonococcal urethritis. *International journal of STD & AIDS* 2016;27(2):85-96. doi: 10.1177/0956462415586675 [published Online First: 2015/05/24]
42. Turner KME, Zienkiewicz AK, Syred J, et al. Web-Based Activity Within a Sexual Health Economy: Observational Study. *J Med Internet Res* 2018;20(3):e74. doi: 10.2196/jmir.8101
43. Mohiuddin S, Gardiner R, Crofts M, et al. Modelling patient flows and resource use within a sexual health clinic through discrete event simulation to inform service redesign. *BMJ Open* 2020;10(7):e037084. doi: 10.1136/bmjopen-2020-037084 [published Online First: 2020/07/10]
44. Whitlock GG, Gibbons DC, Longford N, et al. Rapid testing and treatment for sexually transmitted infections improve patient care and yield public health benefits. *International journal of STD & AIDS* 2018;29(5):474-82. doi: 10.1177/0956462417736431 [published Online First: 2017/10/24]
45. Keizur EM, Goldbeck C, Vavala G, et al. Safety and Effectiveness of Same-Day Chlamydia trachomatis and Neisseria gonorrhoeae Screening and Treatment Among Gay, Bisexual, Transgender, and Homeless Youth in Los Angeles, California, and New Orleans, Louisiana. *Sex Transm Dis* 2020;47(1):19-23. doi: 10.1097/olq.0000000000001088 [published Online First: 2019/11/07]
46. Natoli L, Guy RJ, Shephard M, et al. Public health implications of molecular point-of-care testing for chlamydia and gonorrhoea in remote primary care services in Australia: a qualitative study. *BMJ Open* 2015;5(4):e006922. doi: 10.1136/bmjopen-2014-006922 [published Online First: 2015/04/30]
47. Girometti N, Delpech V, McCormack S, et al. The success of HIV combination prevention: The Dean Street model. *HIV medicine* 2021 doi: 10.1111/hiv.13149 [published Online First: 2021/07/30]

For peer review only - http://bmjopen.bmj.com/site/about/guidelines.xhtml

48. Dixon-Woods M, McNicol S, Martin G. Ten challenges in improving quality in healthcare: lessons from the Health Foundation's programme evaluations and relevant literature. *BMJ Quality & Safety* 2012;21(10):876-84. doi: 10.1136/bmjqs-2011-000760
49. Boog K, Heanue J, Kumar V. Implementing integrated sexual and reproductive healthcare in a large sexual health service in England: challenges and opportunities for the provider. *BMJ Sexual & Reproductive Health* 2019;45(2):164-67. doi: 10.1136/bmjsrh-2018-200090
50. Gollop R, Whitby E, Buchanan D, et al. Influencing sceptical staff to become supporters of service improvement: a qualitative study of doctors' and managers' views. *Quality and Safety in Health Care* 2004;13(2):108-14. doi: 10.1136/qshc.2003.007450
51. The Kings Fund. Understanding NHS financial pressures: How are they affecting patient care?, 2017.
52. World Health Organisation. Emergence of multi-drug-resistant Neisseria gonorrhoeae—threat of global rise in untreatable sexually transmitted infections, 2012.
53. Public Health England. PHE infectious diseases strategy 2020 to 2025, 2019.
54. Town K, Harris S, Sánchez-Busó L, et al. Genomic and Phenotypic Variability in Neisseria gonorrhoeae Antimicrobial Susceptibility, England. *Emerg Infect Dis* 2020;26(3):505-15. doi: 10.3201/eid2603.190732
55. Zienkiewicz AK, Verschueren van Rees N, Homer M, et al. Agent-based modelling study of antimicrobial-resistant Neisseria gonorrhoeae transmission in men who have sex with men: towards individualised diagnosis and treatment. *Sex Health* 2019;16(5):514-22. doi: 10.1071/sh18235 [published Online First: 2019/09/03]
56. Toskin I, Govender V, Blondeel K, et al. Call to action for health systems integration of point-of-care testing to mitigate the transmission and burden of sexually transmitted infections. *Sex Transm Infect* 2020;96(5):342-47. doi: 10.1136/sextrans-2019-054358 [published Online First: 2020/04/04]

For peer review only

For peer review only - http://bmjopen.bmj.com/site/about/guidelines.xhtml

Table 1: Quotes on Coherence and Cognitive Participation	
**Coherence**	**Cognitive participation**
**Staff enthusiasm and concerns**<br>It all sounded quite exciting and I was quite, not excited, but it was like, this is really good, the first one in the UK and, you know, this will be excellent, so I was quite open minded about it... lowering the use of antibiotics and fewer invasive procedures for women. (Nurse 17, T3)<br>It's an exciting opportunity, um, but also there's a bit of, um, er, you know, nerves I suppose about how it will actually be, actually run from day one, really, and how it would go (Public Health 14, T2)	**Disengaged staff**<br>It was very much the higher-up staff that kind of organised it all and they're not really the ones that are going to be doing the actual work, so I think it's really important to include clinical staff of all levels, kind of when it's getting near to it and you know, really explain, and have their opinions and thoughts on, you know, how it's gonna work (Nurse 16, T2)
**Reduced patient anxiety**<br>Results on the same day would be amazing, yeah, no doubt about that yeah, because there's always quite an – well, for me, it's like an anxious wait otherwise. Yeah, that sense of not knowing and actually "how do I manage my sex life in case anything comes back positive?" Yeah, quick results can definitely make a big difference (Mike, didn't use rapid STI service)<br>The thing that would encourage me to test more regularly, [is] if the whole thing [time spent in clinic] could be done in a shorter time slot for me (Ben, used rapid STI service)	**Barriers to engagement**<br>[Clinic managers need to] canvass people's feelings about it because I don't know that we do that very well. I think we just crack on. We don't say 'how was it for you that first week did you cope? Did you keep your head above water?' (Nurse 10, T1)

For peer review only

Avoiding inappropriate antibiotics prescriptions	Inadequate preparation
With antibiotics I'm really – they really mess with my stomach. I really feel really sick whenever I do take them. So, I'm just pro not taking them for that reason alone. Yeah, like – I try to think about the bigger picture of the world and stuff…but I think about my own stomach more than the wider world. So, yeah, I'd – I'm always pleased to like not have to do any unnecessary drugs (Andy, results by text)<br>I just think I'd hate to kind of like take something that I didn't have and then if I ever got it again it doesn't work - so it's kind of like half society view half personal view (Harry, follow-up appointment)	We were told on the Wednesday that it was supposed to be starting on the Monday and we were like all a bit shocked thinking 'well hang on a minute what about the training? We'll look really, really stupid in front of patients' (Administrative staff 6, T1)<br>There was always talk about what it [Panther] could do, never talk about how it's going to function. Even at the last minute, the week before it was meant to start [Lead Consultant] came to me and goes, 'This is what I think the pathway is. Can you make notes on it?' Nobody was really clear about what happened. [Project manager] had sent a PowerPoint around and it was embedded in a way that… some staff who aren't maybe familiar with how embedded links and things work [- couldn't open it]. When the meeting came around, they said, 'Has everyone read the email about the Pathways?' half of the people went, 'What email?' …, it just wasn't presented to us in a clearest way. (Health Adviser 1, T2)

For peer review only

Table 2: Changes along the (walk-in) male patient pathway			
	**Before rapid STI service**	**Rapid STI service**	
**Registering at reception**	All walk-in patients allocated an appointment in order of queue – may have to come back later that day.	Receptionist triages each patient to appropriate pathway referring to guide/pathway	
		Patients not eligible for rapid STI service are given an appointment for later that day and continue on old pathway	Rapid STI service-eligible patients wait after registering to be called up.
	Triage form used to register patients.	Triage form amended to be gender neutral and to make categories clearer.	
**Seeing a clinician/ health adviser**	At first appointment	First appointment very brief– reduced history taking. Unless uncomplicated vaginal discharge.	
	MSM and all patients needing partner notification/ risk reduction/safer sex advice see health adviser	High-risk patients (MSM or new to service) see health adviser at initial appointment.	
		Symptomatic men see doctor/trained nurse only at follow-up not drop-off, usually on same day.	
**Providing samples**	Urine and swabs: taken at time of consultation. Self-taken if asymptomatic. If symptomatic: clinician taken swab for microscopy (to detect NGU and gonorrhoea) and gonorrhoea culture; NAAT self-taken	Self-sample drop-off - Urine and swabs NAAT self-sampled in toilets, putting samples through a hatch to the laboratory. Instructions are on posters in the toilet and from nursing staff/NAs. Gonorrhoea culture taken by clinician on return only if NAAT positive. Swab for microscopy to detect NGU if NAAT-negative (see text above).	
	Blood samples taken by doctor/nurse/nursing assistant (NA)	Blood samples taken by doctor/nurse/NA	
**Tests results**	All STI test results in 2 -3 weeks	Chlamydia and gonorrhoea processed on Panther - results within 48 hours. Others still 2-3 weeks	
	If negative, text sent	Results by text if asymptomatic and negative.	
	If positive, HA phones patient to discuss result and arrange treatment (unless already received presumptive treatment).	Results given at follow up appointment with clinician if symptomatic or asymptomatic positive.	
**Treatment**	Treat presumptively	Wait for results before treating	
	Treat at first (only) appointment	Treat at follow-up appointment	
**Notified partners**	Treated immediately	Only treat on positive results (if sexual contact was >2 weeks; otherwise treat immediately)	

For peer review only - http://bmjopen.bmj.com/site/about/guidelines.xhtml

**Table 3: Collective action quotes**
**Designing and documenting new processes**<br>We need to be really clear about what we're doing when they drop-off [patients drop-off samples], what we're doing when they come back, what are we going to do about contraception, which questions are okay to leave out of the proforma. ..for consultants because we have a lot of experience and because we're used to making decisions then I think we can [unclear] a bit and we can be flexible and can you know think about the individual patient. But for nurses who work a lot to PGD's [guidelines] and like to have clear guidance. And some of the juniors as well, who will be quite new - you know they've just been changed. Because it's going to be bewildering and chaotic you know it's doesn't feel good when there's chaos on the shop floor. (Doctor 3, T1)
**Flexibility in pathways**<br>It's a case by case situation and it does help to have helpful medical staff that have been willing to make an exception. (Administrative staff 18, T2)<br>I think the health advisers also are more able to …know the guidelines but in some situations know that you have to approach things differently, … for me personally if I was seeing someone and they kind of said 'actually I have got this dis[ease]' - you know, real clear symptoms, you know, 'and I'm really fed up with it', I'd be more inclined to say 'okay then let's get you treated…I think you can have your general thing of saying to someone 'look you know come back in the afternoon' but if you've got someone who's kind of 'actually no but I've had these symptoms for two days I've really had enough of it'. (Health Adviser 13, implementation group member, T2)
**Guidelines**<br>It's a work in progress but the problem is as the pathway evolves then the guideline will change again…because this is so rapidly moving actually, I don't think I really want to do a guideline. So it's kind of hard to have a guideline anyway but we need some kind of guidance. (Doctor 3, T1)
**Teething issues**<br>It was chaos, the first few weeks were chaos. Reception didn't know what they were doing… there was hundreds of patients around the reception, we didn't know what we were doing, so yeah, it was chaos, but it has slowly got better. (Nurse 17, T2)<br>The waiting area fills up and people are filling out the Panther triage forms on windowsills. After a while [clinic coordinator] tells receptionists on Panther desk that he had given out 16 forms. Once they get to 10 people booked for Panther returner pathway they need to go check with the laboratory regarding further capacity. (Observation notes, Reception area)<br><br>Two reception staff were unsure whether one person should be panther/same day or walk-in due to the information provided on the form. Staff consulted with person entering data on computer. They checked whether person was returning for results/treatment. They explained to the patient that a new system is in place, so they want to make sure they do the best for him. (Observation notes, Reception area)
**Understaffing**<br>It's been very stressful for staff and I think it has been an enormous amount of work for the implementation group, that I think in the private industry you'd be given huge amounts of time, whereas we virtually squeezed it in amongst everything else we've done, but that's just the NHS. (Doctor 11, T2)<br>Administrative staff / reception team has three staff vacancies, and today there are two members of clinic staff off sick – one clinician, and one Administrative staff (clinic coordinator). The clinician would have been doing sample drop-off, and walk-in, so have had to reduce slots for both until they get confirmation of clinical capacity from clinicians when they arrive. (Observation notes, Reception area)
**Changing ingrained behaviours**

For peer review only

For peer review only - http://bmjopen.bmj.com/site/about/guidelines.xhtml

It's been quite hard on staff and obviously there's a lot of – you know, if you've been doing something the same way for 10/20/maybe 30 years, that's quite a massive change for people. (Nurse 21, T3)
**Changes to clinician contact**<br>We've actually ended up seeing a lot more complicated or complex patients, at least that's how it feels. The easy patients get siphoned off quite quickly and that means that more patients [can be seen], especially the complex patients, which the nurses are less able to deal with and require a lot more consultant supervision. I think there has been a general feeling in the department that the consultant cover job is busier than it ever was before. (Doctor 11, T2)
**Challenges of changes at reception**<br>[Receptionists] were worried that they were looking like they didn't know what they were doing, because it was new and they weren't quite sure. So I think it took a, it was a lot to ask for them all really because it was a big change, but it is just that keep reminding everybody that actually, in the long term, you will get it, and it's much better for other patients once it's in place. (Administrative staff 12, implementation group member, T2)<br>Reception staff on male desk refresh the panther decision pathway together using A4 sheet. Female staff member commented that she always has to double check the process. Reception staff discussed male staff member's confusion about eligibility for Panther. (Observation notes, Reception area)
**Concern about shorter consultations**<br>It was a huge change, because, we, it is quite a detailed consultation. We have been told time and time again that 'oh you need to ask patients about domestic violence, ask the women about female genital mutilation, you need to do this'. Then all of a sudden, they are saying, 'no, don't ask any of these things', it's like aargh! (Nurse 17, T2)<br>It does sometimes feel if I'm absolutely honest a little bit less than a level three service, you know, people are just coming in and dropping off a sample. I know that's possibly better use of our time, but it seems a little bit spurious to call it level three. (Nurse 10, T1)
**Perceived patient views on waiting for treatment**<br>I think the major anxiety that patients have is around not being treated immediately and not being treated necessarily as a contact of infection and anxiety around that. I often find that with a bit of educating that that is overcome and my major impression is that patients really appreciate it. (Doctor 11, T2)<br>I haven't had anybody who's been absolutely, you know, anti about it but there have been a couple of people who I've thought 'I'm going to treat you mate, I'm not going to wait on results' do you know what I mean? ... they're anxious, they've maybe got another partner, a regular partner, who they don't want to infect, which, you know, I can see the reasoning behind that. But I think, you know, once that kind of idea has got out amongst our regular clientele I think it will be a lot easier. (Nurse 10, T1)<br>Four young men approach the door together. [Name] lets them know that he has just 2 forms/slots left at present, and suggests that he gives these to them on the basis of who came through the door first – these two seem pleased, and head in with their forms. He asks the other two to wait here for a minute and they seem OK with this. He tells them there is a new service which means they can give people results/treatment faster, and this is why things are different. He asks them to give him a yes/no answer as to whether they have any symptoms. When they say no he says that it is probably not worth them waiting as they are unlikely to be seen today, and that they could come back another morning for when the doors first open. They seem to find this acceptable. (Observation notes, front door)
**Benefit of evaluation process**<br>Staff member commented that it was helpful to have an outside voice (research team) feeding back, because sometimes when you are within the structure you can be shouting stuff and nobody hears you. (Observation notes)

For peer review only - http://bmjopen.bmj.com/site/about/guidelines.xhtml

Table 4: Reflexive monitoring quotes
**Success**
When you speak at the national [sexual health] meetings, people, it's a bit of a no-brainer, what we're supposed to do, and people are amazed that we've been able to introduce it [rapid STI service] cost-neutrally. Because when you look at the point-of-care systems which other people are researching, it's… more expensive, so we've adopted an innovative approach. (Doctor 19, T3)
**Quality of care**
The person I saw was really brilliant, like, yeah. I felt really comfortable… I really felt like I could ask anything I want and felt sort of safe (Andy, results by text1)
**Benefits**
It was the immediacy and the kind of reassurance that … if something was positive that you would be able to treat it straightaway. (Harry, follow-up appointment)
They're [patients] very happy. I mean, who wouldn't be? You find out the same day that you have got chlamydia and you can start your treatment. I mean that is brilliant. (Nurse 17, T3)
Dr 11: One of the advantages that we hoped would come out of introducing Panther would be that we would attract more high-risk people, because it would be seen as an attractive place to come and test, and also that it would free up staff time so that we could spend more time with risk reduction etc.
Interviewer: Do you think that is happening? Or is it not there yet?
Dr 11: I think it has started to happen, I don't think that's only down to Panther, I think that's down to some other stuff like PrEP and things like that as well. I feel like the cohort of patients that we see is increasingly complex. (Doctor 11, T2)
I think the most positive things are seeing your symptomatic patients with knowing what is going on with them. You know what infection they have, you know what treatment they require or if they don't have anything you can then take the time to discuss that. (Doctor 11, T2)
I think it's [rapid service implementation] made the staff more able to deal with change [to telephone clinics], because they had undergone experience of change with Panther pathways over the past 12-18 months…. Yes, it probably made it smoother and more efficient. (Doctor 22,T4)
**Suggested improvements**
The main issues that have arisen have been when the [Panther] machine fails and that can be pretty catastrophic (laughs), just because you have booked slots and patients come back and you don't, you can't even tell them whether they have chlamydia or gonorrhoea and they've, kind of, come with that expectation. (Doctor 20, T3)
Interviewer: If you had an imaginary clinic who were going to set out on this path, what would your advice be to them overall? With the knowledge that you've now gathered from your experience, is there a way that you could help them?
Dr 11: I think preparation, preparing all of the documents that support your staff on a day-to-day basis, clarifying your communication pathways, giving your lead clinicians adequate time in their work plans to do all of that. I would definitely support it, I think it's definitely been a major benefit. (Doctor 11, T2)
It has been a big change for all staff working, and it's difficult to know whether there was any way of realising some of the things we hadn't realised. I don't think we could have done. I think they were literally just things of implementation that have caused some additional tweaks required - and that in itself has been stressful because it's been the realisation of what are we doing in this scenario and not being quite prepared for it. (Administrative staff 12, T2)

For peer review only - http://bmjopen.bmj.com/site/about/guidelines.xhtml

The thing that I found most interesting is the communication difficulties in amongst the staff and how difficult that has been, having an implementation group that I think represents most of the groups that it's impacted upon and the difficulty that the messages just have not got to the clinic floor, and that's an on-going issue. (Doctor 11, T2)

For peer review only

For peer review only - http://bmjopen.bmj.com/site/about/guidelines.xhtml

Pathway stage	Usual care pathway	Rapid pathway
**Reception**	- Walk-in patients' queue as clinic opens and appt are allocated on first-come-first-served basis	Patients are triaged to eligible for rapid STI service or not eligible.<br>• Eligible – Registered<br>• Non-eligible - continue along old pathway
**Clinical review**	Appt with clinician, full history taken.	Brief history taken at initial appt<br>MSM referred to Health Adviser<br>If follow up appt needed then full history taken at that time
**Sample testing**	Asymptomatic: Self-taken samples<br>Symptomatic: Clinician-taken samples for NAAT, GC culture and microscopy (as appropriate)	Self-taken NAAT testing **only** at initial appt<br>GC cultures taken **only** if GC positive on NAAT<br>Samples taken for microscopy only if NAAT negative and symptomatic
**Test results**	All test results available after approximately 10-14 days	Symptomatic: results available within the same day (triggering same/next day follow-up appt)<br>Asymptomatic: within 48 hours
**Treatment**	Patients treated syndromically prior to NAAT results.<br>Patient return for infection-specific treatment when test results are known (10-14 days)	Infection-specific treatment provided same/next day based on rapid test results
**Partner notification**	Started when positive test results returned (at 10-14 days)	Immediately commenced at same/next day follow-up appt

Figure 1. Overview of rapid pathway service redesign (MSM = Men who have sex with men; NAAT = Nucleic Acid Amplification Test; GC = Gonorrhoea Culture, appt = appointment)

For peer review only - http://bmjopen.bmj.com/site/about/guidelines.xhtml

Unity rapid-results service study

Service user interview topic guide v6 (third round)

1. *Introduction and background*

- Thanks, introduce self, re-state purpose of the interview, structure, recording, right to withdrawal
- If participant aged 16 or 17 – explain safeguarding policy and implications for confidentiality
- Check for questions
- Consent: Face to face – record in writing on consent form. Phone – go through consent form & audio record.
- Any relevant demographics not already recorded on the reply slip.

2. *Accessing the service*

- What prompted your visit this time? *(Check if specific concerns and symptoms vs regular check-up)*
- Have you been to the clinic before? *(If so when / how many times?)*
- How did you know about clinic/what did you know in advance about what might happen?
    - How did this affect your decisions / plans? What else would you like to know and how?
- What were you expecting from the service on this occasion? *(Including expectations of treatment, wait etc)*
- How easy was it for you to fit accessing service into your life? *(e.g. take time off, travel etc.)*
    - How acceptable was this, and does it depend on reason for attendance?

3. *Sample drop off phase*

- Did you see a clinician at your first visit? Did you have to wait to be seen? *(How long, was this OK?)*
- What happened when you were called in? *(Info given, any procedures, opportunity to ask questions etc.)*
- Did you take your own samples?
    - How was it? Was it easy to understand how to take them? (*Females – dry swab)*
    - How did you find the process for dropping off your samples?
    - If you have experience of having a clinician take samples before, which would you prefer, and why?
- Females – was contraception discussed?
    - If yes: Raised by you (on form/face to face) or clinician? *(if wanted but not on form why not?)*
        - If advice/services required how were these provided/arranged? *(during apt/follow up?)*
        - Was this acceptable? Was there anything you would have liked to be different?
    - If no: Would you have liked to/was there an opportunity to discuss?
- Were you given information on what tests were for what, and on what would happen next, and was this easy to understand?
- Was there any other information you would have liked at this point?
- On your first visit, did you leave without getting any treatment – was this OK? If not, why not?

4. *Getting results and any treatment or follow up*

For peer review only

- Tell me about receiving your test results *(e.g. wait? How received? In batches? Clarity? How felt about?)*
    - Was this how you were expecting to receive your results? If not, what were your expectations?
    - How would you prefer to receive your results?
- What were the results of your tests?
    - Did this affect your views of the service? *(e.g. would service be more / less acceptable if circumstances different?)*
- Did you have a consultation with a member of staff (apart from drop-off)? Face to face/phone/both?
    - Were your results available at the time of the consultation?
    - How did you find the consultation? *(Including quality of interaction / amount of time with clinician/able to ask questions)*
- Was any treatment or follow up (further tests, further appointments, partner notification) arranged?
- How acceptable would/did you find waiting until follow up appointment for your results (that PM / next day) - to receive treatment? *(instead of being given treatment straight away 'just in case')*
    - Why was this acceptable / not acceptable *(probe re pros/cons, links to reason for attendance)*
    - If not volunteered: Is AMR something which concerns you, and would this influence preferences?
- Would/does this new rapid results service encourage you to test more regularly? Why?
- Were you given all the advice or support you wanted?
- Did you have an opportunity to talk to someone (about any concerns/for advice) during your visit?

5. *Overall view of the service*

- Overall what do you think has worked well with your visits to Unity?
- What could have improved your experience? Are there any other changes that you could suggest?
- Did the service meet your expectations? Did it provide what you needed?
- Can you think of any positive/negative impacts of organising the service this way? *(e.g. anything off-putting, or more likely to use?)*
- If you have had experience of using sexual health services before the rapid results service started, how did your experience of this service compare? *(Was there anything you preferred about previous service?)*
- What would prompt you to choose this rapid service if you saw it advertised? *(e.g. AMR concerns, quick results, fewer invasive tests?)* Or what explanation of change to service would be acceptable / persuasive to you?

6. *Any other issues*

- Any other issues the participant would like to raise? Is there anything important I have not asked you about?

Thank them for their time and check preferences regarding receipt of summary of study findings.

For peer review only

University Hospitals Bristol NHS Foundation Trust · unity Sexual Health — Be Informed. Be Safe. Be In Control. · University of BRISTOL

Unity rapid results service study

Staff interview topic guide (phase 4)

1. *Introduction and background*

- Thanks, introduce self, re-state purpose of the interview, structure, recording, right to withdrawal.
- Check for questions
- Consent: Phone – go through orally and audio record.

Background information – job/role? How long in this role? Any relevant previous roles/experience? What has your role been in the service changes made in response to COVID-19? Interviewed previously?

From what we understand the Unity service is primarily telephone based now. Walk me through what happens if someone needs a sexual health check. Can you describe the service now we're doing social distancing due to COVID-19?

- Interactions with service users?
- Administration
- Video vs telephone
- Laboratory work
- Interactions /relationships with colleagues?
- Workload?
- Use of panther – drop-off samples and follow-up with patients and accessing treatment
- Postal testing kit requests – use Panther machine now??
- Other?

2. *Coherence*

What do you think about telephone clinics? What is good/bad about them?

3. *Cognitive participation*

What training and support did you and/or others receive for running telephone clinics?
Were you involved in the design or delivery of the training?
How engaged were you in the training?
What was good/bad about the training?

4. *Collective action*

How are telephone clinics working?

- Triage process / involvement of reception staff
- Type / mix of service users seen e.g. symptomatic / asymptomatic, complexity, first/follow up etc.
- Number and duration of consultations
- Content of consultations and history taking
- Number / type of physical examinations you are carrying out?
- How is the process for obtaining samples working? (postal kits through panther machine)
- Getting treatment to patients (Paddy mentioned pick-ups from Boots chemist)
- How is follow-up process working?

How did you find making these changes? Positive or negative? Difficult or easy?
Do you and others in the team feel confident/skilled to do telephone triage?
What is working well? (advantages of having panther machine to do tests in-house / straight forward vs more complex patients – determining who to see in person)
Are there any advantages to the new system? Using Panther for the postal kits (those requested online) - how was this facilitated?
What is more challenging? (not being able to see patients / straight forward vs more complex patients – determining who to see in person)

For peer review only - http://bmjopen.bmj.com/site/about/guidelines.xhtml

How are patients responding to the way the service is running now?
Which patients or presentations is this working well for?
Which patients or presentations is this more challenging for?
How do the service design changes impact on the most at risk/vulnerable groups? (service more or less accessible for these groups) E.g. those experiencing domestic violence or who do not have access to phones.
What has helped support the current service changes? (reduced patient numbers, experience of introducing rapid testing / software to link postal tests to patient records)
[IF NOT MENTIONED ABOVE: The service went through a big change introducing panther / rapid testing, do you think having been through that prepared you/ the service for the current situation?

5. ***Reflexive monitoring***

Since starting this new telephone service, has anything had to change to improve it?
How have staff been able to feed back issues which need improvements? Has the way this has been done changed / improved since the new rapid results service was introduced?
What would improve the current telephone service during COVID-19?
What do you think the service will look like after lockdown? (advantages of rapid testing) Continue using postal kits post-lockdown?
Comparing before the rapid-results service was introduced to now, how well is the clinic/are you set up for any potential ongoing social distancing measures in the future? How has introducing the rapid results service helped this? (service is already set up for self-sampling, sample drop off, text results, prescriptions from chemist)

6. ***Any other issues***

- Any other issues the participant would like to raise? Is there anything important I have not asked you about?

Thank them for their time and check preferences regarding receipt of summary of study findings.

For peer review only

For peer review only - http://bmjopen.bmj.com/site/about/guidelines.xhtml

Elements of rapid STI service implementation	
**What**	**Who should do this**
**Changes to documentation**	
Rewrite and sign-off treatment guidelines and SOPs when new processes are agreed.	Project leads, clinical lead, project implementation/operations team and clinical governance guideline group
Change the triage process and form.	Project operations team, to include reception staff
Consider changes to IT system/medical records system.	Project leads, consultant and project implementation team
**Implementation of the actual machine and process**	
Write business case for new rapid STI service and have it signed off by PHE.	Clinic lead, clinic manager, lead consultant, project manager, in collaboration with PHE.
Source the machine, find space for it (with waste disposal) and install it. Arrange insurance (including negotiations with PHE and legal teams)	Clinic lead, operations manager, lead consultant, PHE, nursing lead
Ensure IT systems allow direct transfer of data from Panther	Project lead, clinic manager
Pilot before implementing with all patients	All staff including reception teams
Write protocol for Panther outages	Project leads, in collaboration with PHE.
Quality assessment scheme /UKAS accreditation	PHE team
**Services**	
Consider impact on other services	Project operations team
Adjust clinic timetabling to accommodate rapid STI service appointments	Operations manager
Changing medical history forms and process to accommodate the new appointment structure	Project leads and implementation team
Changes to the IT coding	Clinic data manager, project lead, clinic manager, clinical lead (minor)
**Staff engagement, training and communication**	
Put together an implementation team, to oversee implementation, and put in place mechanisms for all staff to feedback to this team	Representative from each staff group and clinic manager.
Clarify communication pathways between all staff and the implementation team	Clinic Manager, project leads
Consider the impact on staff roles and workload and if staffing changes are therefore needed	Project operations team
Regular meetings for staff involved in the new service	Possible staff to include: project leads, HAs, consultants, nursing assistants, nurses, administrative staff, researchers, IT lead, clinic manager, data manager, chlamydia screening program team lead.
Staff training and regular updates at existing staff training sessions	Led by project leads, all staff to attend
Regular departmental meetings	Project leads and clinical lead
**Patient communication**	
Communicate changes to patients – write leaflets/posters/website	Project leads

For peer review only

COREQ (COnsolidated criteria for REporting Qualitative research) Checklist

A checklist of items that should be included in reports of qualitative research. You must report the page number in your manuscript where you consider each of the items listed in this checklist. If you have not included this information, either revise your manuscript accordingly before submitting or note N/A.

Topic	Item No.	Guide Questions/Description	Reported on Page No.
**Domain 1: Research team and reflexivity**			
Personal characteristics			
Interviewer/facilitator	1	Which author/s conducted the interview or focus group?	
Credentials	2	What were the researcher's credentials? E.g. PhD, MD	
Occupation	3	What was their occupation at the time of the study?	
Gender	4	Was the researcher male or female?	
Experience and training	5	What experience or training did the researcher have?	
Relationship with participants			
Relationship established	6	Was a relationship established prior to study commencement?	
Participant knowledge of the interviewer	7	What did the participants know about the researcher? e.g. personal goals, reasons for doing the research	
Interviewer characteristics	8	What characteristics were reported about the inter viewer/facilitator? e.g. Bias, assumptions, reasons and interests in the research topic	
**Domain 2: Study design**			
Theoretical framework			
Methodological orientation and Theory	9	What methodological orientation was stated to underpin the study? e.g. grounded theory, discourse analysis, ethnography, phenomenology, content analysis	
Participant selection			
Sampling	10	How were participants selected? e.g. purposive, convenience, consecutive, snowball	
Method of approach	11	How were participants approached? e.g. face-to-face, telephone, mail, email	
Sample size	12	How many participants were in the study?	
Non-participation	13	How many people refused to participate or dropped out? Reasons?	
Setting			
Setting of data collection	14	Where was the data collected? e.g. home, clinic, workplace	
Presence of non-participants	15	Was anyone else present besides the participants and researchers?	
Description of sample	16	What are the important characteristics of the sample? e.g. demographic data, date	
Data collection			
Interview guide	17	Were questions, prompts, guides provided by the authors? Was it pilot tested?	
Repeat interviews	18	Were repeat inter views carried out? If yes, how many?	
Audio/visual recording	19	Did the research use audio or visual recording to collect the data?	
Field notes	20	Were field notes made during and/or after the inter view or focus group?	
Duration	21	What was the duration of the inter views or focus group?	
Data saturation	22	Was data saturation discussed?	
Transcripts returned	23	Were transcripts returned to participants for comment and/or	

For peer review only

Topic	Item No.	Guide Questions/Description	Reported on Page No.
		correction?	
**Domain 3: analysis and findings**			
Data analysis			
Number of data coders	24	How many data coders coded the data?	
Description of the coding tree	25	Did authors provide a description of the coding tree?	
Derivation of themes	26	Were themes identified in advance or derived from the data?	
Software	27	What software, if applicable, was used to manage the data?	
Participant checking	28	Did participants provide feedback on the findings?	
Reporting			
Quotations presented	29	Were participant quotations presented to illustrate the themes/findings? Was each quotation identified? e.g. participant number	
Data and findings consistent	30	Was there consistency between the data presented and the findings?	
Clarity of major themes	31	Were major themes clearly presented in the findings?	
Clarity of minor themes	32	Is there a description of diverse cases or discussion of minor themes?	

Developed from: Tong A, Sainsbury P, Craig J. Consolidated criteria for reporting qualitative research (COREQ): a 32-item checklist for interviews and focus groups. *International Journal for Quality in Health Care*. 2007. Volume 19, Number 6: pp. 349 – 357

Once you have completed this checklist, please save a copy and upload it as part of your submission. DO NOT include this checklist as part of the main manuscript document. It must be uploaded as a separate file.

For peer review only
